# Norlignans and Phenolics from *Curculigo capitulata* and Their Neuroprotection Against Glutamate-Induced Oxidative Injury in SH-SY5Y Cells

**DOI:** 10.3390/molecules29235648

**Published:** 2024-11-28

**Authors:** Xueru Wang, Wei Ma, Ying Wang, Fucai Ren, Kaijin Wang, Ning Li

**Affiliations:** Anhui Key Laboratory of Bioactivity of Natural Products, School of Pharmacy, Anhui Medical University, Hefei 230032, China; 19855867686@139.com (X.W.); mw421553449@sina.com (W.M.); 18326039565@163.com (Y.W.); renfucai@ahmu.edu.cn (F.R.); wkjahla@163.com (K.W.)

**Keywords:** *Curculigo capitulata*, chemical constituent, norlignans, phenolics, neuroprotective activity, Nrf2/HO-1, BDNF

## Abstract

The herb *Curculigo capitulata* (Lour.) Ktze is widely distributed in southern and southwestern China. The *Curculigo* genus and its primary chemical constituents exhibit remarkable antidepressant activities. To investigate the chemical constituents and potential health benefits of *C. capitulata*, a phytochemical study was conducted. In this study, seven new compounds (capitugenin A–G), including three new norlignans (**1**–**3**), a new chalcone dimer (**4**), a new hemiacetal (**5**), two novel pyrrolidine-based compounds (**6** and **7**), including one identified as a natural product (**7**), and nineteen known compounds (**8**–**26**), were isolated from *C*. *capitulata.* The chemical structures and absolute configurations of Compounds **1**–**7** were elucidated via comprehensive spectroscopic data analyses. The neuroprotective effects of Compounds **1**–**26** against glutamate-induced cell death were tested in the human neuroblastoma cell line SH-SY5Y. Compounds **1**, **3**, **6**, **8**, **11,** and **17** showed significant neuroprotective effects, with protection rates ranging from 29.4 to 52.8% at concentrations ranging from 5 to 40 μM. Western blot analysis indicated that Compound **3** exerted a protective effect by regulating the expression of Nrf2/HO-1.

## 1. Introduction

Neurodegenerative diseases (NDs), such as Alzheimer’s disease, Parkinson’s disease, and amyotrophic lateral sclerosis, are characterized by progressive dysfunction and loss of neurons due to specific neurological impairments [1,2]. Glutamate (Glu), the most abundant excitatory neurotransmitter in the central nervous system (CNS), plays a crucial role in synaptic communication and neuronal signaling. In conditions such as Alzheimer’s, Parkinson’s, and Huntington’s diseases, there is an abnormal accumulation of Glu in the extracellular space of the brain [3,4,5]. Glu accumulation is the primary factor responsible for excessive reactive oxygen species (ROS) generation, which can interact with biomolecules to generate harmful byproducts, such as peroxides and aldehydes, ultimately damaging the cellular structure and function [6,7,8].

Under normal conditions, cells maintain an antioxidant defence system that includes both enzymatic antioxidants, such as superoxide dismutase (SOD), and nonenzymatic antioxidants, such as glutathione and vitamins, to counteract excessive ROS. However, when this intrinsic antioxidant capacity is compromised, oxidative stress (OS) occurs, leading to cellular apoptosis, a key factor in the pathogenesis of most neurodegenerative disorders [9,10].

The nuclear factor E2-related factor 2 (Nrf2) pathway is thought to be the most important pathway in the cell for protection against OS [11,12]. Nrf2 can regulate the expression of heme oxygenase 1 (HO-1), an endogenous antioxidant protein, to detoxify or scavenge oxidant species [13]. Therefore, Nrf2 plays an important role in antioxidative defence systems, and natural products that upregulate Nrf2 may be potential therapeutic agents for pathologies associated with ND.

**Figure 1:**
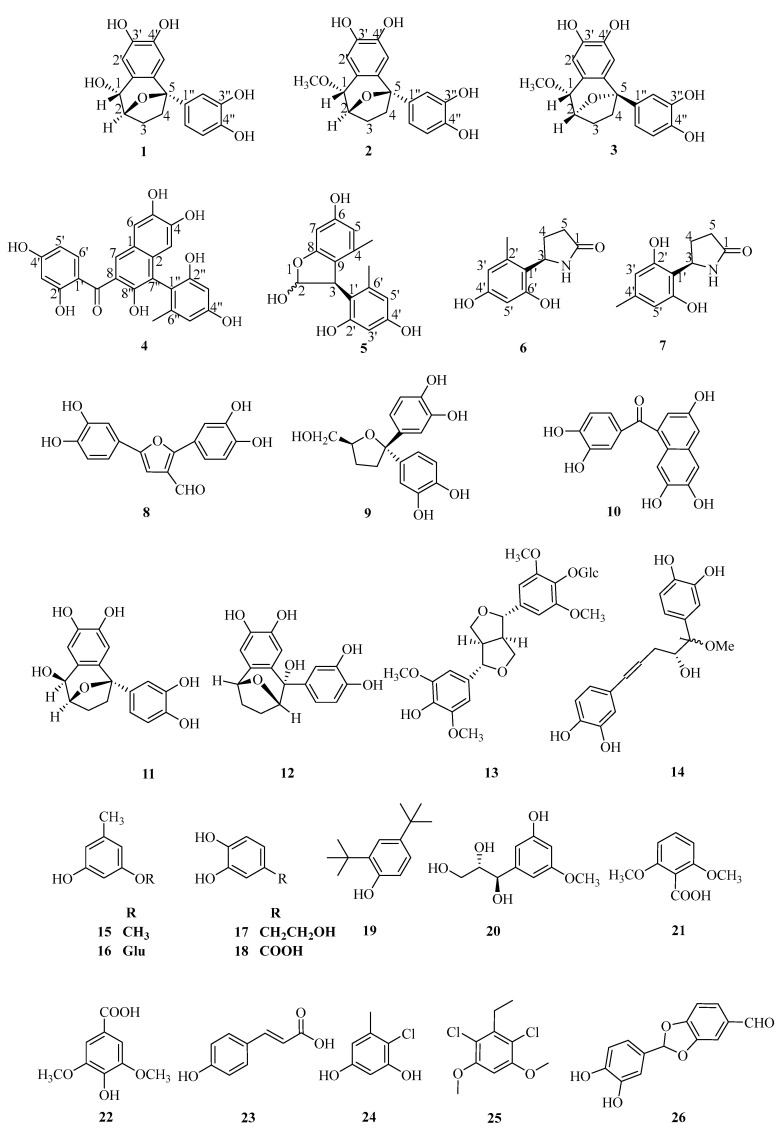
Structures of **1**–**26** isolated from *Curculigo capitulata*.

The genus *Curculigo* belongs to the Hypoxidaceae family. In traditional Chinese medicine, plants of the genus *Curculigo* have been used for centuries to treat conditions, such as impotence, limb weakness, and general fatigue [14,15]. Previous studies have demonstrated that the *Curculigo* genus and its primary chemical constituents exhibit remarkable biological activities, including antidepressant effects in chronic unpredictable mild stress (CUMS) rats and perimenopausal depression mice, anti-tumor effects against cervical cancer cells, and anti-osteoporotic effects in bone-related models [16,17,18,19,20]. Several norlignans and phenolic compounds demonstrate pronounced neuroprotective effects [21]. To identify the neuroprotective components in this plant, three new norlignans (**1**–**3**), a new chalcone dimer (**4**), a new hemiacetal (**5**), two novel pyrrolidine-based compounds (**6** and **7**), including one identified as a natural product (**7**), and nineteen known compounds (**8**–**26**) were isolated from *Curculigo capitulata* (Figure 1). Here, the isolation and structural elucidation of these compounds are described, and the protective effects of the isolated compounds on glutamate-induced oxidative damage in SH-SY5Y cells are discussed.

## 2. Results

### 2.1. Structural Elucidation of the Isolated Compounds

Compound **1** had a molecular formula of C_17_H_16_O_6_, as determined by its HRESIMS, ^1^H-NMR, and ^13^C-NMR data. The ^1^H-NMR data (Table 1) revealed signals characteristic of a 1,3,4,6-tetrasubstituted benzene ring [*δ*_H_ 6.79 (s, H-2′), 6.08 (s, H-5′)] and a set of peaks corresponding to an ABX system [*δ*_H_ 6.97 (d, *J* = 2.1 Hz, H-2″), 6.78 (d, *J* = 8.1 Hz, H-5″), and 6.89 (dd, *J* = 8.1, 2.1 Hz, H-6″)], two methylenes [*δ*_H_ 1.58 (m, H-3a), 2.24 (m, H-3b), and 2.16 (m, H-4a), 2.21 (m, H-4b)], two oxygenated methine protons [*δ*_H_ 4.61 (d, *J* = 8.1 Hz, H-2) and 4.10 (s, H-1)]. The ^13^C-NMR spectrum of Compound **1** revealed 17 carbon signals, which were attributed to four oxygenated olefinic carbons, five aromatic CH carbons, three aromatic quaternary carbons, and five aliphatic carbons, comprising one oxygenated quaternary carbon, two oxymethine carbons, and two methylene carbons.

**Figure 2:**
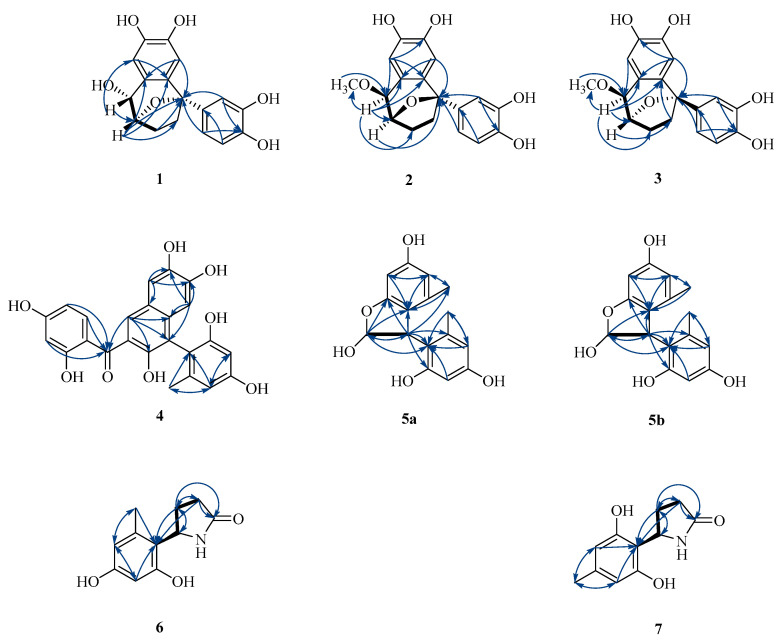
Key COSY (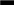), HMBC (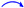) correlations of compounds **1**–**7**.

The structural fragment -CH(O)CH(O)CH_2_CH_2_C(O)- was deduced based on the ^1^H-^1^H COSY correlations between the C-2 methine proton and both the C-1 methine and C-3 methylene protons, as well as the HMBC correlations between H-2 and C-4 and between H-3 and C-5. The HMBC spectrum (Figure 2) revealed long-range couplings between H-2″ and H-6″ and C-5, suggesting the attachment of a 1,3,4-trisubstituted aromatic ring to C-5. The correlations (Figure 2) from H-1 to C-2′ and from H-5′ to C-5 established the connections of C-1–C-1′ and C-5–C-6′, respectively. The attachment of C-2 and C-5 to an oxygen atom was confirmed by the HMBC correlations between the proton at C-2 and the carbon at C-5, as well as the deshielded chemical shifts observed for C-2 and C-5 at *δ* 79.4 and *δ* 87.6, respectively (Table 1).

**Figure 3:**
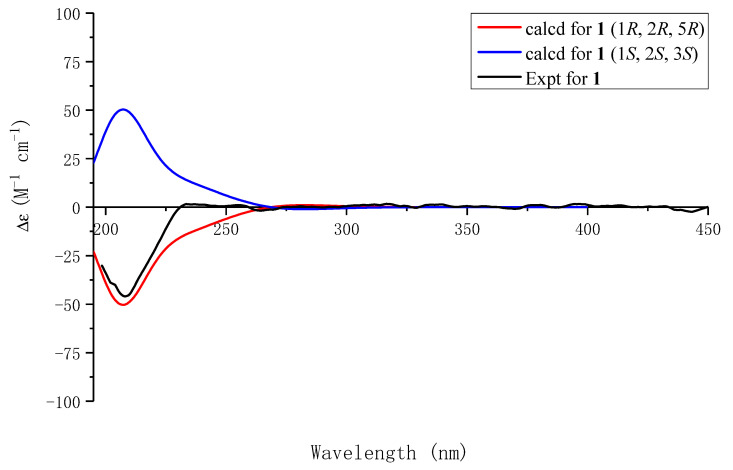
Experimental ECD spectrum of **1** and calculated ECD spectra of (1*R*, 2*R*, 5*R*)-**1** and (1*S*, 2*S*, 5*S*)-**1**.

The 1D and 2D NMR data (Table 1) of **1** were similar to those of **11**, sinensigenin C, except for the chemical shifts of H-1 (4.10 s) and H-2 (4.61 d, *J* = 8.1 Hz) in **1**, instead of H-1 (5.07 d, *J* = 5.1 Hz) and H-2 (4.55 t, 6.4, *J* = 6.2 Hz) in **11,** which indicated that Compound **1** was a conformational isomer of **11**. Due to the rigid architecture of the oxygen bridge, the relative configurations at C-2 and C-5 were fixed. The NOESY correlation of H-1 with H-2 was subsequently used to determine the *β* orientation of H-1–H-2 and C-2–C-5. The simulated ECDs were used to determine the absolute configuration, as evidenced by the experimental ECD curve matching one calculated (1*R*, 2*R*, 5*R*)-**1**, as shown in Figure 3. The structure of Compound **1**, namely, capitugenin A, was, thus, assigned as shown.

**Figure 4:**
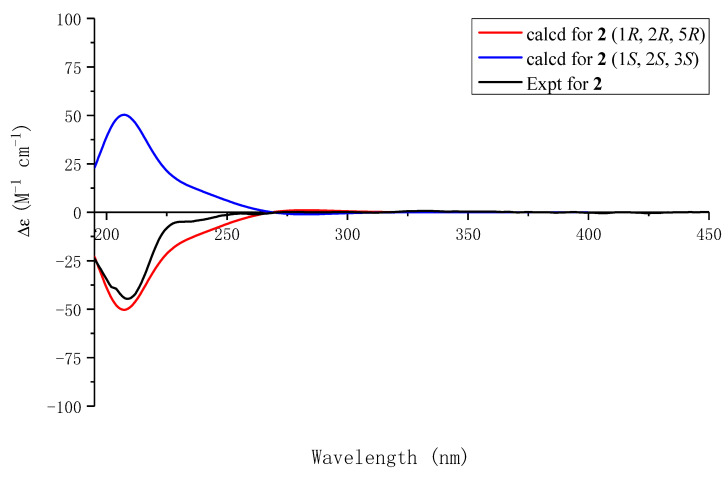
Experimental ECD spectrum of **2** and calculated ECD spectra of (1*R*, 2*R*, 5*R*)-**2** and (1*S*, 2*S*, 5*S*)-**2**.

Compound **2** had a molecular formula of C_18_H_17_O_6_, as determined by its HRESIMS, ^1^H-NMR, and ^13^C-NMR data. Compound **2** contained a methoxy group [(*δ*_H_ 3.54 s, H-OCH_3_) and (*δ*_C_ 57.5, C-OCH_3_)] in comparison with the NMR data of Compound **1** and was the methylated derivative of Compound **2** at C-1, as confirmed by the chemical shift of C-1 (*δ*_C_ 79.7 vs. 71.7) and the correlation from H-1 to C-OCH_3_ in the HMBC spectrum (Figure 2). The NOESY correlations of H-1 with H-2 indicated that Compound **2** retained the same stereochemical configuration at C-1 and C-2 as Compound **1**. Consequently, the absolute configuration of Compound **2** was established as 1*R*, 2*R*, and 5*R* based on the congruence between the calculated and experimental ECD spectra (Figure 4). The structure of Compound **2**, namely, capitugenin B, was, thus, assigned as shown.

**Figure 5:**
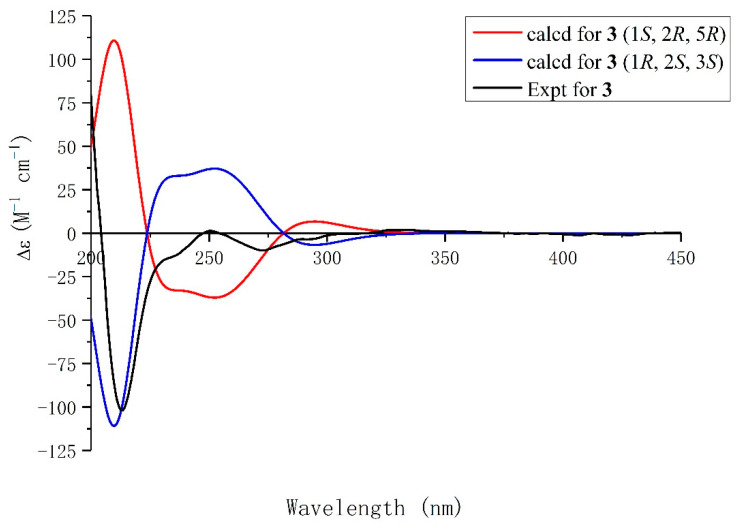
Experimental ECD spectrum of **3** and calculated ECD spectra of (1*S*, 2*R*, 5*R*)-**3** and (1*R*, 2*S*, 5*S*)-**3**.

Compound **3** had a molecular formula of C_18_H_17_O_6_, as determined by its HRESIMS, ^1^H-NMR, and ^13^C-NMR data. Analyses of the NMR data (Table 1) suggested that Compound **3** exhibited the same planar structure as Compound **2**, and the differences in the chemical shifts of C-1 (*δ*_C_ 80.4 vs. 79.7), C-3 (*δ*_C_ 26.3 vs. 23.1), C-4 (*δ*_C_ 38.5 vs. 39.6), C-5 (*δ*_C_ 86.5 vs. 87.3), C-1′ (*δ*_C_ 123.0 vs. 126.3), C-2′ (*δ*_C_ 117.8 vs. 114.9), C-6′ (*δ*_C_ 137.4 vs. 136.6) and C-6″ (*δ*_C_ 146.3 vs. 145.9) suggested that Compound **3** was a conformational isomer of Compound **2**. The experimental spectrum of Compound **3** was consistent with the calculated ECD spectrum of (1*R*, 2*S*, 5*S*)-**3** (Figure 5). The structure of Compound **3**, namely, capitugenin C, was, thus, assigned as shown.

Compound **4** had a molecular formula of C_24_H_18_O_8_, as determined by its HRESIMS, ^1^H-NMR, and ^13^C-NMR data. A detailed analysis of the 1D and 2D NMR data (Table 2) of **4** revealed the existence of four sets of substituted phenyl rings. The two peaks at *δ*_H_ 7.02 (s, H-3) and 6.54 (s, H-8) were attributed to the 1,2,4,6-tetrasubstituted aromatic group assigned to ring B of unit I of the chalcone dimer. The doublet of doublets at *δ*_H_ 7.17 (dd, *J* = 8.3, 2.1 Hz, H-5′), the ortho-coupled doublet at *δ*_H_ 6.86 (d, *J* = 8.3 Hz, H-6′), and the meta-coupled doublet at *δ*_H_ 7.30 (d, *J* = 2.1 Hz, H-3′) collectively indicated the presence of a trisubstituted aromatic system, corresponding to ring A of unit I in the chalcone dimer. Additionally, two meta-coupled doublets at *δ*_H_ 6.27 (d, *J* = 2.3 Hz, H-3″) and *δ*_H_ 6.24 (d, *J* = 2.3 Hz, H-5″) were characteristic of a 1, 2, 4, 6-tetrasubstituted aromatic ring, which was attributed to ring B of unit II in the dimeric structure. The connection between the two chalcone units was clearly established through the HMBC correlations observed between H-7/C-8″ and H-3/C-8″ in the spectrum. These signals were typical of a debenzoyl dimeric chalcone-type structure. The structure of Compound **4**, namely, capitugenin D, was, thus, assigned as shown.

Compound **5** had a molecular formula of C_16_H_16_O_5_, as determined by its HRESIMS, ^1^H-NMR, and ^13^C-NMR data. The 1D NMR spectrum revealed binate resonances of protons and carbons at a 1:1 ratio, which indicated that this compound exists in solution as a mixture of two stereoisomers. Structural analysis of one of the configurations (5a) of Compound **5** was then performed.

The ^1^H NMR spectrum (Table 3) displayed characteristic signals corresponding to two sets of 1,3,4,6-tetrasubstituted benzene rings at *δ*_H_ 6.00 (d, *J* = 2.0 Hz, H-5), 6.02 (d, *J* = 2.5 Hz, H-7), 6.19 (d, *J* = 2.5 Hz, H-3′), and 5.94 (d, *J* = 2.5 Hz, H-5′). Additionally, signals for two methyl groups were observed at *δ*_H_ 1.72 (s, 4-CH_3_) and *δ*_H_ 1.62 (s, 6′-CH_3_), a methylene group at *δ*_H_ 4.87 (d, *J* = 3.4 Hz, H-3), and a hemiacetal proton at *δ*_H_ 5.69 (dd, *J* = 6.7, 3.4 Hz, H-2). The ^13^C-NMR spectrum of Compound **5a** showed 16 carbon signals, which were attributed to two methyl groups, two methylenes, and eight quaternary carbons, including four oxygenated sp^2^ carbons.

**Figure 6:**
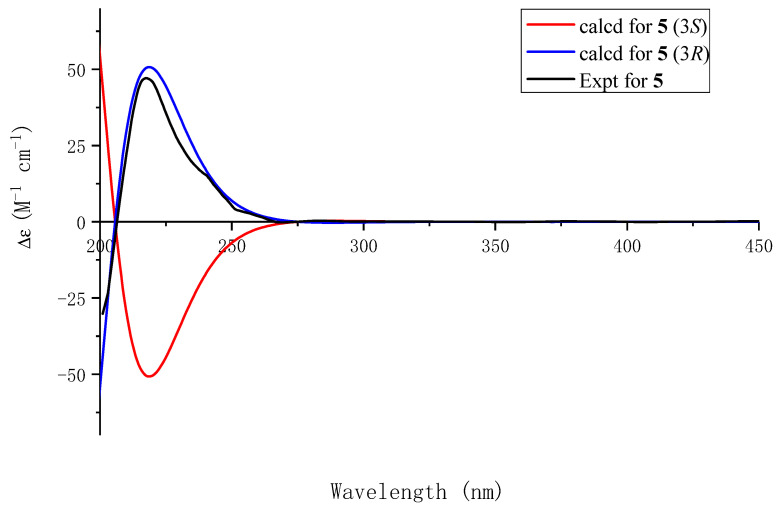
Experimental ECD spectrum of **5** and calculated ECD spectra of (3*R*)-**5** and (3*S*)-**5**.

The presence of a hydroxydihydrofuran ring was confirmed by the ^1^H-^1^H COSY correlations between H-2 and H-3, as well as the interaction between the free hydroxyl group [*δ*_H_ 7.10 (d, *J* = 6.7 Hz, -OH)] and C-2. Additionally, the HMBC correlations of H-2 with C-3, C-8, and C-9 and of H-3 with C-2, C-8, and C-9 further substantiated this structure, establishing the connections of O1-C2 and C-3-C-9. The HMBC spectrum (Figure 2) demonstrated long-range couplings between C-2′ and C-6′ with H-3, indicating that the 1,3,4-trisubstituted aromatic ring was connected to C-3. The simulated ECD spectrum of (3*S*)-**5** agreed with the experimental spectrum (Figure 6). The structure of Compound **5**, capitugenin E, was, thus, assigned as shown.

Attempts to separate this mixture consistently failed, suggesting that Compound **5** may undergo epimerization in solution, a common phenomenon for hemiacetals [22,23,24].

**Figure 7:**
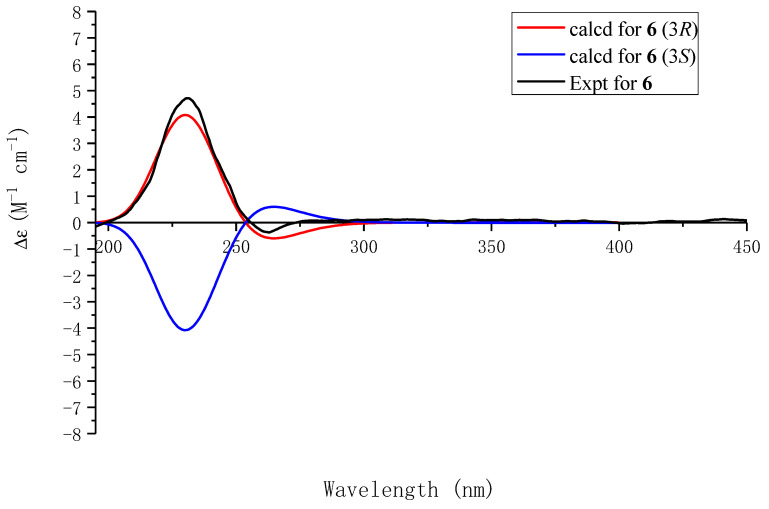
Experimental ECD spectrum of **6** and calculated ECD spectra of (3*R*)-**6** and (3*S*)-**6**.

Compound **6** had a molecular formula of C_11_H_13_NO_3_, as determined by its HRESIMS, ^1^H-NMR, and ^13^C-NMR data. The ^1^H NMR and ^13^C NMR spectra revealed characteristic resonances of pyrrolidin-2-one, including two methylenes [*δ*_H_ 2.23 (m, H-4a), 2.40 (m, H-4b), 2.43 (m, H-5a), 2.56 (m, H-5b)], a methine group [*δ*_H_ 5.16 (dd, *J* = 8.2, 5.8 Hz, H-3)], a conjugated ketocarbonyl group (*δ*_C_ 181.3, C-1), an ABX system [*δ*_H_ 6.15, d (*J* = 2.4 Hz, H-3′), 6.10, d (*J* = 2.4 Hz, H-5′)], and a methyl group [*δ*_H_ 2.26, s] (Table 4). The HMBC correlations between H-3 and C-2′ and C-6′ confirmed the connection of the 1,2,4,6-tetrasubstituted phenyl ring to the C-3 position of the pyrrolidin-2-one core. The simulated ECD spectrum of (3*S*)-**6** agreed with the experimental spectrum (Figure 7). The structure of Compound **6**, namely, capitugenin F, was, thus, assigned as shown.

**Figure 8:**
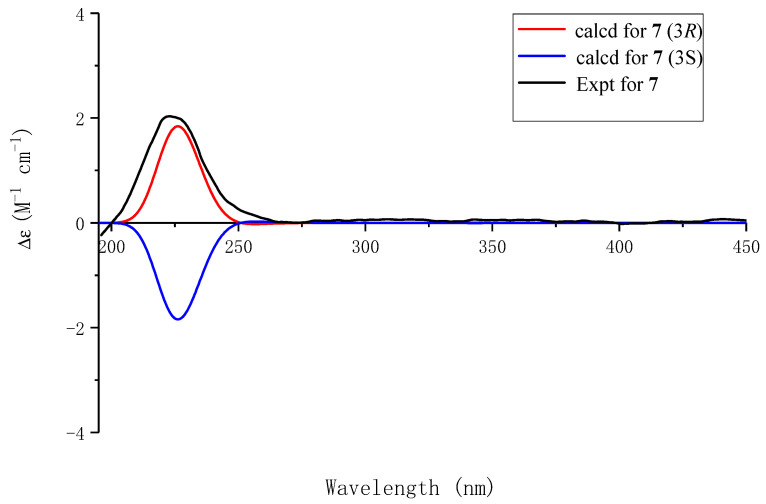
Experimental ECD spectrum of **7** and calculated ECD spectra of (3*R*)-**7** and (3*S*)-**7**.

Compound **7** had a molecular formula of C₁₁H₁₃NO₃, as determined by its HRESIMS, ^1^H-NMR, and ^13^C-NMR data, which were identical to those of Compound **6**. Combining the signals of aromatic protons in the ^1^H NMR spectrum with only nine carbon signals in the ^13^C spectrum (Table 4), it is possible that the 1, 2, 4, 6-tetrasubstituted benzene ring in **7** is symmetrical. The structure of **7** was confirmed by the correlation between methyl protons and C-3′/5′ in the HMBC spectrum. The simulated ECD spectrum of (3*R*)-**7** agreed with the experimental spectrum (Figure 8). The structure of Compound **7**, namely, capitugenin G, was, thus, assigned as shown.

The known compounds (**8**–**26**) were identified by comparing their experimental NMR spectral data with those in the literature. These compounds were identified as crassifogenin A (**8**) [25], breviscapin B (**9**) [26], crassifogenin B (**10**) [27], sinensigenin C (**11**) [28], sinensigenin A (**12**) [29], (+)-syringaresinol-O-β-D-glucopyranoside (**13**) [30], (1R, 2R)-crassifogenin D (**14**) [31], orcinol (**15**) [32], orcinol glucoside (**16**) [32], 3,4-dihydroxyphenylethyl alcohol (**17**) [33], protocatechuic acid (**18**) [34], 2,4-di-tert-butylphenol (**19**) [35], threo-5-hydroxy-3,7 dimethoxyphenylpropane-8,9-diol (**20**) [36], 2,6-dimethoxy-benzioc acid (**21**) [37], syringic acid (**22**) [38], *p*-coumaric acid (**23**) [39], 2-chloro-3,5-dihydroxytoluene (**24**) [40], 2,6-dichloro-3,5-dimethoxybenzenemethanol (**25**) [36], and 2-(3,4-dihydroxyphenyl)-1,3-benzodioxole-5-carboxaldehyde (**26**) [41].

### 2.2. Neuroprotective Activity of Compounds (***1**–**26***)

**Figure 9:**
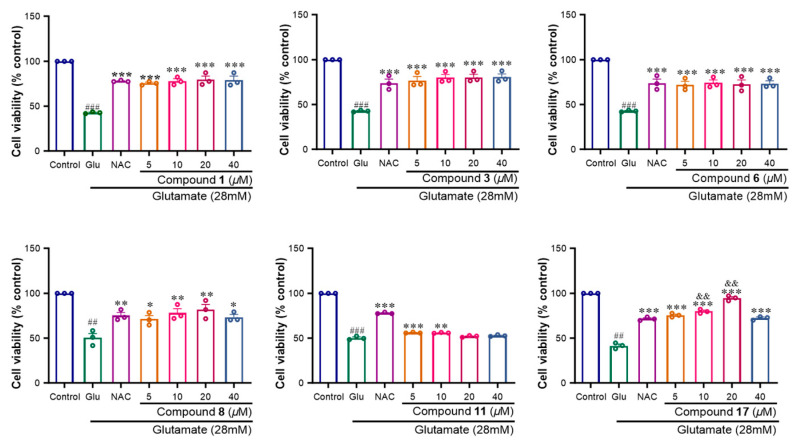
Effects of compounds **1**, **3**, **6**, **8**, **11** and **17** on glutamate-induced oxidative injury of SH-SY5Y cells. Results were obtained from three independent experiments, expressed by mean ± SEM. ## *p* < 0.01, ### *p* < 0.001 compared with the control group; * *p* < 0.05, ** *p* < 0.01, *** *p* < 0.001 compared with glutamate group; && *p* < 0.01 compared with the NAC group.

**Figure 10:**
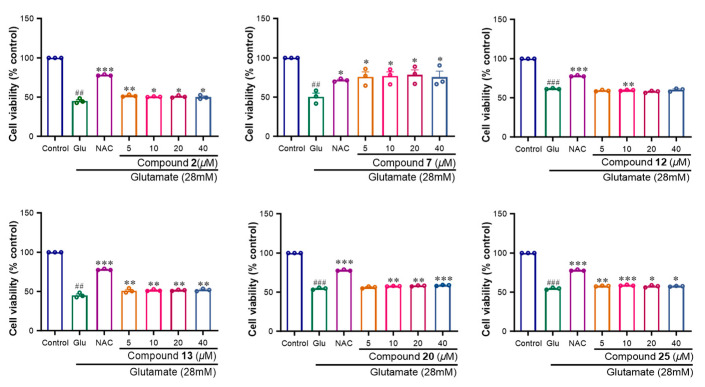
Effects of compounds **2**, **7**, **12**, **13**, **20** and **25** on glutamate-induced oxidative injury of SH-SY5Y cells. Results were obtained from three independent experiments, expressed by mean ± SEM. ## *p* < 0.01, ### *p* < 0.001 compared with the control group; * *p* < 0.05, ** *p* < 0.01, *** *p* < 0.001 compared with glutamate group.

The neuroprotective effects of Compounds **1**–**26** against Glu-induced oxidative injury in SH-SY5Y cells were evaluated via a CCK-8 assay. The results revealed that 28 mM Glu significantly reduced cell viability compared with that of the control group. Among the compounds, many displayed significant neuroprotective effects. Compounds **1**, **3**, **6**, **8**, **11** and **17** had significant protective effects, with protection rates ranging from 29.4 to 52.8% at concentrations ranging from 5 to 40 μM, whereas the positive control N-Acetylcysteine group (NAC) had a protection rate of 31.3% at 50 mΜ (Figure 9). Compounds **2**, **7**, **12**, **13**, **20** and **25** had moderate protective effects, with protective rates ranging from 5.63 to 30.5% at concentrations ranging from 5 to 40 μM (Figure 10). Compounds **4**, **5**, **9**, **10**, **14**, **15**, **16**, **18**, **19**, **21**, **22**, **23**, **24** and **26** exhibited no activity in this assay (Page S59). As shown in Figure 9 and Figure 10, Compound **3** among the norlignans had the most significant protective effect on SH-SY5Y cells, with no obvious cytotoxicity at 1, 10 or 100 μM. Thus, we chose Compound **3** for further study of its neuroprotective effects.

### 2.3. Effects of Compound ***3*** on Glutamate-Induced Intracellular LDH, SOD and ROS Release in SH-SY5Y Cells

**Figure 11:**
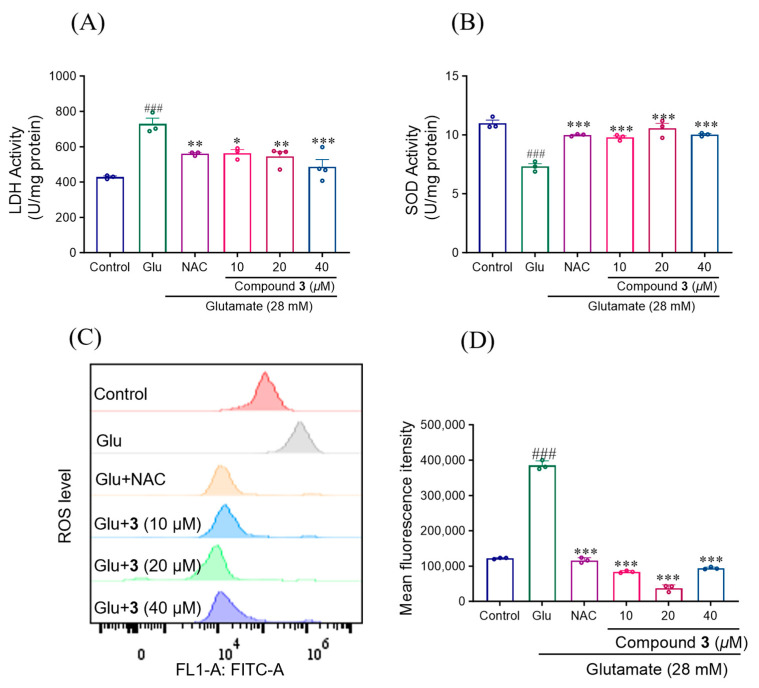
Effect of compound **3** on glutamate-induced oxidative stress in SH-SY5Y cells. (**A**,**B**) Activities of LDH and SOD were estimated according to commercial assay kits, respectively. (**C**,**D**) DCFH-DA staining was used to detect the effects of compound **3** on endogenous ROS levels in SH-SY5Y cells treated with glutamate. Results were obtained from three independent experiments, expressed by mean ± SEM. ### *p* < 0.001 compared with the control group; * *p* < 0.05, ** *p* < 0.01, *** *p* < 0.001 compared with glutamate group.

As shown in Figure 11, compared with the control group, the SOD activity of the Glu group decreased significantly (*p* < 0.01), the lactate dehydrogenase (LDH) content increased significantly (*p* < 0.01), and the oxidative damage effect was apparent. Compared with the Glu group, Compound **3** significantly increased SOD activity (*p* < 0.01) and reduced LDH content (*p* < 0.05, *p* < 0.01, *p* < 0.001) at concentrations of 10, 20 and 40 μM. The effects of Compound **3** on ROS levels were subsequently evaluated by DCFH-DA staining and flow cytometry (Figure 11). Compared with those in the control group, the ROS levels in the Glu group were significantly greater (*p* < 0.01), and Compound **3** significantly reduced the ROS levels (*p* < 0.01).

### 2.4. Effects of Compound ***3*** on the Nrf2/HO-1 and BDNF Downregulation Induced by Glutamate in SH-SY5Y Cells

**Figure 12:**
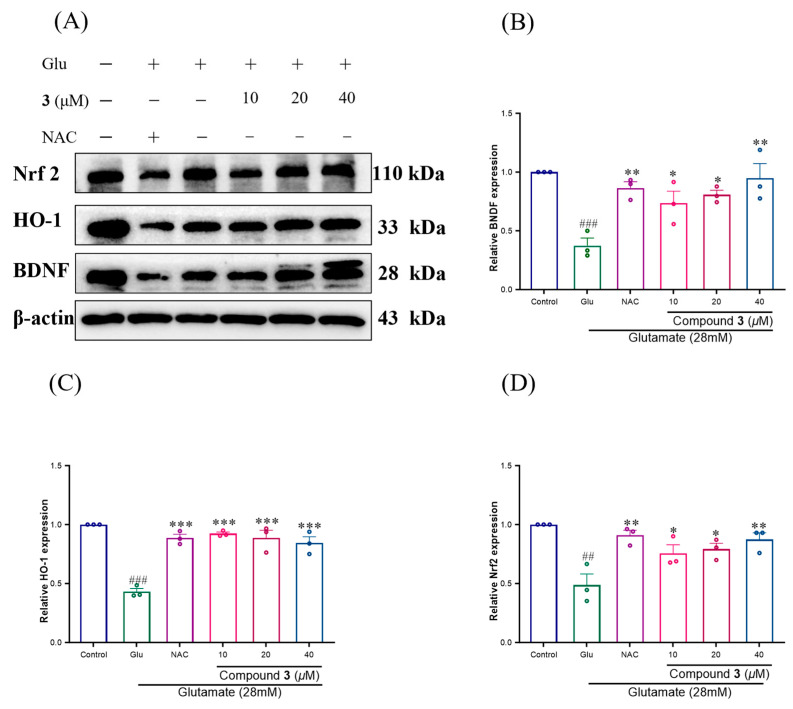
Compound **3** promote Nrf2/HO-1 and BDNF release and exert antioxidant protective activity. (**A**–**D**) Results were obtained from three independent experiments, expressed by mean ± SEM (compared to the control group ## *p* < 0.01, ### *p* < 0.001. Compared to the glutamate group, ** *p* < 0.01, * *p* < 0.05, *** *p* < 0.001).

To explore the protective mechanism of Compound **3** against Glu-induced SH-SY5Y damage, the protein expression of Nrf2/HO-1 and BDNF in SH-SY5Y cells was detected by Western blotting. Compared with that in the control group, the protein expression of Nrf2/HO-1 and BDNF in the Glu group was significantly lower, whereas Compound **3** significantly increased the expression of Nrf2/HO-1 and BDNF (Figure 12).

## 3. Discussion

OS plays a pivotal role in the development of NDs by generating free radicals that harm nerve cells and contribute to neurodegeneration [42]. In the mammalian central nervous system, a high Glu concentration causes OS and contributes to neuronal degeneration [7,8]. Consequently, researchers often use Glu to induce neuronal damage, facilitating investigations into neuroprotective effects and underlying molecular mechanisms for potential drug therapies.

*Curculigo orchioides* is reported to be rich in phenolic and norlignan compounds, which exhibit notable neuroprotective properties [21]. Similarly, *C*. *capitulata* and *Curculigo orchioides* both belong to the same genus and share highly similar chemical profiles. Given this chemical resemblance, we hypothesize that the primary constituents of *C*. *capitulata* may also possess neuroprotective activity.

In our research, three new norlignans (**1**–**3**), a new chalcone dimer (**4**), a new hemiacetal isomer mixture (**5**), a new pyrrolidine (**6**), a new pyrrolidine natural product (**7**), and nineteen known compounds (**8**–**26**) were isolated from *C*. *capitulata*. The neuroprotective effects of these compounds were assessed using a CCK-8 assay in Glu-induced SH-SY5Y cells.

A comprehensive analysis of the structure–activity relationships for Compounds **1**–**3** and **11**–**12** revealed that the presence of a hydroxy or methoxy group at the C-1 position may be more likely to exhibit pronounced neuroprotective activity (Fifure **1**, **9** and **10**). Similarly, hydroxy or methoxy substitutions at the C-3 or C-4 positions were also found to contribute substantially to their neuroprotective effects. Furthermore, the observed differences in the activities of Compounds **2** and **3** were attributed to distinct conformations at the C-2 and C-5 positions, highlighting the critical role of stereochemistry in determining their efficacy.

Exposure of SH-SY5Y cells to Glu resulted in cell membrane damage and apoptosis, leading to the release of LDH from the cytoplasm into the medium, which is indicative of cell injury. SOD is an important antioxidant enzyme in the body that can protect the integrity of cell membranes and reduce damage of peroxides.

Further investigations demonstrated that Compound **3** significantly reduced LDH release in Glu-damaged SH-SY5Y cells and enhanced intracellular SOD activity. These findings suggest that Compound **3** exerts neuroprotective effects by augmenting the activity of the endogenous antioxidant defense system.

OS is associated with the accumulation of high levels of reactive oxygen and reactive nitrogen species (RNS). During normal metabolism, cells produce a certain amount of ROS. However, the generation of excessive ROS plays a key role in the process of glutamate-induced neuronal cell death [43]. As shown in Figure 10, Compound **3** significantly inhibited intracellular ROS levels. These results provide evidence for the antioxidant effect of Compound **3**.

Recent studies have shown that the Nrf2 pathway plays a critical role in the regulation of many antioxidative stress/antioxidant and detoxification enzyme-encoding genes. HO-1, the main effector of Nrf2, is an important endogenous antioxidant protein that detoxifies or scavenges oxidant species in the cell to protect against oxidative stress [44,45,46,47]. Brain-derived neurotrophic factor (BDNF) is a small dimeric protein and a neurotrophic factor. The amount of BDNF and its receptors is reduced in the hippocampus of animal models of NDs and normally ageing animals, which may lead to synaptic and cell loss and memory loss [48,49].

Thus, we tested whether active norlignans induce Nrf2/HO-1 and BNDF expression-mediated protective effects. These results suggest that Compound **3** effectively prevented oxidative stress-induced neuronal death by maintaining the Nrf2/HO-1 pathway in SH-SY5Y cells.

## 4. Conclusions

In conclusion, we report 26 norlignans and phenolic compounds (**1**–**26**), including six new compounds (**1**–**6**) and a new natural product (**7**), which were isolated from *C*. *capitulata*. The complex stereo structures of these compounds were elucidated by extensive spectroscopic data analyses and comparisons of their experimental and calculated ECD spectra. Biologically, Compounds **1**, **3**, **6**, **8**, **11** and **17** had strong protective effects on glutamate-induced oxidative damage in SH-SY5Y cells, and Compounds **2**, **7**, **12**, **13**, **20** and **25** had moderate protective effects. Additionally, Compound **3** reduced the release of LDH and ROS in glutamate-damaged SH-SY5Y cells, increased the activity of intracellular SOD, and played a protective role by promoting the activation of the Nrf2/HO-1 and BDNF signaling pathway. Taken together, the results showed that norlignans from *C. capitulata* could be used as candidate compounds for further research and development as neuroprotective agents for the treatment of PD.

## 5. Materials and Methods

### 5.1. General Experimental Procedures

Optical rotation was measured using an Anton Paar MCP-150 polarimeter (Anton Paar, Graz, Austria). Infrared (IR) spectra were recorded using a Bruker IFS-66/S FT-IR spectrometer (Bruker, Karlsruhe, Germany). UV spectra were obtained using a Shimadzu UV-1500 spectrophotometer (Shimadzu, Kyoto, Japan). Further, 1D and 2D NMR spectra were recorded on an Avance III-600 spectrometer with TMS as an internal standard (Bruker, Fällanden, Switzerland). High-performance liquid chromatography (HPLC) separations were performed using a Thermo Scientific UltiMate3000 liquid chromatography system (Thermo, Waltham, MA, USA), and a Thermo Hypersil ODS column (250 × 10 mm, 5 μm; Thermo, Waltham, MA, USA) was used. Medium-pressure liquid chromatography (MPLC) was performed using a Biotage Dalton 2000 (Biotage, Uppsala, Sweden).

Column chromatography was performed using silica gel (200–300 mesh) and thin-layer chromatography (TLC) silica gel plates (Shanghai Honghu Biomedical Technology, Shanghai, China), Sephadex LH-20 (25–100 μM, Pharmacia, Uppsala, Sweden), C18 silica gel (50 μM, YMC, Kyoto, Japan) or middle chromatogram isolated gel (MCI, 75–150 μm, Mitsubishi, Tokyo, Japan).

### 5.2. Plant Material

The rhizomes of *Curculigo capitulata* were collected in Leshan, Sichuan Province, China, in July 2022. The species was authenticated by Prof. Kai-Jin Wang (School of Life Sciences, Anhui University, China), and a voucher specimen (20220708) was deposited in the School of Pharmacy at Anhui Medical University.

### 5.3. Extraction and Isolation

The dried rhizomes of *C. capitulata* (10 kg) were extracted with 95% ethanol at room temperature 3 times, filtered, and concentrated to obtain 1350 g of ethanol extract. The ethanol extract was separated via silica gel column chromatography (silica gel CC) and eluted with a gradient mixture of dichloromethane (CH_2_Cl_2_)/methyl alcohol (MeOH) (from 100:0 to 0:100) to obtain 3 fractions (A–C).

Fraction A (50 g) was further fractionated using silica gel CC (CH_2_Cl_2_/MeOH from 100:1 to 5:1), which yielded ten subfractions (Frs. A1–A10).

Fraction A1 (6 g) was subjected to MCI column chromatography (MeOH/H_2_O from 10% to 100%) to produce seven fractions (Fr. A1.1–A1.7).

Compounds **23** (4 mg) and **15** (4212 mg) were obtained from Fr. A1-1 (4670 mg) via MPLC (C18, MeOH/H_2_O 40:60). Fraction A1-4 (560 mg) was separated on a Sephadex LH-20 (CH_2_Cl_2_/MeOH 1:1) column and purified by high-performance liquid chromatography (HPLC) (MeOH/H_2_O 40:60, 2.0 mL/min) to obtain Compound **25** (3 mg, *t_R_* 17.9 min).

Fraction A6 (3 g) was separated by MPLC (C18, MeOH/H_2_O from 10% to 100%) and purified on a Sephadex LH-20 column (MeOH) to yield Compounds **24** (12 mg) and **17** (13 mg).

Fraction A9 (22 g) was subjected to a Sephadex LH-20 column (MeOH/H_2_O 10% to 100%) to yield nine fractions (A9.1–A9.9). Fractions A9-2 (186 mg), A9-3 (690 mg), and A9-9 (6 g) were separated by silica gel CC (CH_2_Cl_2_/MeOH 30:1 to 1:1) and purified by MPLC (C18, MeOH/H_2_O 10% to 100%) to yield Compounds **19** (7 mg), **22** (39 mg), **26** (6 mg) and **21** (939 mg).

Fraction B (36 g) was subjected to column chromatography on a silica gel column and eluted with CH_2_Cl_2_/MeOH (from 50:1 to 10:1) to yield two fractions (B1–B2). Further purification of Fr. B1 (10 g) using silica gel CC (CH_2_Cl_2_/MeOH from 20:1 to 1:1) afforded three main fractions (B1.1–B1.3). Fractions B1-1 (54 mg) and B1-2 (410 mg) were separated by MPLC (MeOH/H_2_O from 10% to 100%) to obtain Compounds **5** (9 mg), **8** (8 mg) and **4** (60 mg), respectively. Fraction B1-3 (1.5 g) was separated on a Sephadex LH-20 (CH_2_Cl_2_/MeOH 1:1) and silica gel CC (CH_2_Cl_2_/acetone 15:1) column to yield Compounds **7** (90 mg), **6** (110 mg) and **12** (9 mg).

Fraction B2 (6 g) underwent a sequential and detailed purification process, beginning with MPLC over a C18 gel (MeOH/H_2_O from 10% to 100%) to divide it into two fractions (B2-1–B2-2). Fraction B2-1 (1 g) was purified by silica gel CC (CH_2_Cl_2_/MeOH from 20:1 to 1:1) and semipreparative HPLC (acetonitrile/H_2_O 40:60, 2.5 mL/min) to yield Compounds **1** (11.0 mg, *t_R_* 17.3 min) and **3** (17.0 mg, *t_R_* 28.5 min). Fraction B2-2 (1.3 g) was separated on a Sephadex LH-20 column (CH_2_Cl_2_/MeOH 1:1) and purified by silica gel CC (CH_2_Cl_2_/MeOH 100:1) to yield Compounds **18** (151 mg) and **2** (12 mg).

Fraction C (50 g) was subjected to column chromatography (MCI, MeOH/H_2_O 10% to 100%) and further fractionated using Sephadex LH-20 (CH_2_Cl_2_/MeOH 1:1), yielding seven fractions (C1–C7). Fractions C1 (1.6 g) and C3 (1.2 g) were separated by silica gel CC (CH_2_Cl_2_/MeOH from 15:1 to 1:1) and purified by MPLC (ODS, MeOH/H_2_O from 10% to 100%) to yield Compounds **16** (1.2 g), **11** (30 mg), and **20** (3 mg). Fraction C4 (1 g) was subjected to a Sephadex LH-20 column (CH_2_Cl_2_/MeOH 1:1) and purified by MPLC (MeOH/H_2_O from 10% to 100%) to obtain Compound **10** (25 mg).

Fraction C5 (890 mg) was subjected to silica gel CC (CH_2_Cl_2_/MeOH from 15:1 to 1:1) and Sephadex LH-20 (MeOH) to produce two fractions (C5-1 and C5-2). Fraction C5-1 (89 mg) was purified by HPLC (MeOH/H_2_O 22:78, 2.5 mL/min), and Compounds **9** (3 mg, *t_R_* 15.6 min), **13** (30 mg *t_R_* 27.5 min) and **14** (12 mg *t_R_* 44.5 min) were obtained.

### 5.4. Spectroscopic Data

**Capitugenin A** (**1**): Amorphous powder; ${\left[\alpha \right]}_{D}^{25}$-37.33 (c 0.1, MeOH); UV (MeOH) *λ*_max_ (log *ε*) 190 (4.04), 204.5 (4.18) nm, 279 (3.19) nm; IR *ν*_max_: 3442.2, 1631.4, 1592.2, 1384.2, 1350.3, 765.9 cm^−1^; for ^1^H (600 MHz, MeOH) and ^13^C (175 MHz, MeOH) NMR data, see Table 1; (+)-HRESIMS *m*/*z* 339.0880 [M + Na]^+^ (calcd. for C_17_H_16_NaO_6_ 339.0845).

**Capitugenin B** (**2**): Amorphous powder; ${\left[\alpha \right]}_{D}^{25}$-40.27 (c 0.1, MeOH); UV (MeOH) *λ*_max_ (log *ε*) 191 (3.98), 203 (4.13) nm, 283 (3.10) nm; IR *ν*_max_: 3434.3, 2951.6, 1652.1, 1581.7, 1518.7, 1453.2, 1293.6, 1092.6, 964.4, 879.6, 814.7, 783.1, 639.5 cm^−1^; for ^1^H (600 MHz, MeOH) and ^13^C (175 MHz, MeOH) NMR data, see Table 1; (+)-HRESIMS *m*/*z* 329.1030 [M − H]^−^ (calcd. for C_18_H_17_O_6_^−^ 329.1025).

**Capitugenin C** (**3**): Amorphous powder; ${\left[\alpha \right]}_{D}^{25}$-29.33 (c 0.1, MeOH); UV (MeOH) *λ*_max_ (log *ε*) 191.5 (4.11), 203.5 (3.99) nm, 279.5 (2.98) nm; IR *ν*_max_: 3406.8, 1649.4, 1580.5, 1518.6, 1452.1, 1258.4, 1092.8, 880.2, 815.0, 784.25 cm^−1^; for ^1^H (600 MHz, MeOH) and ^13^C (175 MHz, MeOH) NMR data, see Table 1; (+)-HRESIMS *m*/*z* 329.1030 [M − H]^−^ (calcd. for C_18_H_17_O_6_^−^ 329.1025).

**Capitugenin D** (**4**): pale yellow solid; ${\left[\alpha \right]}_{D}^{25}$+2.0 (c 0.1, MeOH); UV (MeOH) *λ*_max_ (log *ε*) 192 (4.36), 199 (4.13) nm, 236 (4.04), 282 (3.38) nm; IR *ν*_max_: 3424.4, 1631.3, 1591.1, 1384.1, 1350.2, 1269.7, 768.5 cm^−1^; for ^1^H (600 MHz, MeOH) and ^13^C (175 MHz, MeOH) NMR data, see Table 2; (+)-HRESIMS *m*/*z* 457.0869 [M + Na]^+^ (calcd. for C_24_H_18_NaO_8_ 457.0899).

**Capitugenin E** (**5**): Amorphous powder; ${\left[\alpha \right]}_{D}^{25}$-1.1 (c 0.1, MeOH); UV (MeOH) *λ*_max_ (log *ε*) 191.5 (4.01), 204 (3.97) nm, 282 (2.92) nm; IR *ν*_max_: 3423.8, 1598.4, 1468.8, 1383.6, 1349.4, 1313.2, 1242.3, 1207.6, 1144.4, 1124.8, 1010.3, 970.7, 837.6 cm^−1^; for ^1^H (600 MHz, MeOH) and ^13^C (175 MHz, MeOH) NMR data, see Table 3; (+)-HRESIMS *m*/*z* 311.0891 [M + Na]^+^ (calcd. for C_16_H_16_NaO_5_ 211.0895).

**Capitugenin F** (**6**): Amorphous powder; ${\left[\alpha \right]}_{D}^{25}$-1.6 (c 0.1, MeOH); UV (MeOH) *λ*_max_ (log *ε*) 206 (3.88), 285 (2.55) nm; IR *ν*_max_: 3597.5, 3414.4, 3205.6, 2983.0, 1651.6, 1606.5, 1513.7, 1474.9, 1400.8, 1336.9, 1283.1, 1226.1, 1166.5, 1145.9, 1092.8, 1057.9, 974.9, 841.0, 808.2, 651.7, 523.9, 500.7 cm^−1^; for ^1^H (600 MHz, MeOH) and ^13^C (175 MHz, MeOH) NMR data, see Table 4; (+)-HRESIMS *m*/*z* 230.0826 [M + Na]^+^ (calcd. for C_11_H_13_NNaO_3_ 230.0793).

**Capitugenin G** (**7**): Amorphous powder; ${\left[\alpha \right]}_{D}^{25}$-29.32 (c 0.1, MeOH); UV (MeOH) *λ*_max_ (log *ε*) 208 (3.09), 284 (2.46) nm; IR *ν*_max_: 3393.0, 2678.1, 1646.5, 1593.2, 1427.0, 1372.7, 1280.6, 1160.7, 1060.9, 984.9, 824.5, 641.0, 589.0, 514.8 cm^−1^; for ^1^H (600 MHz, MeOH) and ^13^C (175 MHz, MeOH) NMR data, see Table 4; (+)-HRESIMS *m*/*z* 230.0826 [M + Na]^+^ (calcd. for C_11_H_13_NNaO_3_ 230.0793).

### 5.5. ECD Calculations

The theoretical electronic circular dichroism (ECD) spectra for Compounds **1**, **2**, **3**, **5**, **6** and **7** were generated using density functional theory (DFT), as implemented in the Gaussian 16 software suite. First, a conformational search was conducted using the MMFF94 molecular mechanics force field, identifying conformers with energies within a 2.0 kcal/mol range. Subsequent optimization of these conformations was performed at the B3LYP/6-31g(d, p) level of theory. The ECD intensities for each conformer were then computed through time-dependent density functional theory (TDDFT) at the B3LYP/6-311+g(2d, p) basis set in a methanol solvent environment. Finally, the ECD spectra were plotted using OriginPro 2017 software.

### 5.6. Cell Lines and Cell Culture

The human neuroblastoma cell line SH-SY5Y was purchased from the Cell Bank of Wuhan Servicebio Technology (Servicebio, Wuhan, China). The cells were cultured in minimum essential medium (MEM), enriched with L-glutamine, D-glucose, and sodium pyruvate (Servicebio Wuhan, China). The medium was further supplemented with 10% foetal bovine serum (Rongye Biotechnology, Lanzhou, China) and antibiotics, including 100 units/mL penicillin and 100 µg/mL streptomycin (Kaiji Biotechnology, Nanjing, China). The cells were maintained in a humidified incubator at 37 °C with 5% CO_2_.

### 5.7. Cell Viability Assay

Cell viability was determined using a Cell Counting Kit-8 (CCK-8, Apexbio, Houston, TX, USA) assay [21]. SH-SY5Y cells were seeded at a density of 2 × 10^4^ cells per well in a 96-well plate, followed by treatment with sample concentrations of 5, 10, 20, and 40 µmol/L. The cells were incubated at 37 °C for 24 h. Subsequently, 10 µL of CCK-8 solution was added to each well, and the mixture was incubated for an additional 3 h. Cell viability was quantified using a microplate reader at 450 nm (PerkinElmer, Waltham, MA, USA). The percentage of cell viability was calculated using the formula [(Acompound − Ablank)/(Acontrol − Ablank) × 100%] [21].

### 5.8. Determination of SOD and LDH in Cultured Cells

SH-SY5Y cells were seeded in 6-well plates at 2.5 × 10^5^ cells/well for 24 h and treated with sample concentrations of 10, 20, and 40 µmol/L for 24 h. The concentrations of biomarkers, including SOD (Nanjing Jiancheng Bioengineering Institute, Nanjing, China) and LDH (Nanjing Jiancheng Bioengineering Institute, Nanjing, China), were measured using assay kits according to the manufacturer’s recommendations. The protein concentration was measured using a BCA protein assay kit (Elabscience, Wuhan, China).

### 5.9. Western Blot Analysis

Total protein was extracted from SH-SY5Y cells at low temperatures using RIPA lysis buffer (Beyotime, P0013B, Haimen, China). The protein concentration was quantified using a BCA protein assay kit (Elabscience, E-BC-K318-M, Houston, TX, USA). Protein samples were separated via SDS-PAGE and subsequently transferred onto polyvinylidene fluoride (Merck Millipore, PR05505, Burlington, MA, USA) membranes. Following a 1–2 h blocking step in 5% skim milk, the membranes were incubated overnight at 4 °C with primary antibodies. The membranes were then washed three times with Tris-buffered saline containing 1% Tween-20 (Servicebio, G0004), followed by incubation with either rabbit or mouse secondary antibodies at room temperature for 1 h. Finally, the membranes were washed three additional times with TBST, and the protein bands were visualized using an enhanced chemiluminescence detection kit (Affinibody, AIWB-006, Wuhan, China), followed by analysis with ImageJ2 software.

### 5.10. Flow Cytometry

Flow cytometry was used to measure the ROS levels in SH-SY5Y cells. DCFH-DA was diluted to a final concentration of 10 μmol/L according to the instructions of the manufacturer for the ROS Assay Kit (Beyotime, S0033S). After cell digestion and collection, the cells were resuspended in diluted DCFH-DA at a concentration of 1 × 10^6^ to 2 × 10^7^ cells/mL and incubated for 20 min at 37 °C in a cell culture incubator. The cells were then washed three times, and the ROS levels were analyzed using a Cytek™ Aurora flow cytometer. Data analysis was conducted with FlowJo software (version 10.0).

## Figures and Tables

**Table 1:** ^1^H and ^13^C NMR (600, 150 MHz) data of compounds **1**–**3** in CD_3_OD.

Position	1		2		3	
	*δ*_C_	*δ*_H_ (*J* in Hz)	*δ*_C_	*δ*_H_ (*J* in Hz)	*δ*_C_	*δ*_H_ (*J* in Hz)
1	71.79	4.10 (1H, s)	79.74	4.75 (1H, d, 5.0 Hz)	80.44	3.86 (1H, s)
2	81.19	4.61 (1H, d, 8.1Hz)	76.09	4.86 (1H, ddd, 7.5, 5.0, 1.8Hz)	76.88	4.83(1H, d, 7.8Hz)
3	26.26	2.24 (1H, m); 1.58 (1H, m)	23.15	2.19 (1H, m); 2.03 (1H, m)	26.39	2.26 (1H, m); 1.57 (1H, m)
4	38.51	2.21 (1H, m); 2.16 (1H, m)	39.60	2.26 (2H, m)	38.51	2.22 (1H, m); 2.15 (1H, m)
5	86.44		87.39		86.57	
1′	125.73		126.39		123.06	
2′	115.65	6.79 (1H, s)	114.91	6.82 (1H, s)	117.86	6.74 (1H, s)
3′	145.89		145.33		145.96	
4′	145.53		145.01		145.47	
5′	112.83	6.08 (1H, s)	112.89	5.99 (1H, s)	112.83	6.09 (1H, s)
6′	136.79		136.63		137.49	
1″	135.58		135.68		135.41	
2″	116.34	6.97 (1H, d, 2.1 Hz)	116.20	6.87 (1H, d, 2.0 Hz)	116.28	6.93 (1H, d, 2.1 Hz)
3″	145.98		145.91		145.98	
4″	145.96		145.94		146.33	
5″	117.71	6.78 (1H, d, 8.1 Hz)	115.62	6.77 (1H, d, 8.2 Hz)	115.64	6.78 (1H, d, 8.1 Hz)
6″	120.26	6.89 (1H, dd, 8.1, 2.1Hz)	120.10	6.80, dd (8.2, 2.0)	120.17	6.85, dd (8.1, 2.1)
-OCH_3_			57.56	3.54 (3H, s)	56.23	3.53 (3H, s)

**Table 2:** ^1^H and ^13^C NMR data of compound **4** in DMSO.

Position	4	
	*δ*_C_	*δ*_H_ (*J* in Hz)
1	118.65	
2	130.47	
3	107.75	7.02 (1H, s)
4	144.5	
5	146.82	
6	107.38	6.54 (1H, s)
7	148.89	
8	120.67	
9	196.14	
1′	113.95	
2′	156.96	
3′	100.14	6.27(1H, d, 2.3 Hz)
4′	155.92	
5′	107.75	6.24 (1H, d, 2.5Hz)
6′	138.71	
1″	129.58	
2″	145.07	
3″	116.72	7.30 (1H, d, 2.1 Hz)
4″	150.95	
5″	123.55	7.17 (1H, dd,8.3,2.1Hz)
6″	115.23	6.86 (1H, d, 8.3 Hz)
7″	134.64	
8″	115.32	6.92 (1H, s)
-CH_3_	19.85	1.77 (3H, s)

**Table 3:** ^1^H and ^13^C NMR data of compound **5** in DMSO.

Position	5a		5b	
	*δ*_C_	*δ*_H_ (*J* in Hz)	*δ*_C_	*δ*_H_ (*J* in Hz)
1				
2	107.46	5.69 (1H, dd, 6.7, 3.4 Hz)	107.31	5.86 (1H, dd, 7.1, 4.6 Hz)
3	45.3	4.87 (1H, d, 3.4 Hz)	48.6	4.25 (1H, d, 4.6 Hz)
4	134.19		133.16	
5	108.26	6.00 (1H, d, 2.0 Hz)	107.68	5.90 (1H, d, 2.1 Hz)
6	157.5		156.92	
7	94.14	6.02 (1H, d, 2.5 Hz)	93.85	5.92 (1H, d, 2.1 Hz)
8	159.25		159.4	
9	118.51		119.07	
1′	115.77		115.37	
2′	155.99		156.57	
3′	99.83	6.19 (1H, d, 2.5 Hz)	101.09	6.02 (1H, d, 2.5 Hz)
4′	156.27		155.93	
5′	109.7	5.94 (1H, d, 2.5 Hz)	108.14	6.08 (1H, d, 2.4 Hz)
6′	138.29		137.68	
4-CH_3_	17.49	1.72 (3H, s)	17.49	1.67 (3H, s)
6′-CH_3_	18.76	1.62(3H, s)	20.59	2.27 (3H, s)

**Table 4:** ^1^H and ^13^C NMR data of compounds **6**–**7** in CD_3_OD.

Position	6		7	
	*δ*_C_	*δ*_H_ (*J* in Hz)	*δ*_C_	*δ*_H_ (*J* in Hz)
1	181.38		181.71	
3	53.46	5.16 (1H, dd, 8.2, 5.8 Hz)	50.38	5.37 (1H, dd, 9.3, 5.2 Hz)
4	27.14	2.23 (1H, m); 2.40 (1H, m)	26.95	2.26 (1H, m); 2.43 (1H, m)
5	32.3	2.43 (1H, m); 2.56 (1H, m)	32.24	2.36 (1H, m); 2.62 (1H, m)
1′	118.4		112.9	
2′	158.3		157.8	
3′	102.19	6.15 (1H, d, 2.4 Hz)	108.71	6.13 (1H, s)
4′	158.7		157.8	
5′	109.91	6.10 (1H, d, 2.4 Hz)	108.71	6.13 (1H, s)
6′	139.29		140.27	
CH_3_	20.35	2.26 (3H, s)	21.34	2.14 (3H, s)

## Data Availability

The data presented in this study are available in the main text of this paper and the Supplementary Materials.

## Supplementary Materials

The following supporting information can be downloaded at: https://www.mdpi.com/article/10.3390/molecules29235648/s1, Pages S1–S35: ^1^H NMR, ^13^C NMR, ^1^H-^1^H COSY, HSQC, HMBC, NOESY HRESIMS, IR and UV spectrum of compounds **1**–**7**. Pages S36–S54: ^1^H NMR, ^13^C NMR and ^13^C DEPT 135 of compounds **8**–**26**. Pages S55–S58: ^1^H and ^13^C NMR data of compounds **8**–**26**. Pages S58: Effects of compounds **4**, **5**, **9**, **10**, **14**, **15**, **16**, **18**, **19**, **21**, **22**, **23**, **24** and **26** on glutamate-induced oxidative injury of SH-SY5Y cells.
