# Supplementary material for: Norlignans and Phenolics from Curculigo capitulata and Their Neuroprotection Against Glutamate-Induced Oxidative Injury in SH-SY5Y Cells
Source: Molecules. 2024 Nov 28;29(23):5648. doi: 10.3390/molecules29235648 (PMC11643597; doi:10.3390/molecules29235648)

**Supplementary Materials**

# Norlignans and phenolics from *Curculigo capitulata* are protective against glutamate-induced oxidative injury via regulation of the expression of Nrf2/HO-1/BDNF in SH-SY5Y cells

**Xueru Wang [1], Wei Ma [1], Ying Wang, Fucai Ren, Kaijin Wang, Ning Li***

Anhui Key Laboratory of Bioactivity of Natural Products, School of Pharmacy, Anhui Medical University, Hefei 230032, China; 19855867686@139.com (X.W.); mw421553449@sina.com (W.M.); 18326039565@163.com (Y.W.); renfucai@ahmu.edu.cn. (F.R.); wkjahla@163.com (K.W.);

[1] These authors contributed equally to this work.

* Correspondence: 1993500019@ahmu.edu.cn (N.L.); Tel.: +86-551-6516115 (N.L.)

$^{1}$H-NMR spectrum of compound **1** (600 MHz, $CD_3OD$) ........ S1
$^{13}$C-NMR spectrum of compound **1** (151 MHz, $CD_3OD$) ........ S1
$^{1}$H-$^{1}$H COSY spectrum of compound **1** ........ S2
HSQC spectrum of compound **1** ........ S2
HMBC spectrum of compound **1** ........ S3
NOESY spectrum of compound **1** ........ S3
HRESIMS plot of compound **1** ........ S4
IR spectrum of compound **1** ........ S5
UV spectrum of compound **1** ........ S5
$^{1}$H-NMR spectrum of compound **2** (600 MHz, $CD_3OD$) ........ S6
$^{13}$C-NMR spectrum of compound **2** (151 MHz, $CD_3OD$) ........ S6
$^{1}$H-$^{1}$H COSY spectrum of compound **2** ........ S7
HSQC spectrum of compound **2** ........ S7
HMBC spectrum of compound **2** ........ S8
NOESY spectrum of compound **2** ........ S8
HRESIMS plot of compound **2** ........ S9
IR spectrum of compound **2** ........ S10
UV spectrum of compound **2** ........ S10
$^{1}$H-NMR spectrum of compound **3** (600 MHz, $CD_3OD$) ........ S11
$^{13}$C-NMR spectrum of compound **3** (151 MHz, $CD_3OD$) ........ S11
$^{1}$H-$^{1}$H COSY spectrum of compound **3** ........ S12
HSQC spectrum of compound **3** ........ S12
HMBC spectrum of compound **3** ........ S13
NOESY spectrum of compound **3** ........ S13
HRESIMS plot of compound **3** ........ S14
IR spectrum of compound **3** ........ S15
UV spectrum of compound **3** ........ S15
$^{1}$H-NMR spectrum of compound **4** (600 MHz, DMSO-$d_6$) ........ S16
$^{13}$C-NMR spectrum of compound **4** (151 MHz, DMSO-$d_6$) ........ S16
$^{1}$H-$^{1}$H COSY spectrum of compound **4** ........ S17
HSQC spectrum of compound **4** ........ S17
HMBC spectrum of compound 4 ........ S18

NOESY spectrum of compound **4** ........ S18
HRESIMS plot of compound **4** ........ S19
IR spectrum of compound **4** ........ S20
UV spectrum of compound **4** ........ S20
$^{1}$H-NMR spectrum of compound **5** (600 MHz, DMSO-$d_6$) ........ S21
$^{13}$C-NMR spectrum of compound **5** (151 MHz, DMSO-$d_6$) ........ S21
$^{1}$H-$^{1}$H COSY spectrum of compound **5** ........ S22
HSQC spectrum of compound **5** ........ S22
HMBC spectrum of compound **5** ........ S23
NOESY spectrum of compound **5** ........ S23
HRESIMS plot of compound **5** ........ S24
IR spectrum of compound **5** ........ S25
UV spectrum of compound **5** ........ S25
$^{1}$H-NMR spectrum of compound **6** (600 MHz, $CD_3OD$) ........ S26
$^{13}$C-NMR spectrum of compound **6** (151 MHz, $CD_3OD$) ........ S26
$^{1}$H-$^{1}$H COSY spectrum of compound **6** ........ S27
HSQC spectrum of compound **6** ........ S27
HMBC spectrum of compound **6** ........ S28
ROESY spectrum of compound **6** ........ S28
HRESIMS plot of compound **6** ........ S29
IR spectrum of compound **6** ........ S30
UV spectrum of compound **6** ........ S30
$^{1}$H-NMR spectrum of compound **7** (700 MHz, $CD_3OD$) ........ S31
$^{13}$C-NMR spectrum of compound **7** (151 MHz, $CD_3OD$) ........ S31
$^{1}$H-$^{1}$H COSY spectrum of compound **7** ........ S32
HSQC spectrum of compound **7** ........ S32
HMBC spectrum of compound **7** ........ S33
HRESIMS plot of compound **7** ........ S34
IR spectrum of compound **7** ........ S35
UV spectrum of compound **7** ........ S35
$^{1}$H-NMR spectrum of compound **8** (600 MHz, $CD_3OD$) ........ S36
$^{13}$C-NMR and $^{13}$C DEPT-135 spectra of compound **8** (151 MHz, $CD_3OD$) ........ S36

$^{1}$H-NMR spectrum of compound **9** (600 MHz, $CD_3OD$) ........ S37
$^{13}$C-NMR and $^{13}$C DEPT-135 spectra of compound **9** (151 MHz, $CD_3OD$) ........ S37
$^{1}$H-NMR spectrum of compound **10** (600 MHz, $CD_3OD$) ........ S38
$^{13}$C-NMR and $^{13}$C DEPT-135 spectra of compound **10** (151 MHz, $CD_3OD$) ........ S38
$^{1}$H-NMR spectrum of compound **11** (600 MHz, $CD_3OD$) ........ S39
$^{13}$C-NMR and $^{13}$C DEPT-135 spectra of compound **11** (151 MHz, $CD_3OD$) ........ S39
$^{1}$H-NMR spectrum of compound **12** (600 MHz, $CD_3OD$) ........ S40
$^{13}$C-NMR and $^{13}$C DEPT-135 spectra of compound **12** (151 MHz, $CD_3OD$) ........ S40
$^{1}$H-NMR spectrum of compound **13** (600 MHz, $CD_3OD$) ........ S41
$^{13}$C-NMR and $^{13}$C DEPT-135 spectra of compound **13** (151 MHz, $CD_3OD$) ........ S41
$^{1}$H-NMR spectrum of compound **14** (600 MHz, $CD_3OD$) ........ S42
$^{14}$C-NMR and $^{14}$C DEPT-135 spectra of compound **14** (151 MHz, $CD_3OD$) ........ S42
$^{1}$H-NMR spectrum of compound **15** (600 MHz, $CD_3OD$) ........ S43
$^{15}$C-NMR and $^{15}$C DEPT-135 spectra of compound **15** (151 MHz, $CD_3OD$) ........ S43
$^{1}$H-NMR spectrum of compound **16** (600 MHz, $CD_3OD$) ........ S44
$^{16}$C-NMR and $^{16}$C DEPT-135 spectra of compound **16** (151 MHz, $CD_3OD$) ........ S44
$^{1}$H-NMR spectrum of compound **17** (600 MHz, $CD_3OD$) ........ S45
$^{13}$C-NMR and $^{13}$C DEPT-135 spectra of compound **17** (151 MHz, $CD_3OD$) ........ S45
$^{1}$H-NMR spectrum of compound **18** (600 MHz, $CD_3OD$) ........ S46
$^{13}$C-NMR and $^{13}$C DEPT-135 spectra of compound **18** (151 MHz, $CD_3OD$) ........ S46
$^{1}$H-NMR spectrum of compound **19** (600 MHz, $CD_3OD$) ........ S47
$^{13}$C-NMR and $^{13}$C DEPT-135 spectra of compound **19** (151 MHz, $CD_3OD$) ........ S47
$^{1}$H-NMR spectrum of compound **20** (600 MHz, $CD_3OD$) ........ S48
$^{13}$C-NMR and $^{13}$C DEPT-135 spectra of compound **20** (151 MHz, $CD_3OD$) ........ S48
$^{1}$H-NMR spectrum of compound **21** (600 MHz, $CD_3OD$) ........ S49
$^{13}$C-NMR and $^{13}$C DEPT-135 spectra of compound **21** (151 MHz, $CD_3OD$) ........ S49
$^{1}$H-NMR spectrum of compound **22** (600 MHz, $CD_3OD$) ........ S50
$^{1}$H-NMR spectrum of compound **23** (600 MHz, $CD_3OD$) ........ S51
$^{13}$C-NMR and $^{13}$C DEPT-135 spectra of compound **23** (151 MHz, $CD_3OD$) ........ S51
$^{1}$H-NMR spectrum of compound **24** (600 MHz, $CD_3OD$) ........ S52
$^{13}$C-NMR and $^{13}$C DEPT-135 spectra of compound **24** (151 MHz, $CD_3OD$) ........ S52
$^{1}$H-NMR spectrum of compound **25** (600 MHz, $CD_3OD$) ........ S53

$^{13}$C-NMR and $^{13}$C DEPT-135 spectra of compound **25** (151 MHz, $CD_3OD$) ....................... S53

$^{1}$H-NMR spectrum of compound **26** (600 MHz, $CD_3OD$) .................................................... S54

$^{13}$C-NMR and $^{13}$C DEPT-135 spectra of compound **26** (151 MHz, $CD_3OD$) ....................... S54

$^{1}$H and $^{13}$C NMR data of compounds **8-26** .......................................................................... S55

Effects of compounds **4, 5, 9, 10, 14, 15, 16, 18, 19, 21, 22, 23, 24** and **26** on glutamate-induced oxidative injury of SH-SY5Y cells. .......................................................................... S59

$^{1}$H-NMR spectrum of compound **1** (600 MHz, $CD_3OD$)

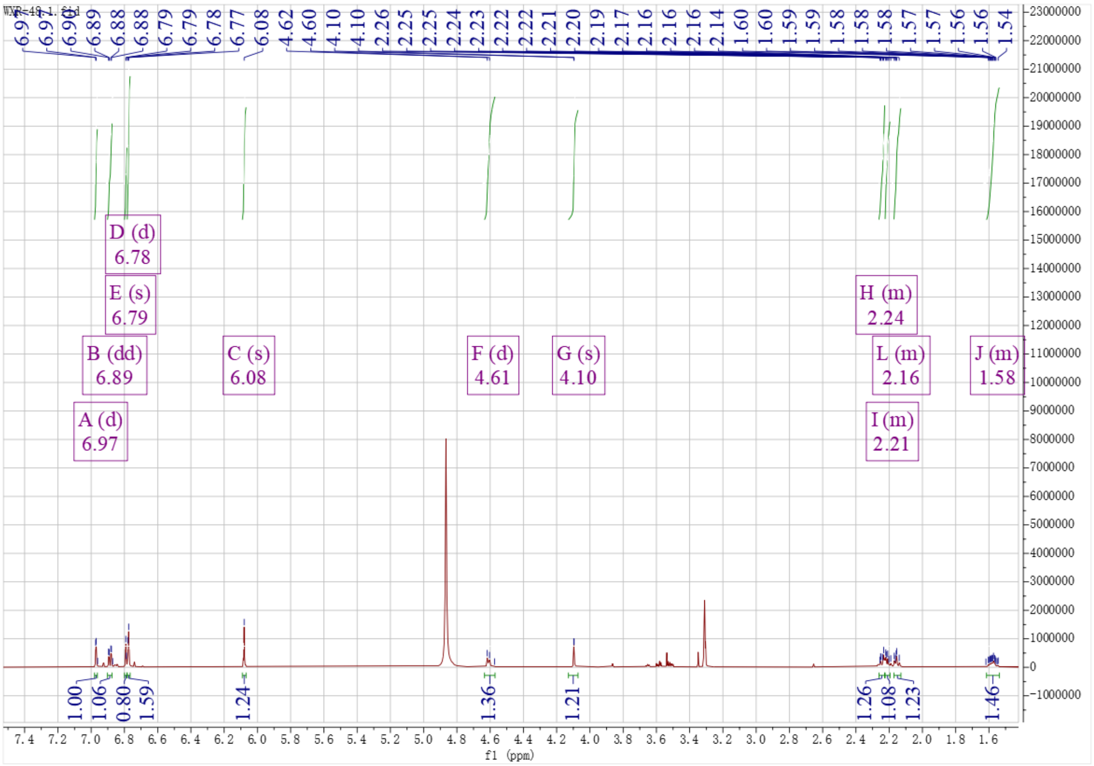

$^{13}$C-NMR spectrum of compound **1** (151 MHz, $CD_3OD$)

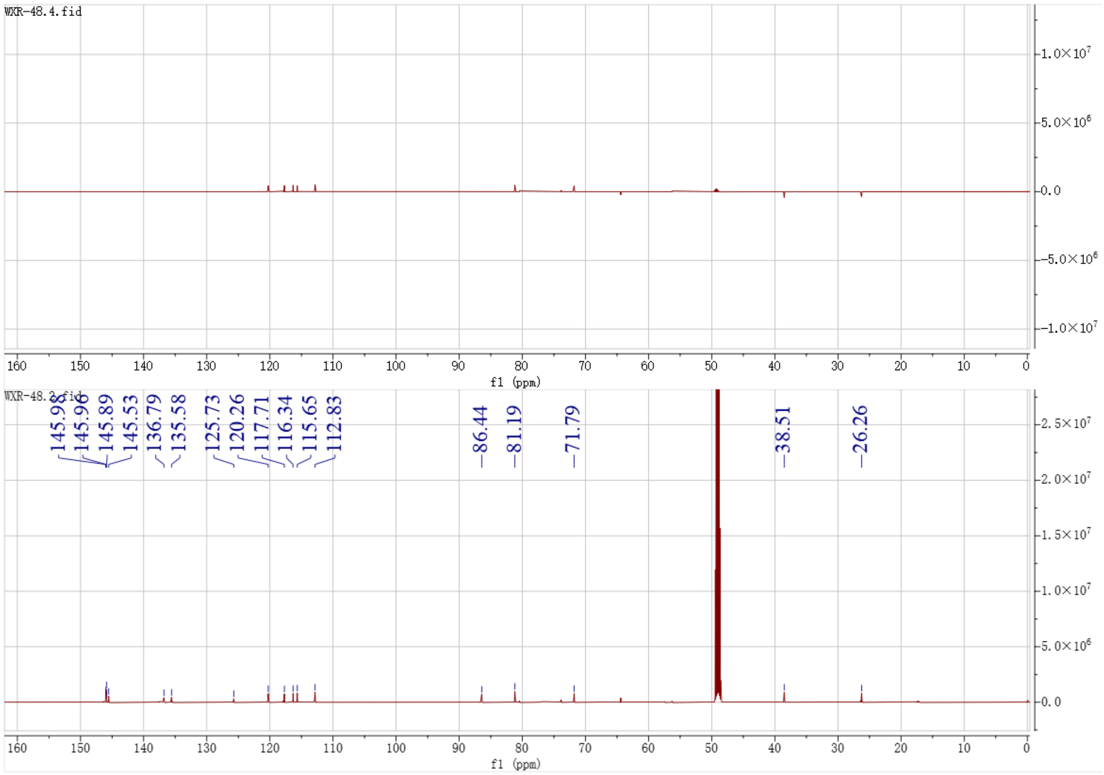

$^{1}$H-$^{1}$H COSY spectrum of compound **1**

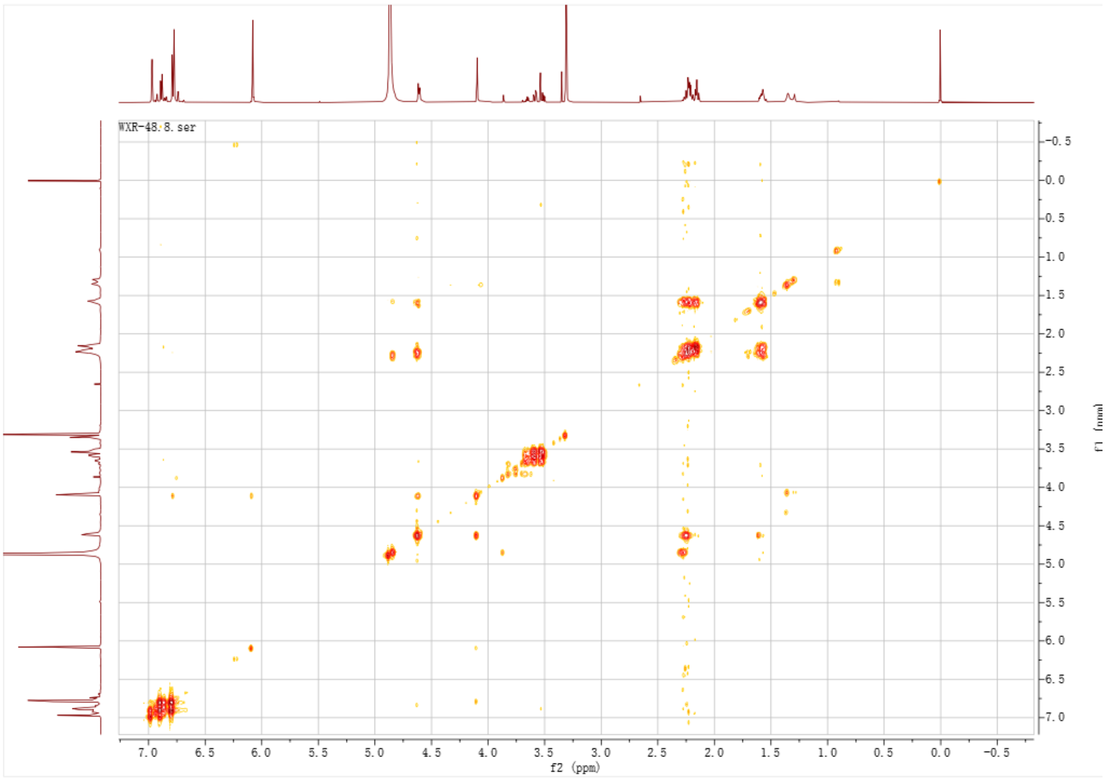


HSQC spectrum of compound **1**

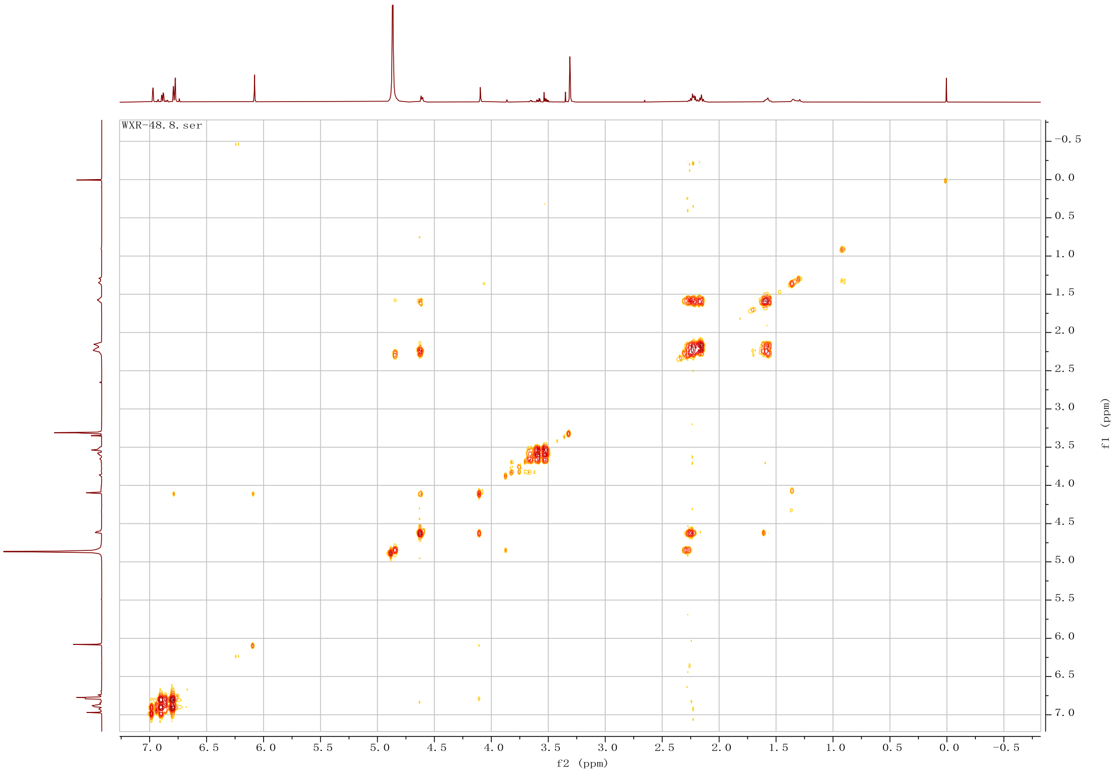

HMBC spectrum of compound **1**

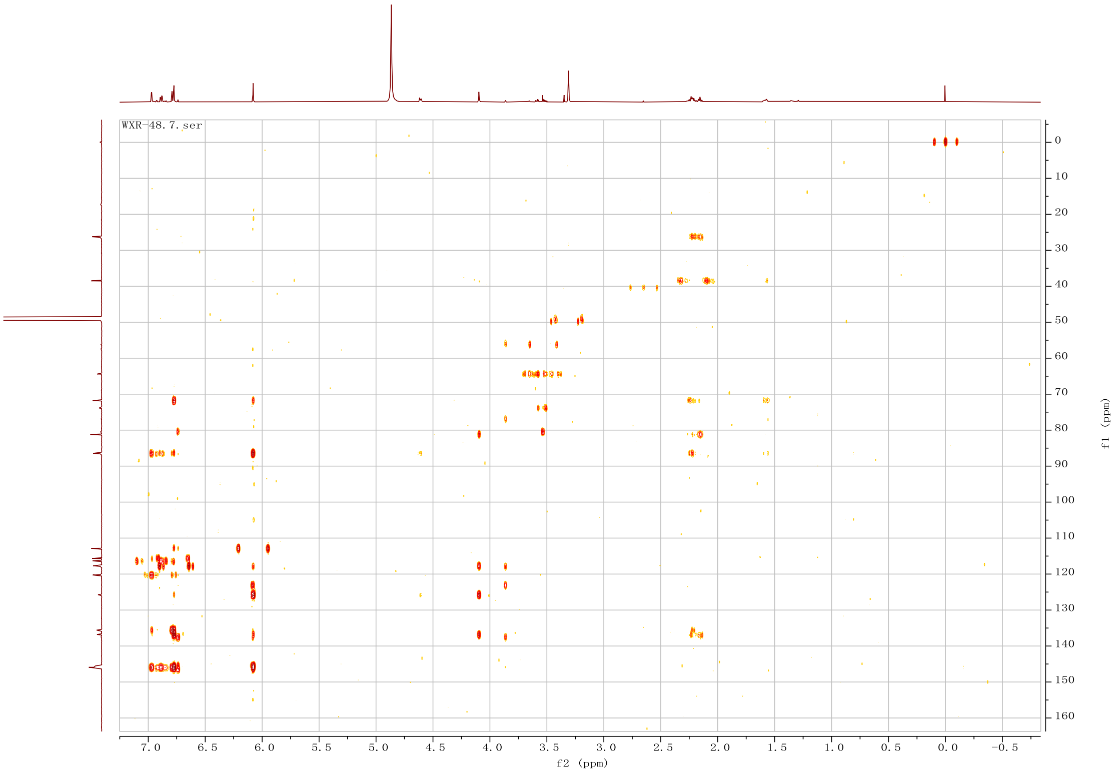


NOESY spectrum of compound **1**

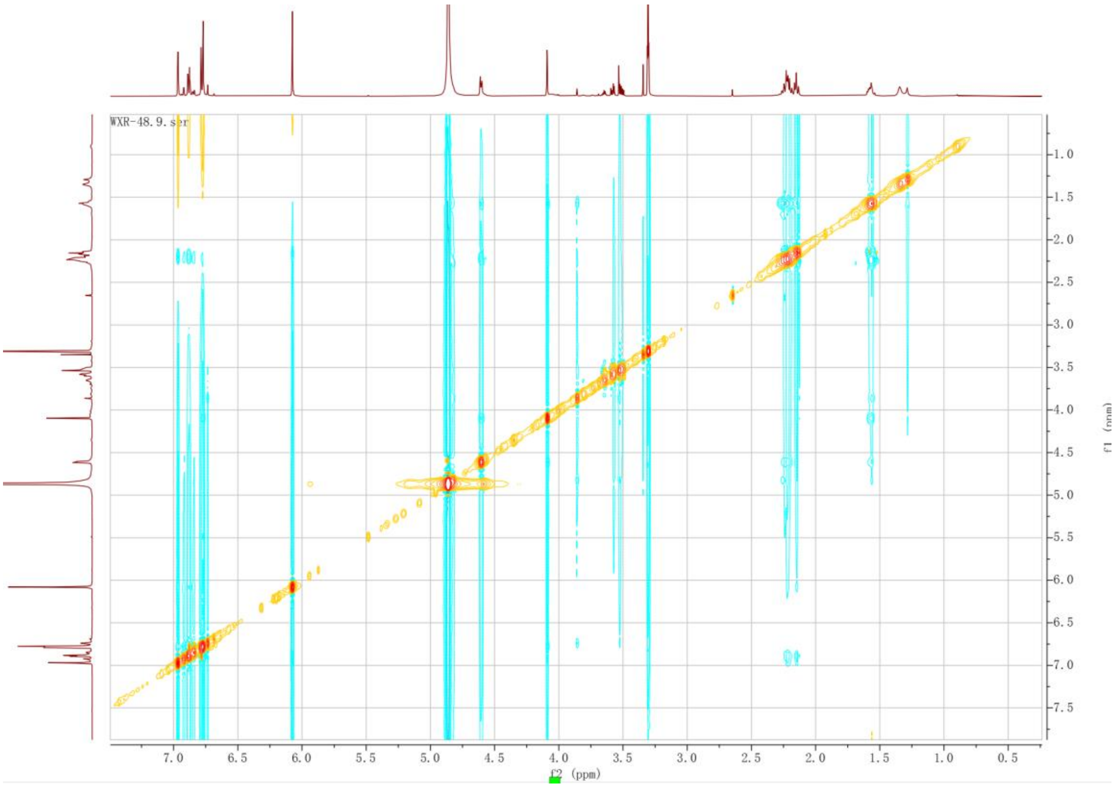

HRESIMS plot of compound **1**

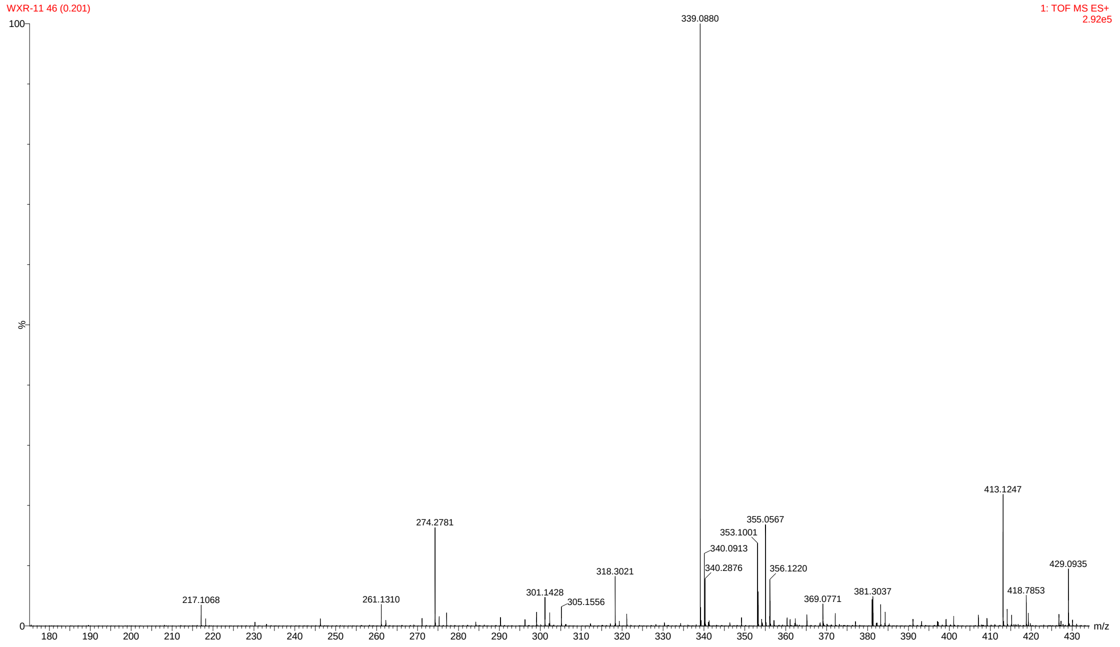

IR spectrum of compound **1**

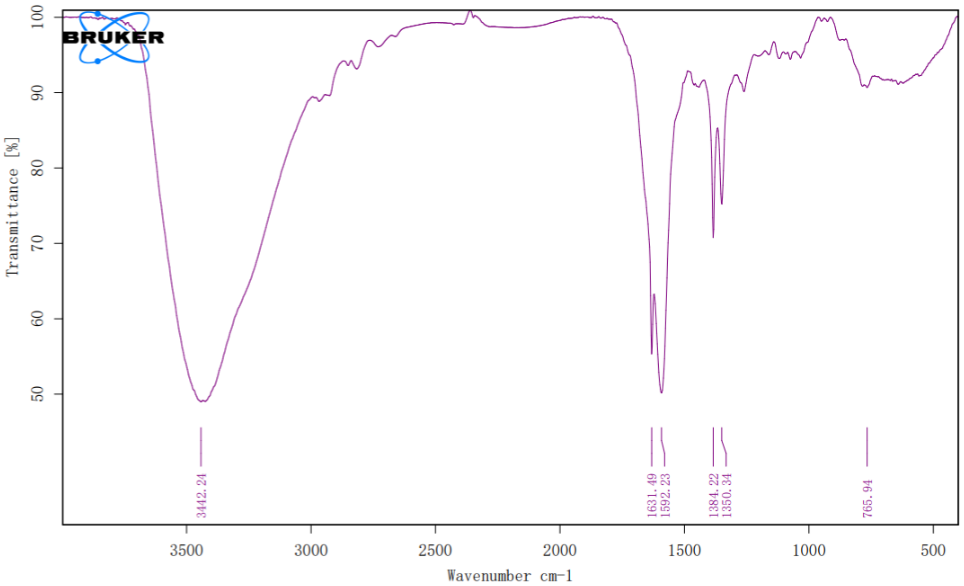


UV spectrum of compound **1**

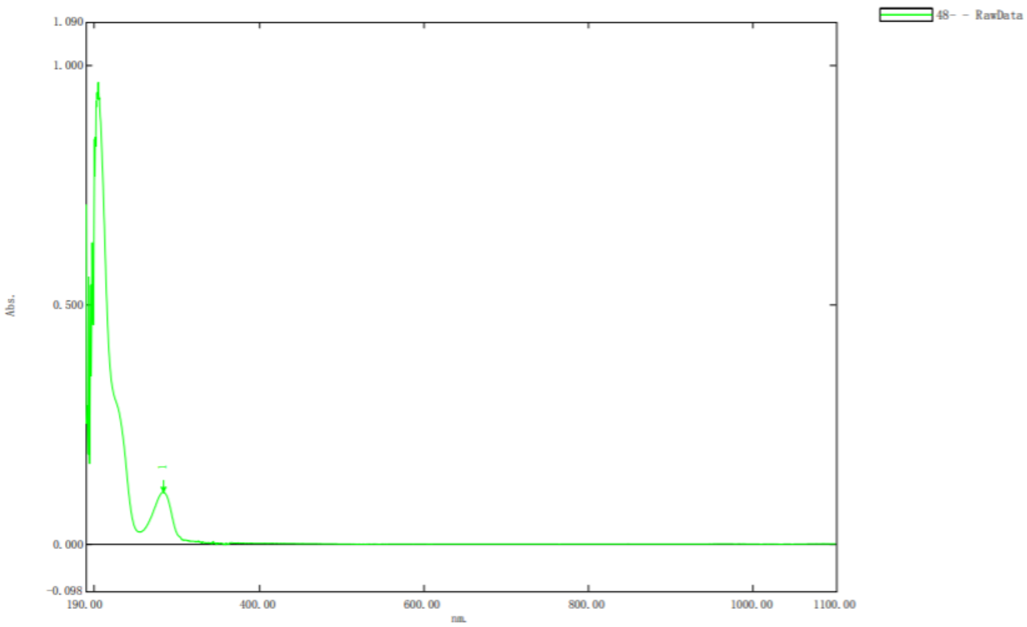

$^{1}$H-NMR spectrum of compound **2** (600 MHz, $CD_3OD$)

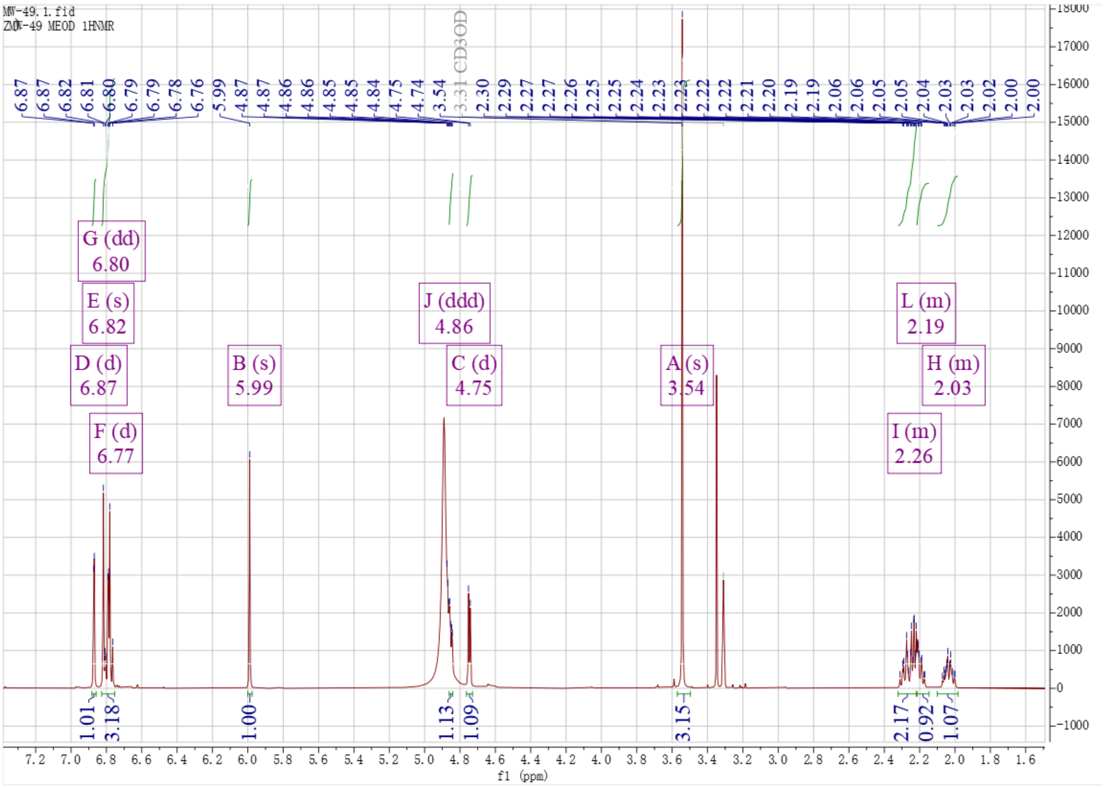

$^{13}$C-NMR spectrum of compound **2** (151 MHz, $CD_3OD$)

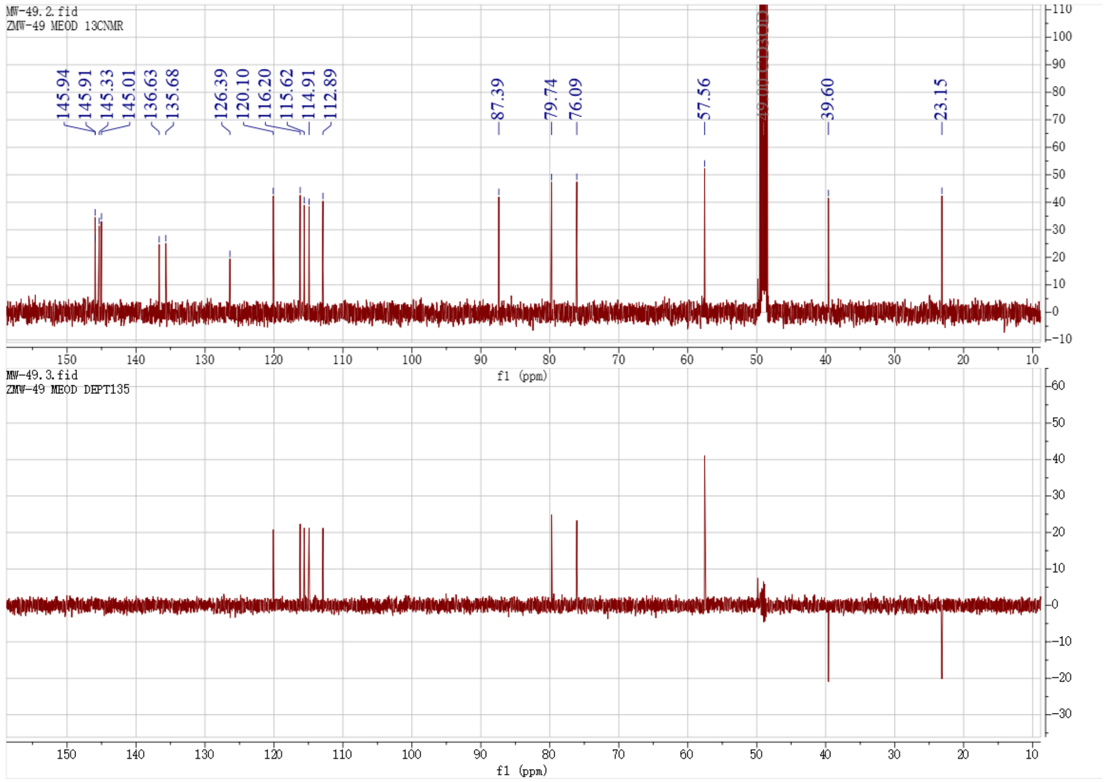

$^{1}$H-$^{1}$H COSY spectrum of compound **2**

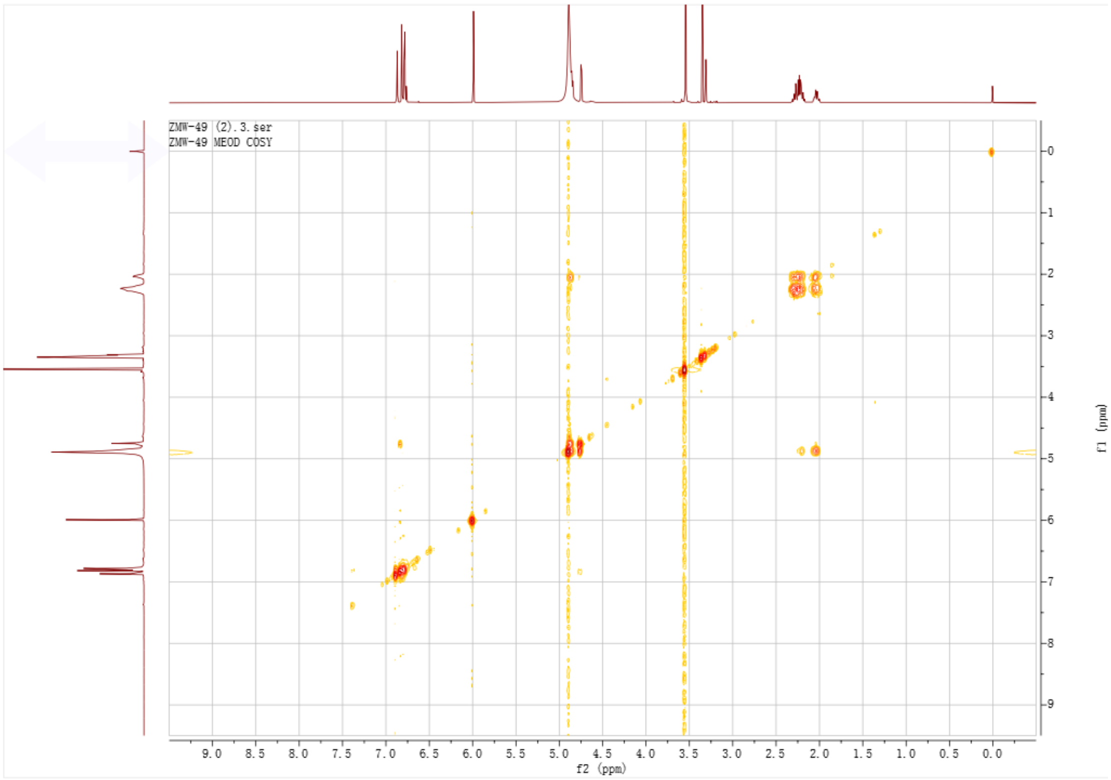


HSQC spectrum of compound **2**

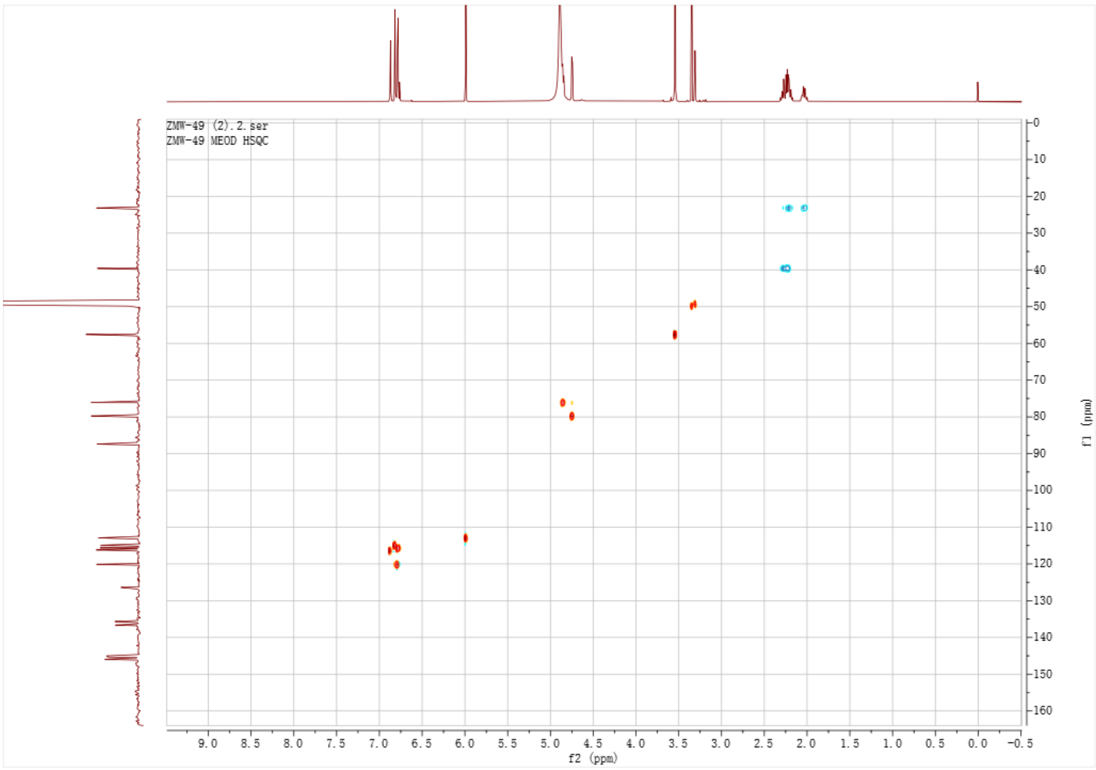

HMBC spectrum of compound **2**

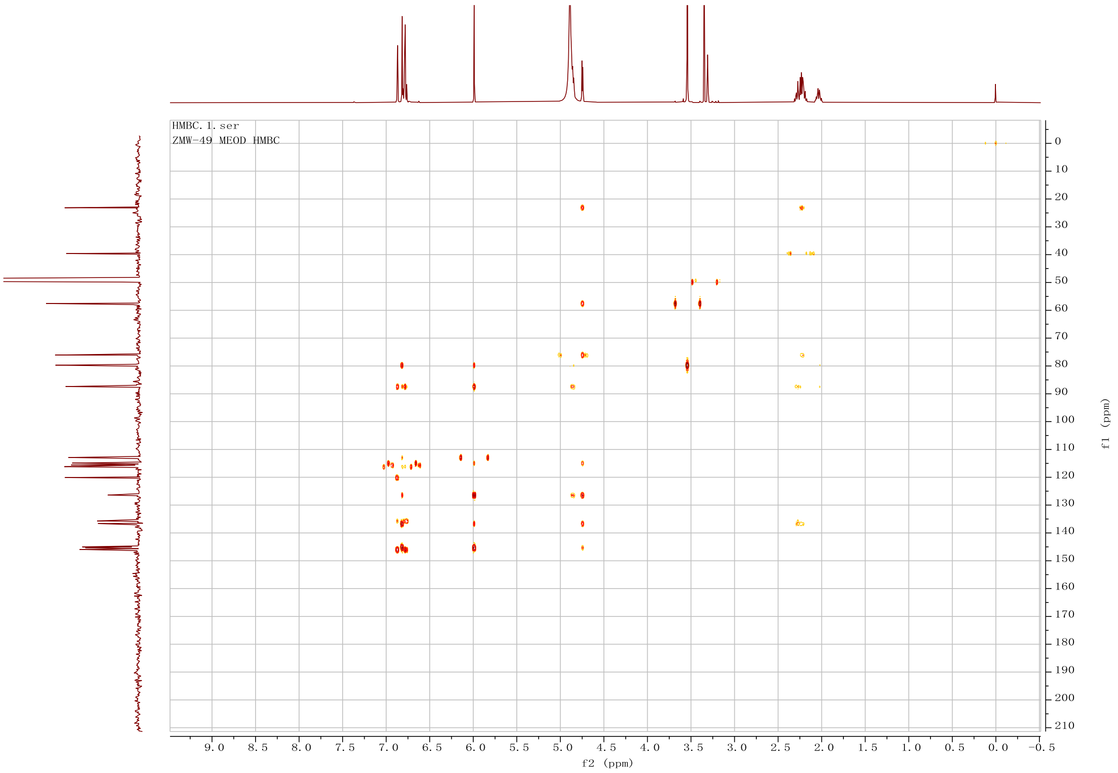


NOESY spectrum of compound **2**

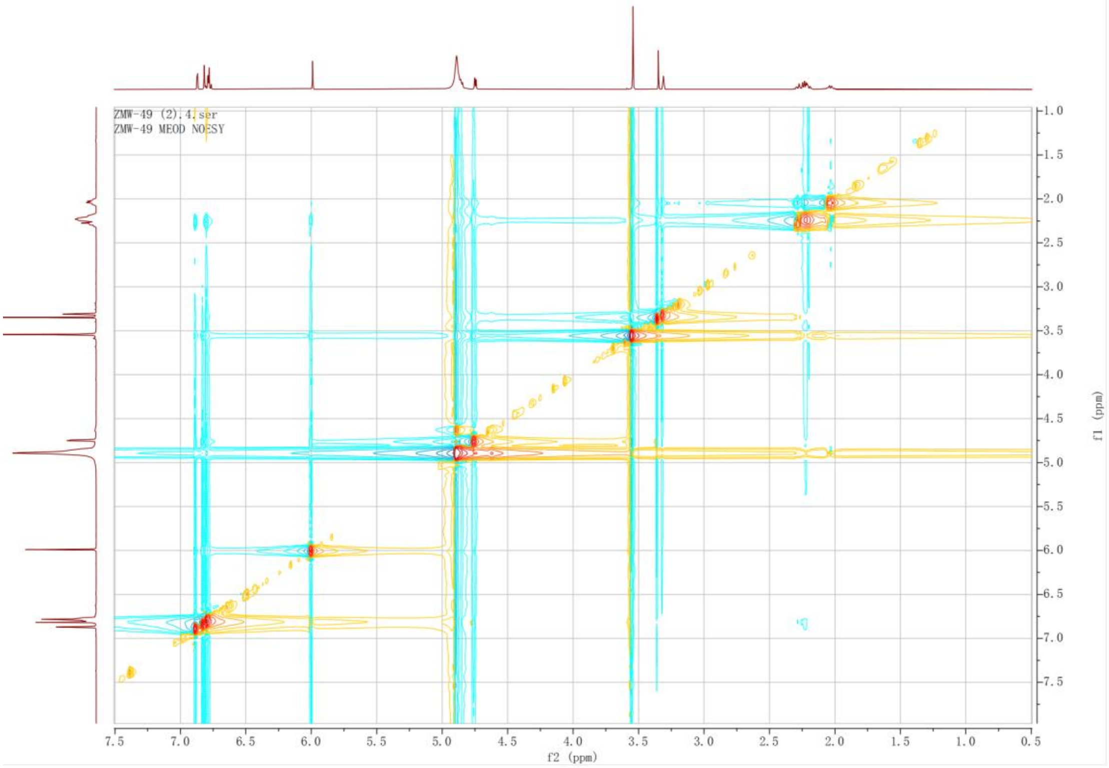

# HRESIMS plot of compound **2**

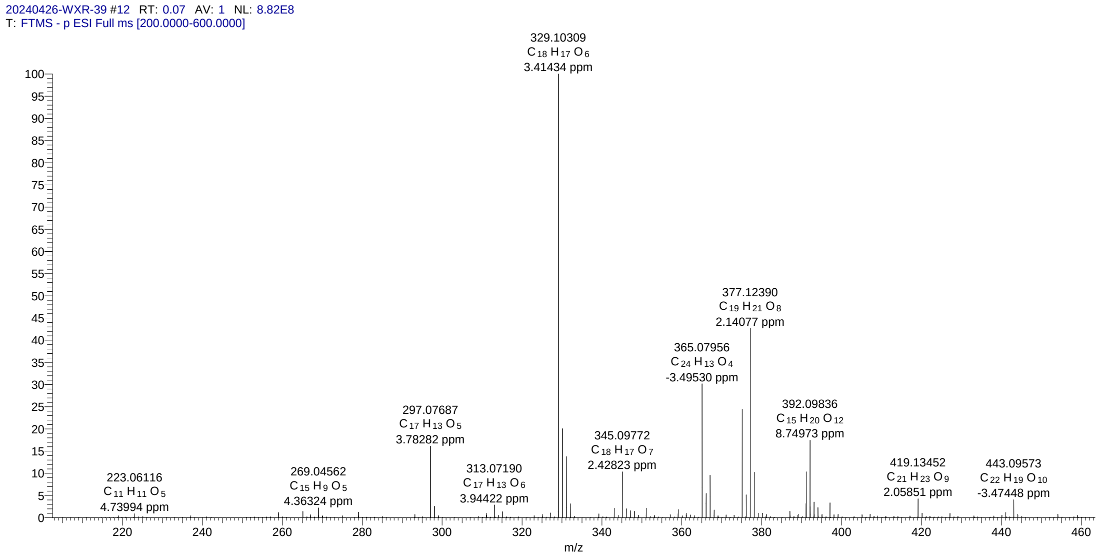

IR spectrum of compound **2**

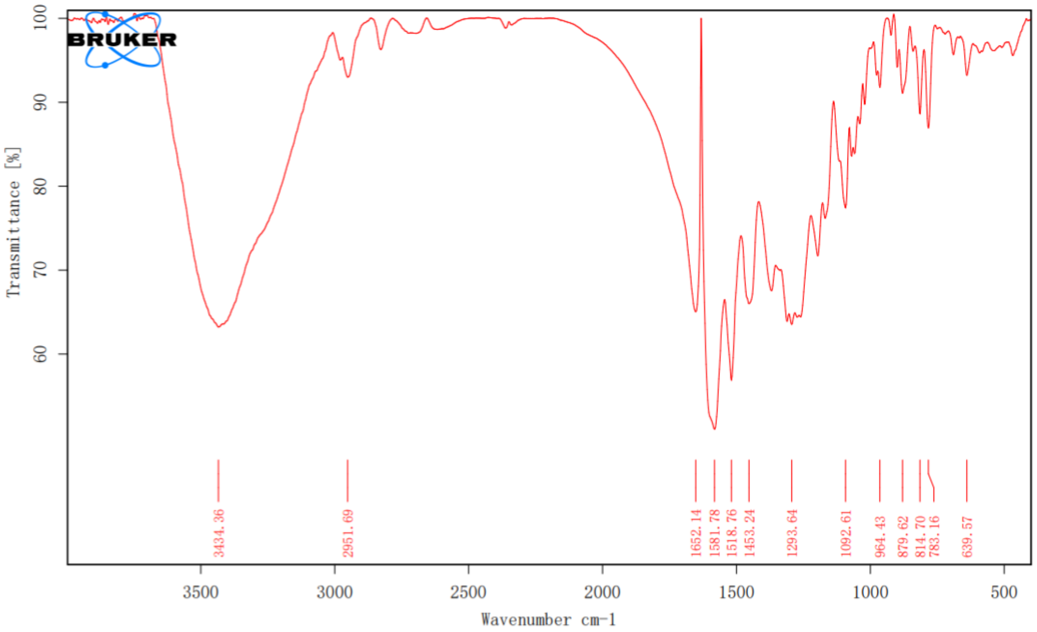


UV spectrum of compound **2**

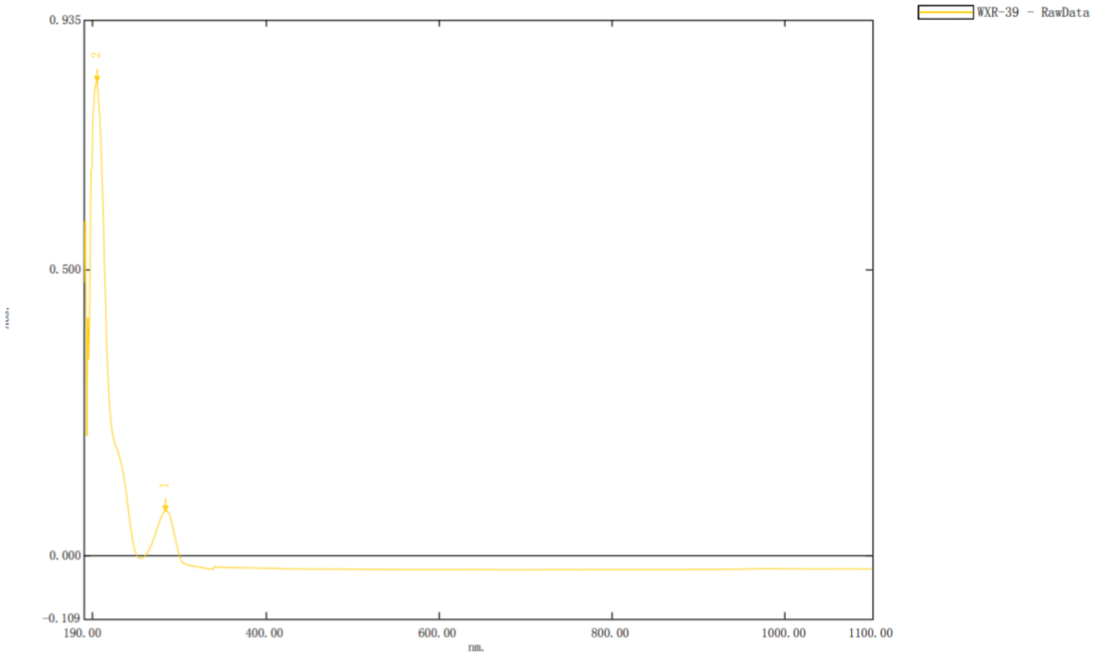

$^{1}$H-NMR spectrum of compound **3** (600 MHz, $CD_3OD$)

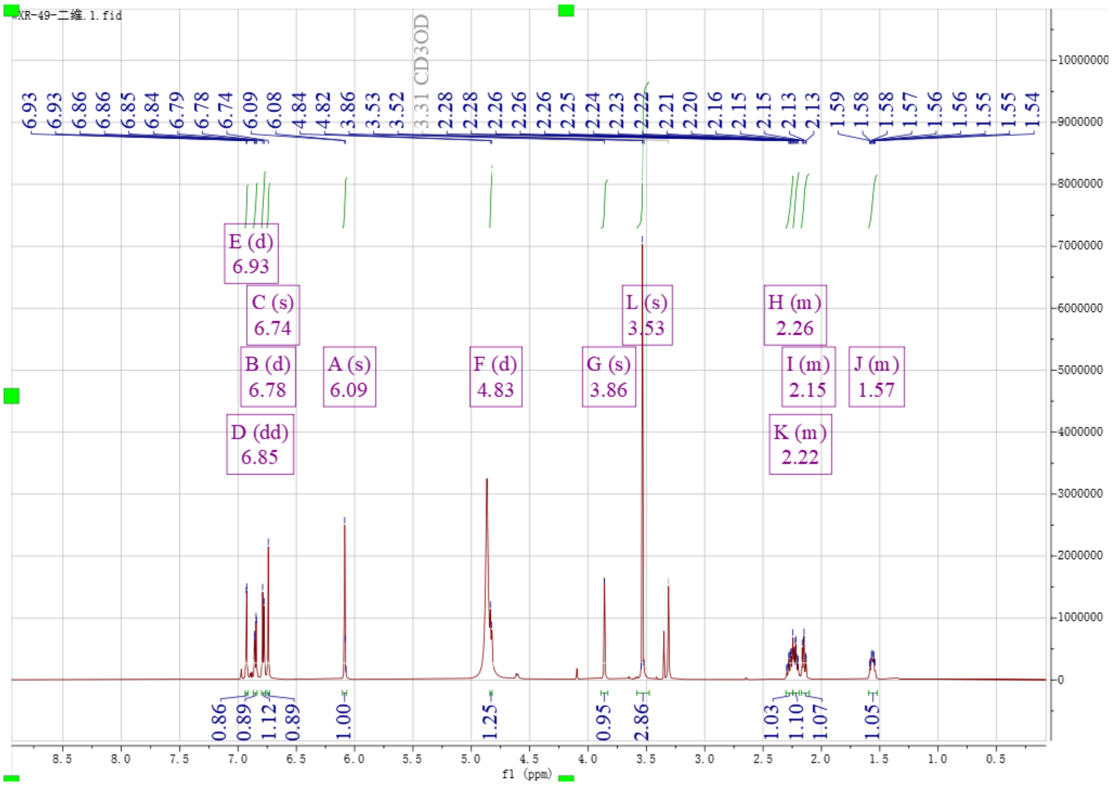

$^{13}$C-NMR spectrum of compound **3** (151 MHz, $CD_3OD$)

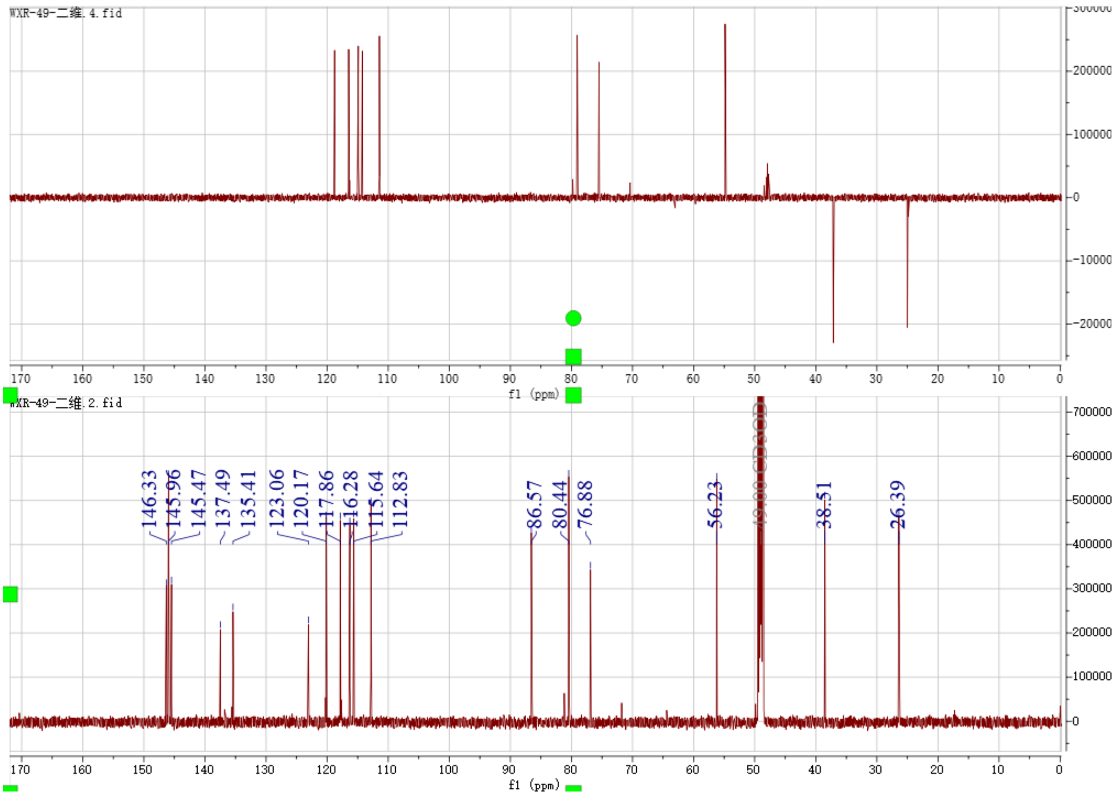

$^1$H-$^1$H COSY spectrum of compound **3**

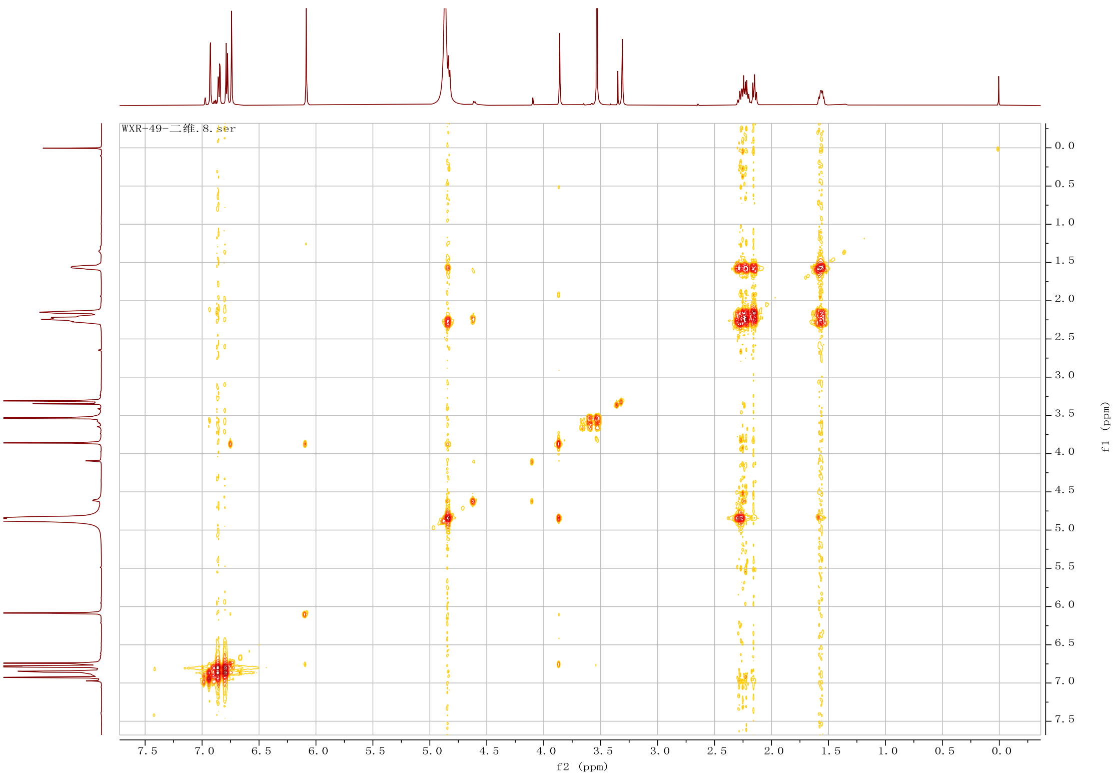


HSQC spectrum of compound **3**

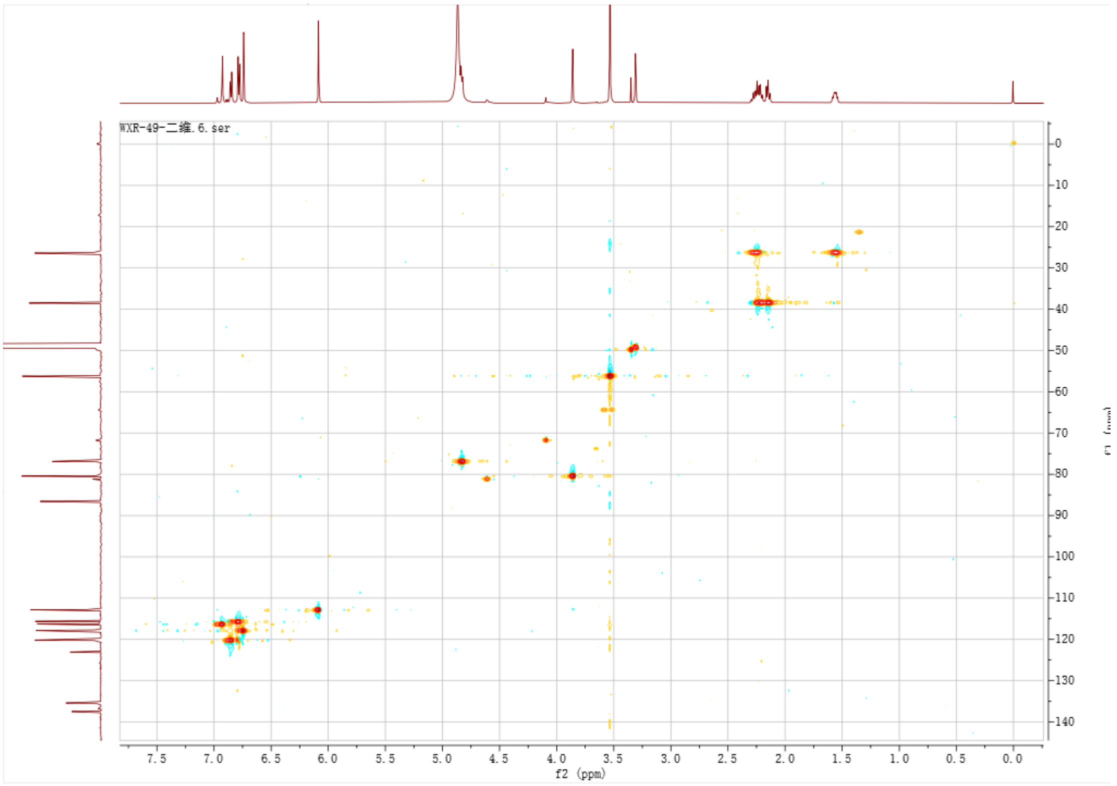

HMBC spectrum of compound **3**

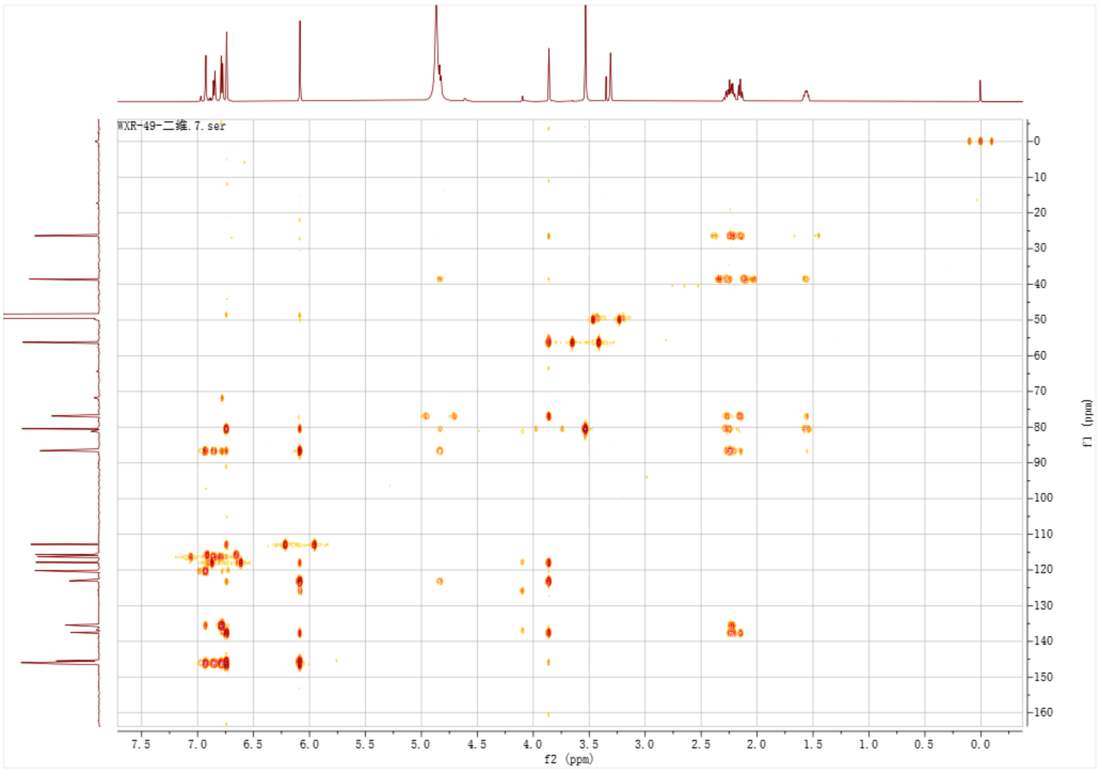


NOESY spectrum of compound **3**

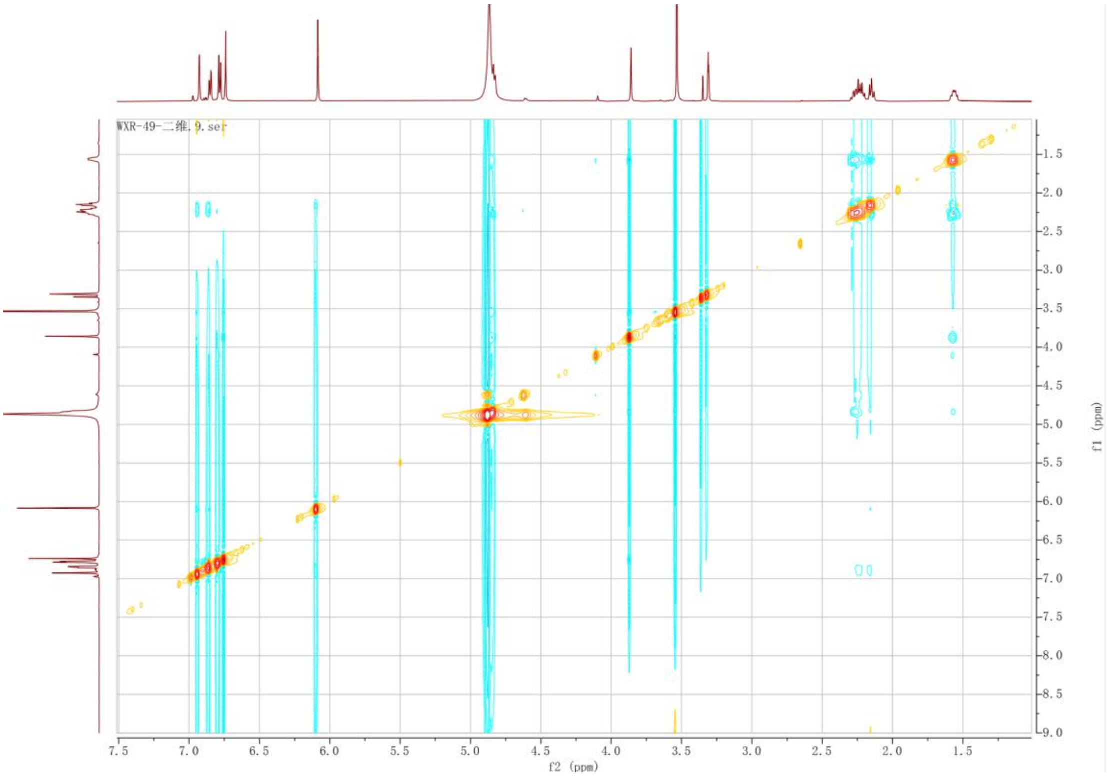

# HRESIMS plot of compound **3**

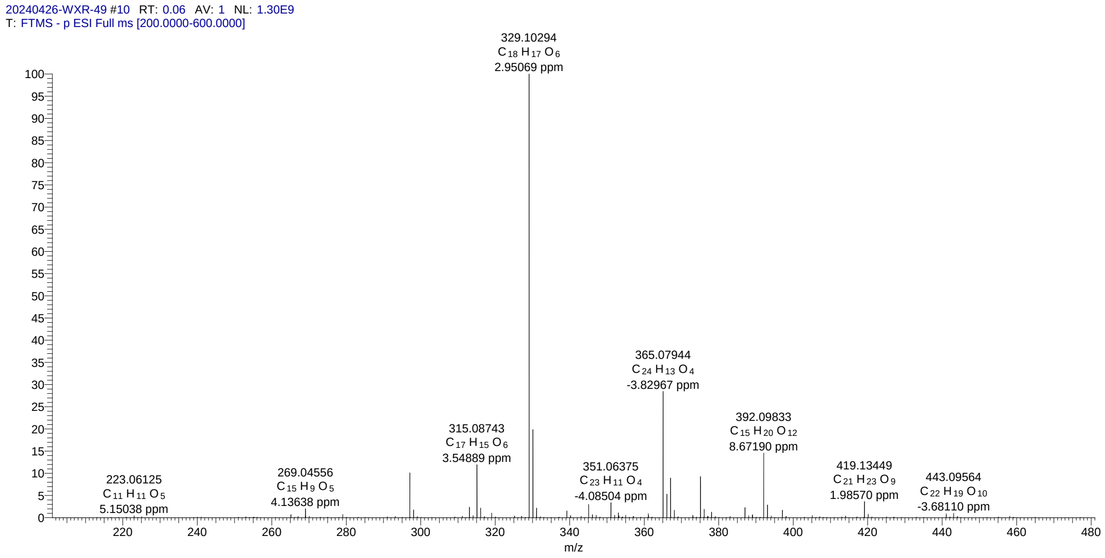

IR spectrum of compound **3**

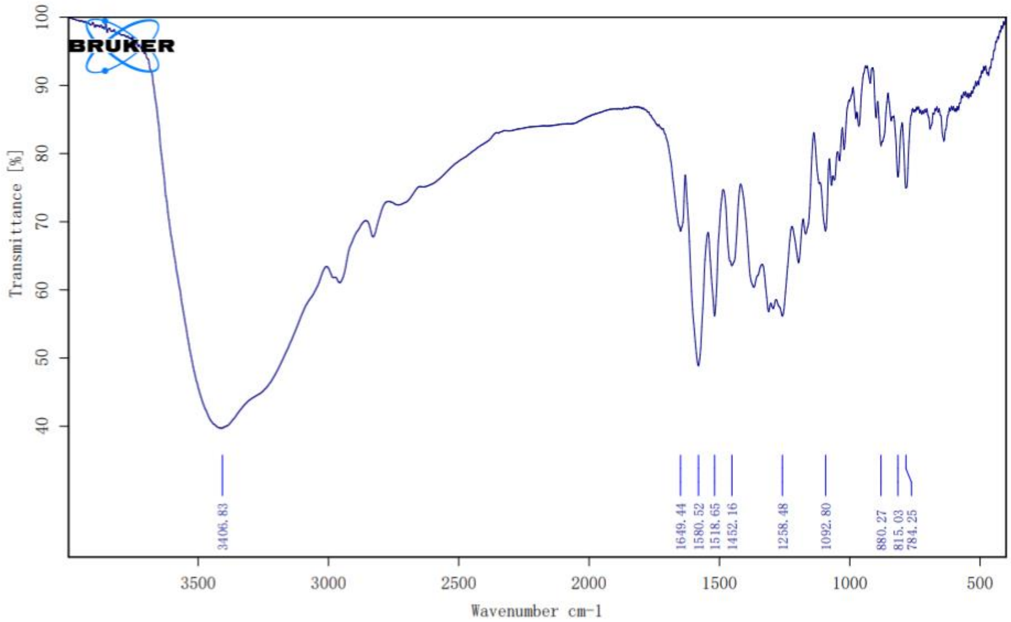


UV spectrum of compound **3**

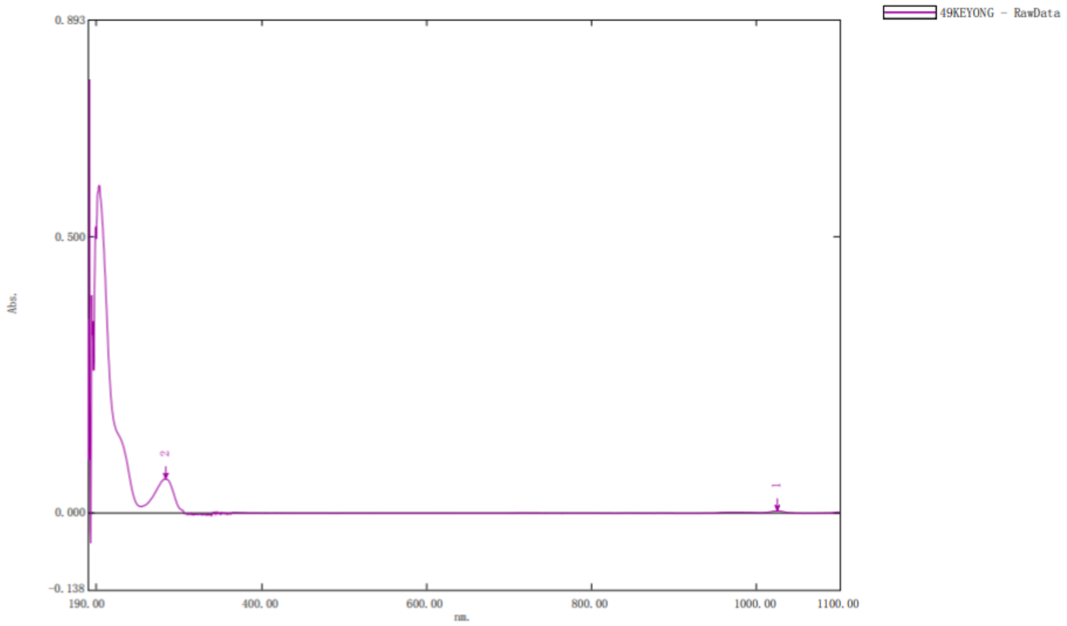

$^{1}$H-NMR spectrum of compound **4** (600 MHz, DMSO-$d_6$)

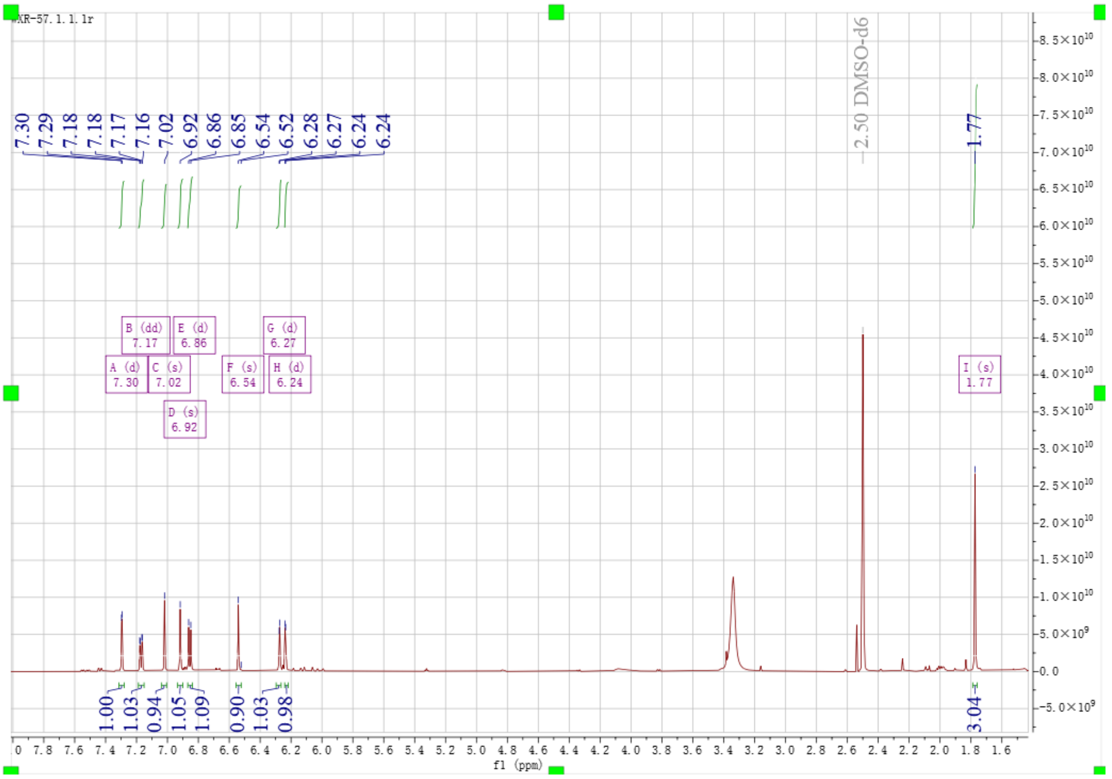

$^{13}$C-NMR spectrum of compound **4** (151 MHz, DMSO-$d_6$)

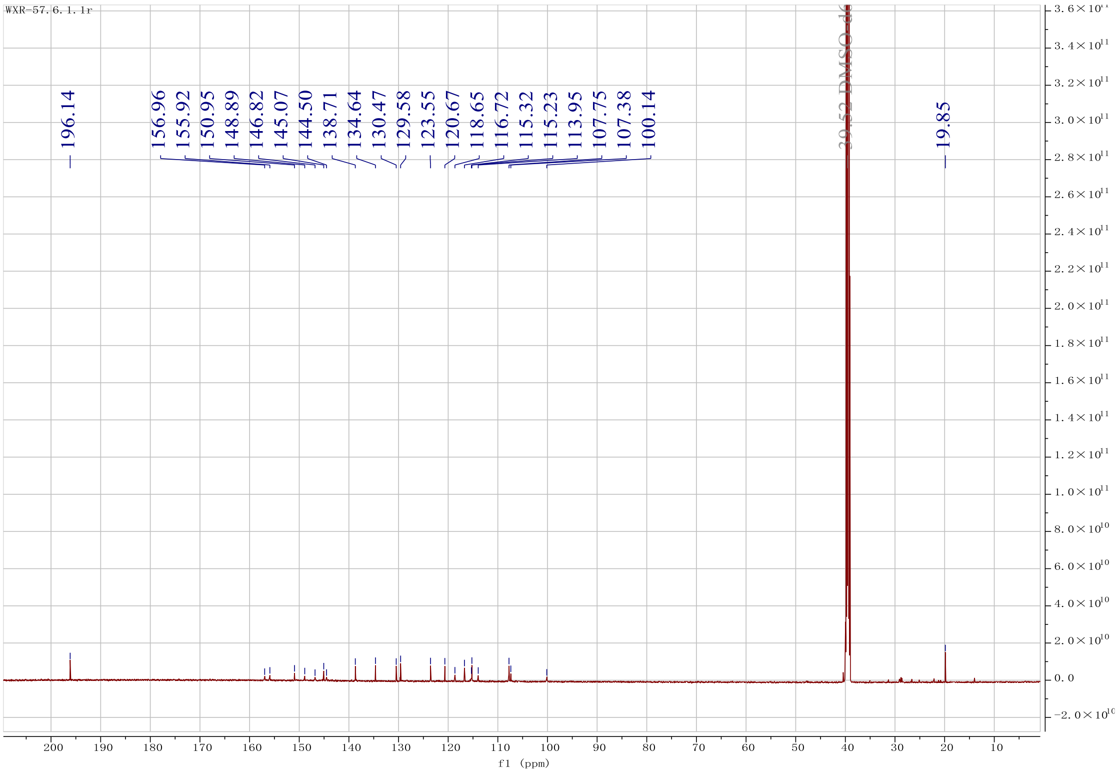

$^{1}$H-$^{1}$H COSY spectrum of compound **4**

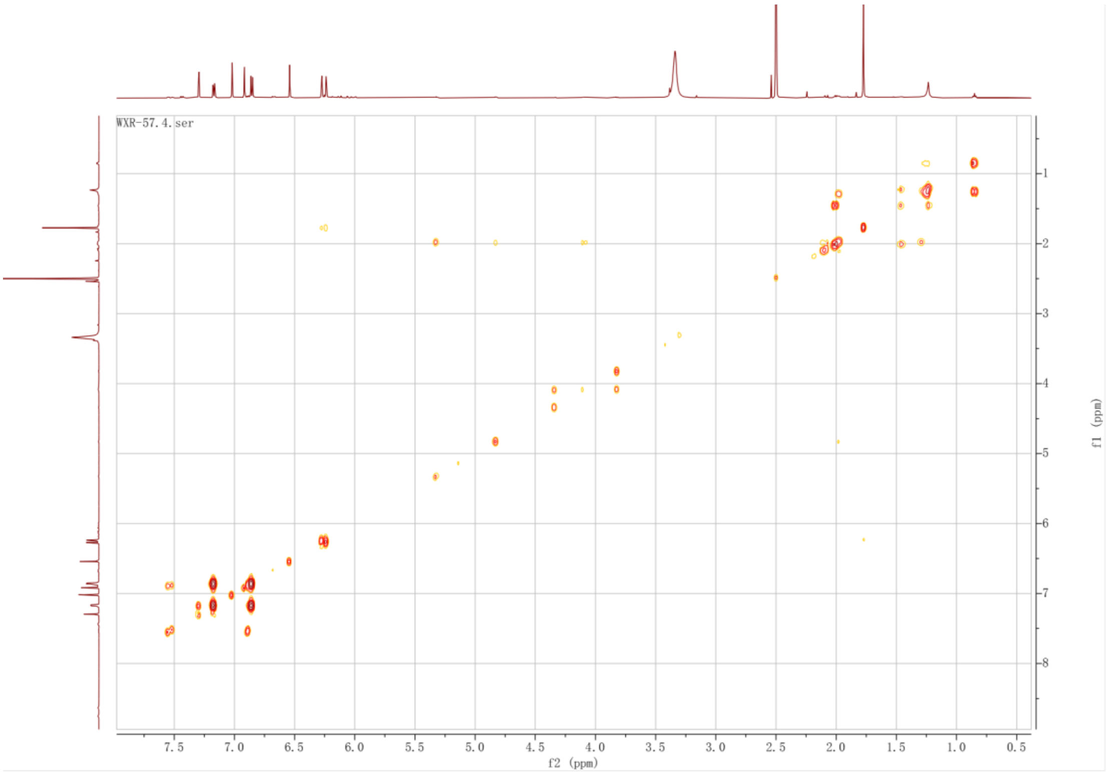


HSQC spectrum of compound **4**

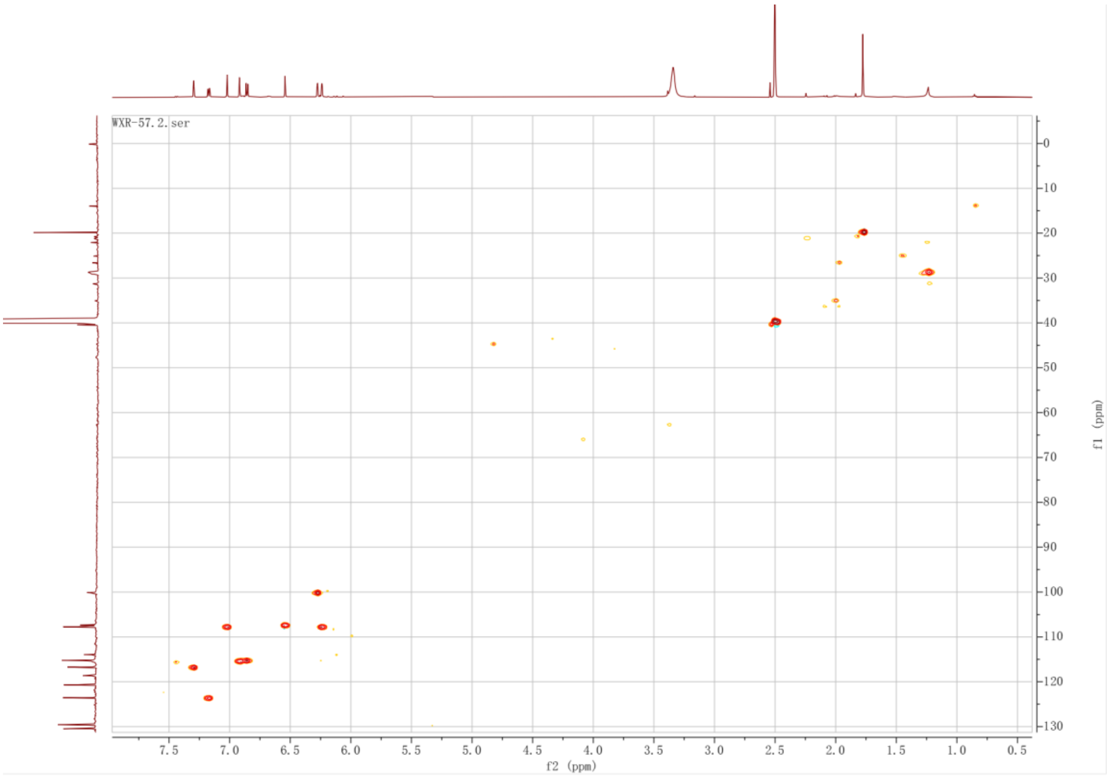

HMBC spectrum of compound 4

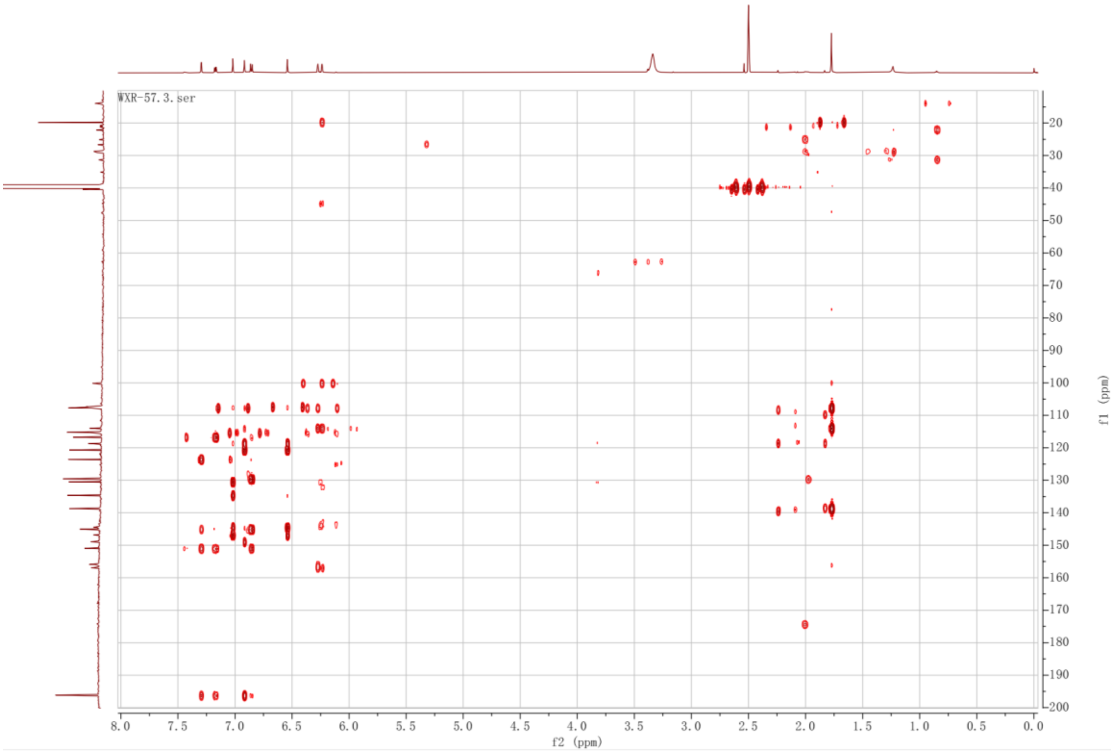


NOESY spectrum of compound **4**

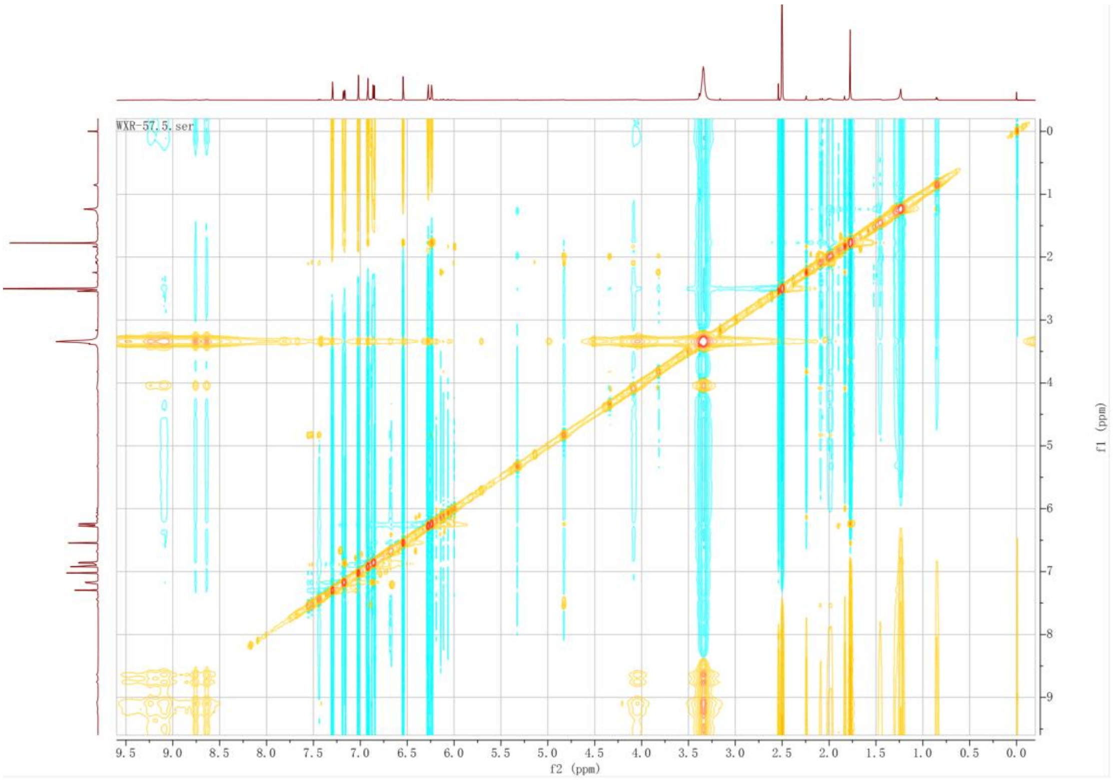

HRESIMS plot of compound **4**

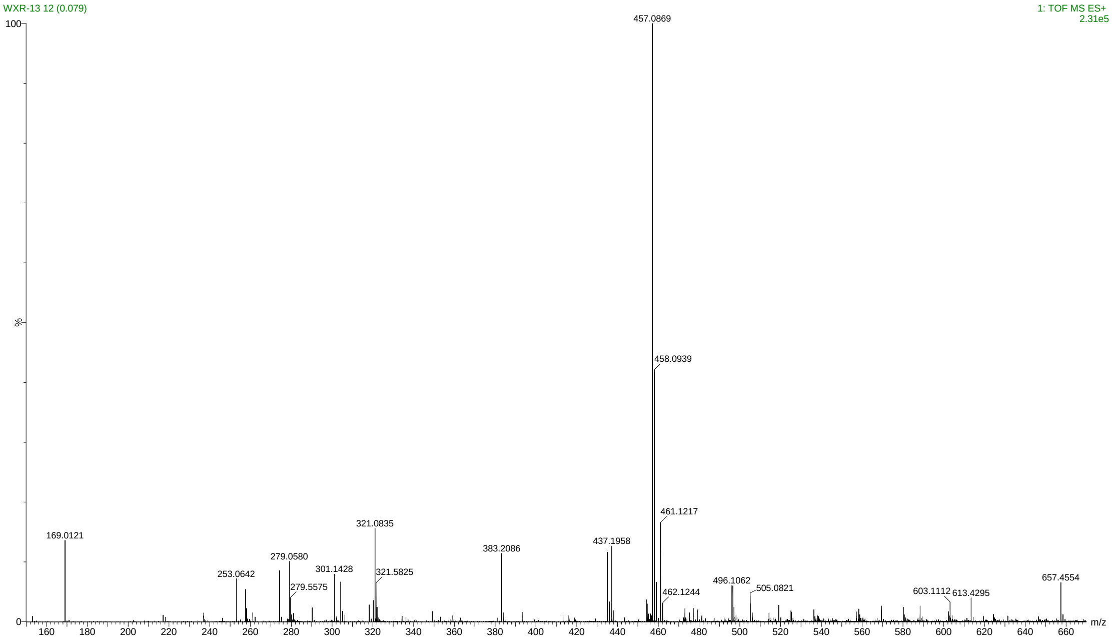

IR spectrum of compound **4**

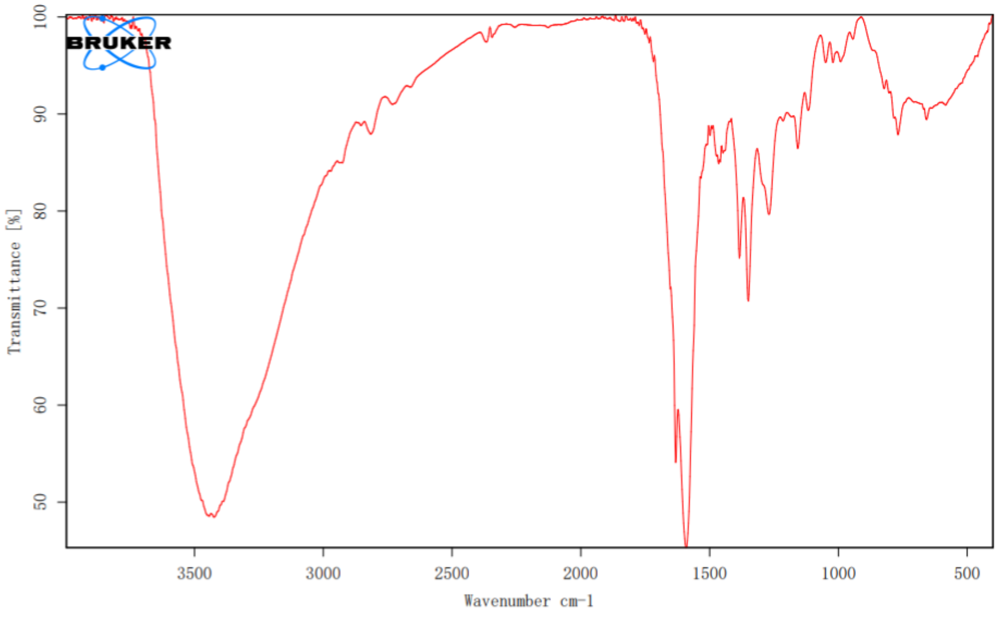


UV spectrum of compound **4**

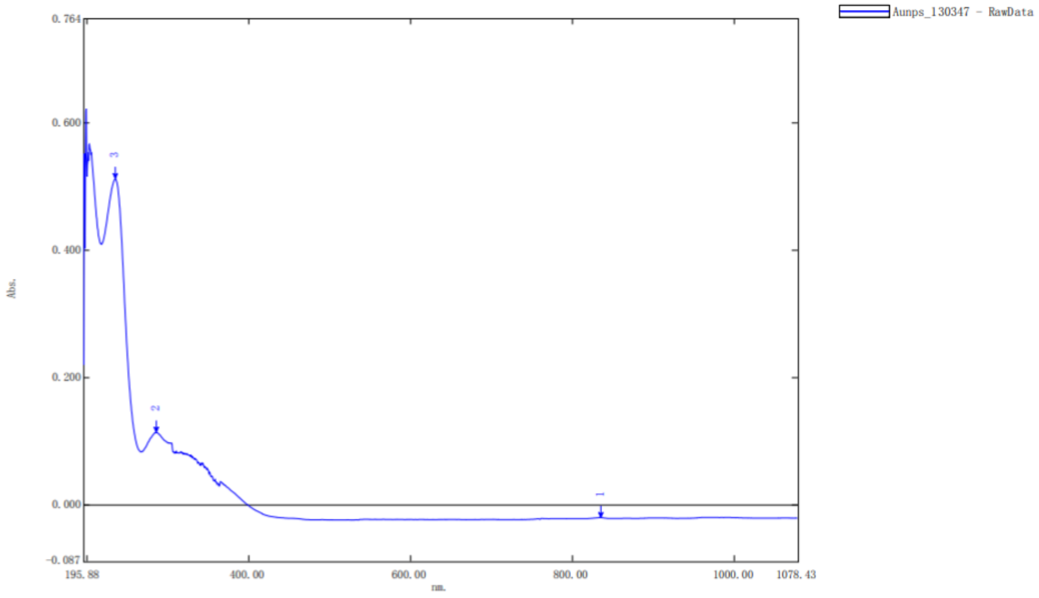

$^{1}$H-NMR spectrum of compound **5** (600 MHz, DMSO-$d_6$)

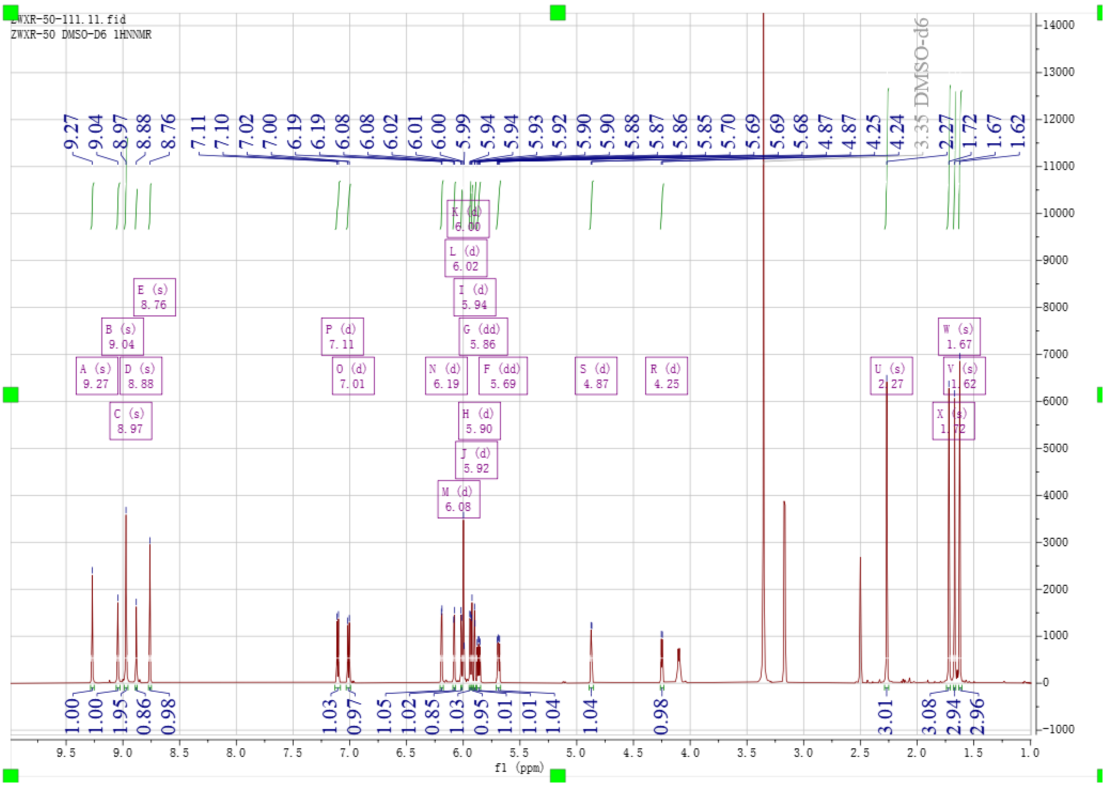


$^{13}$C-NMR spectrum of compound **5** (151 MHz, DMSO-$d_6$)

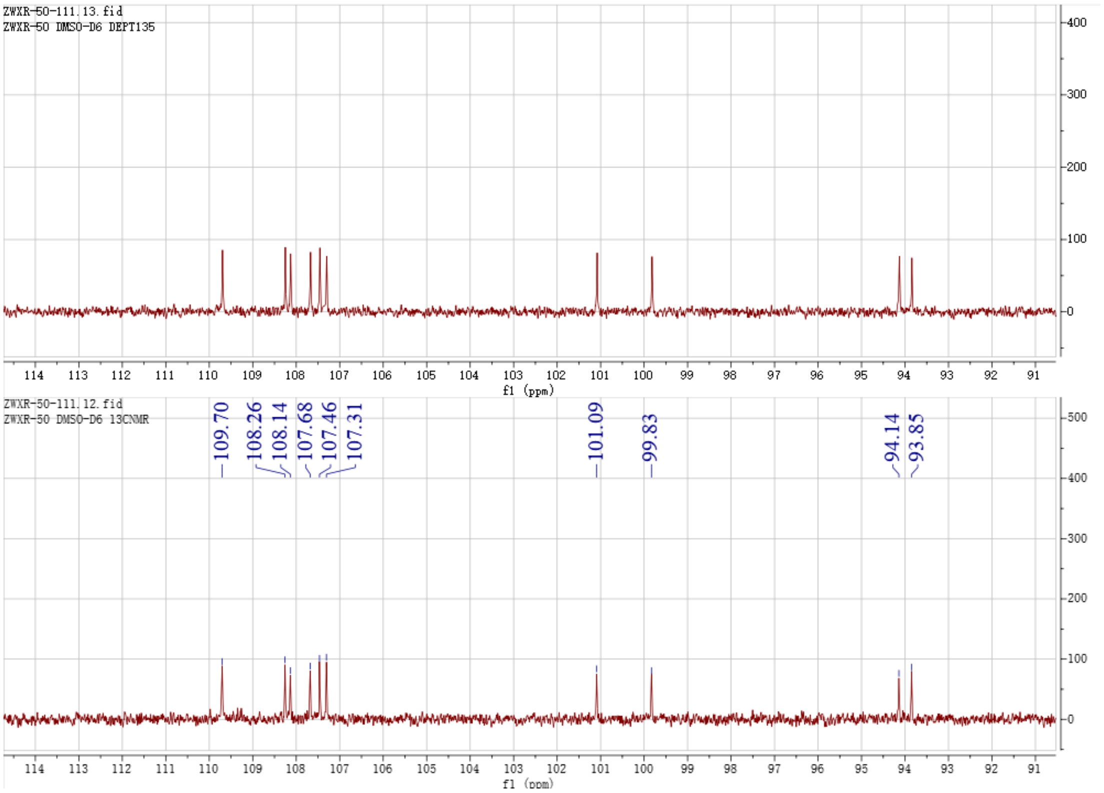

$^1H$-$^1H$ COSY spectrum of compound **5**

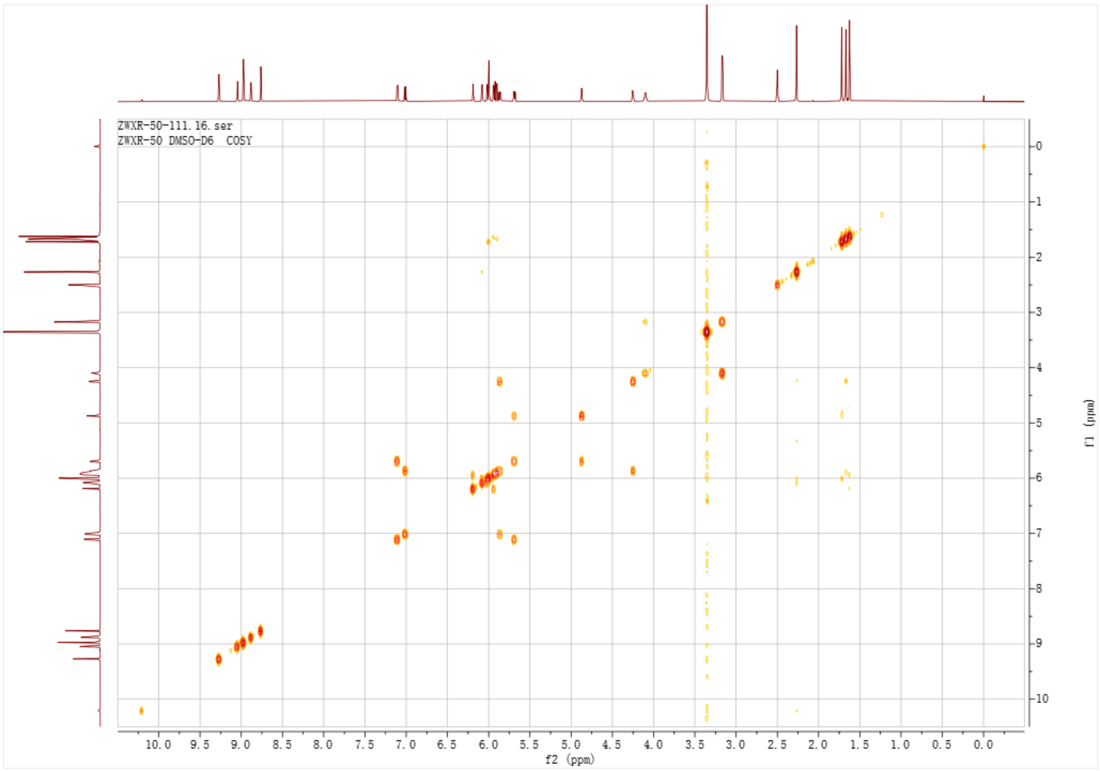


HSQC spectrum of compound **5**

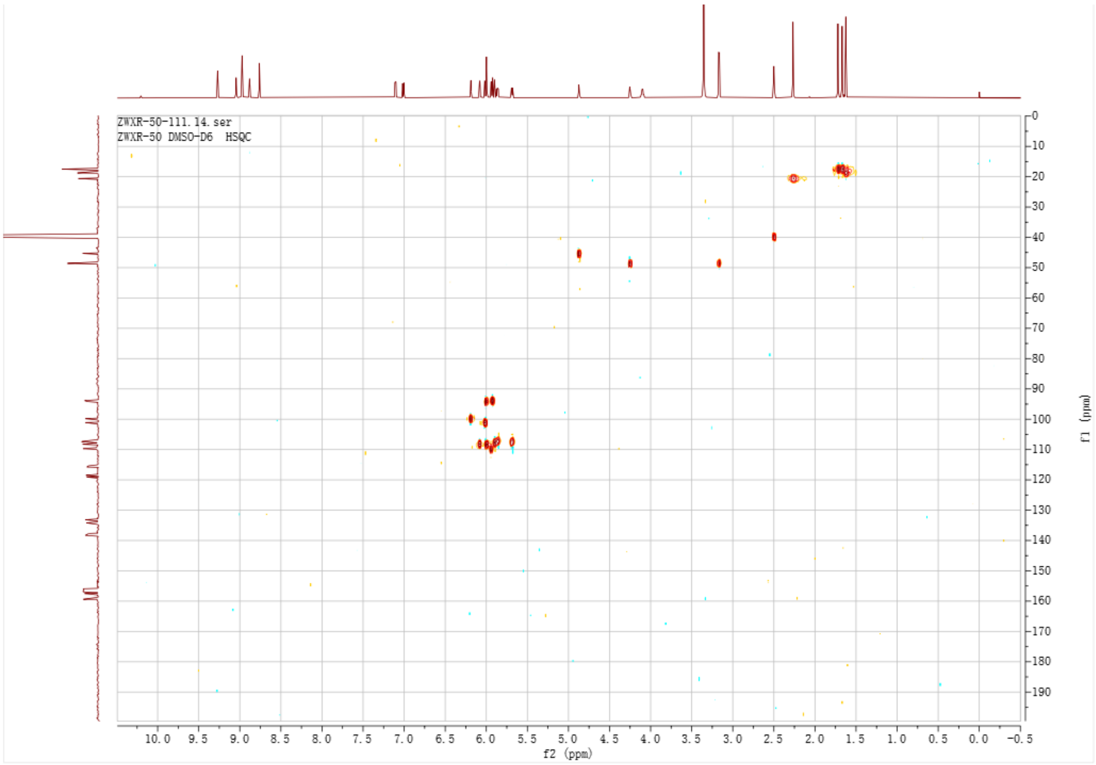

HMBC spectrum of compound **5**

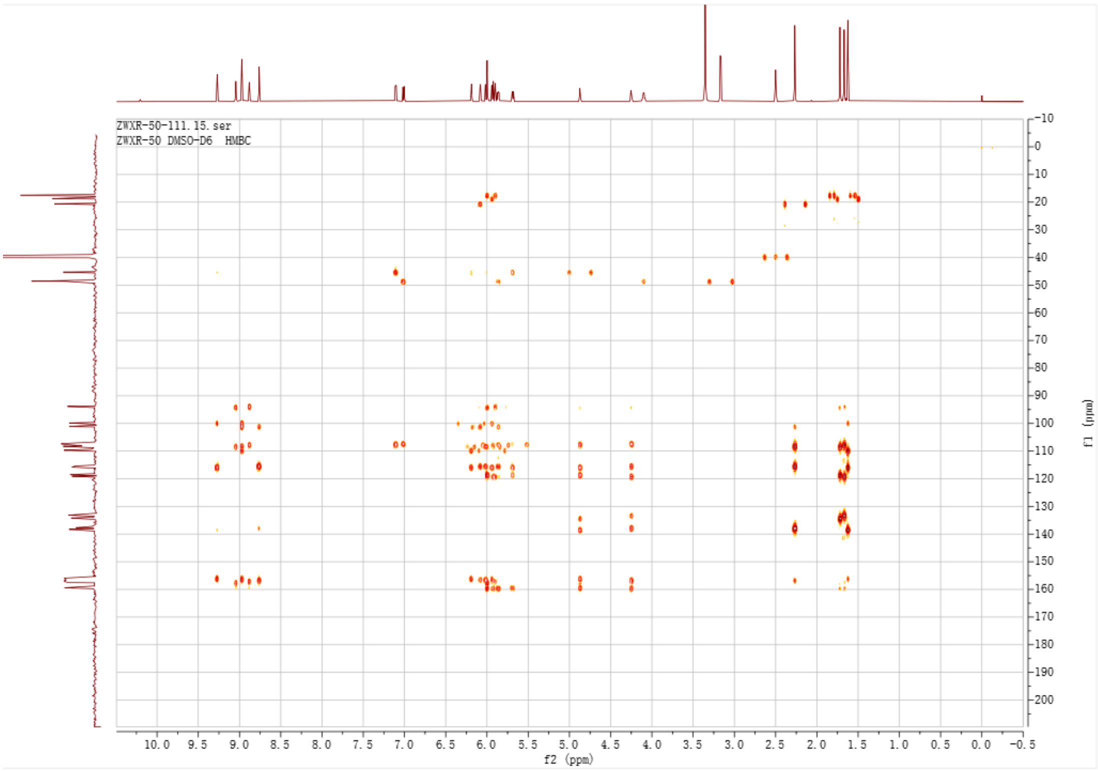


NOESY spectrum of compound **5**

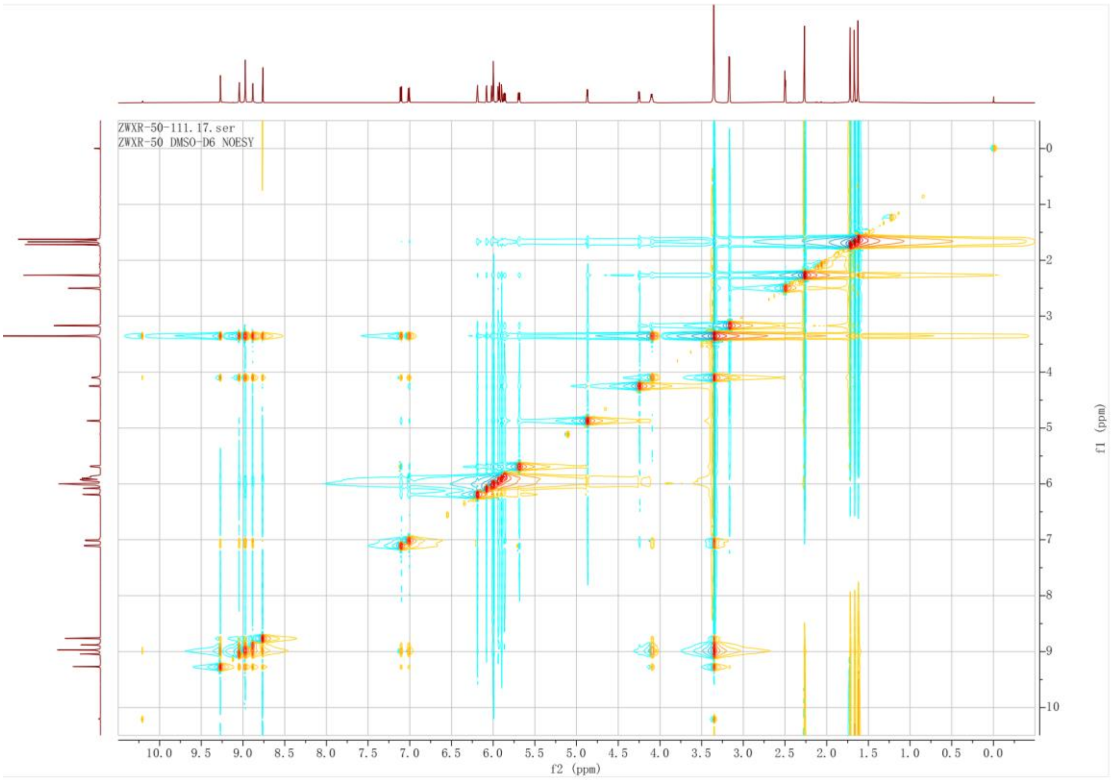

HRESIMS plot of compound **5**

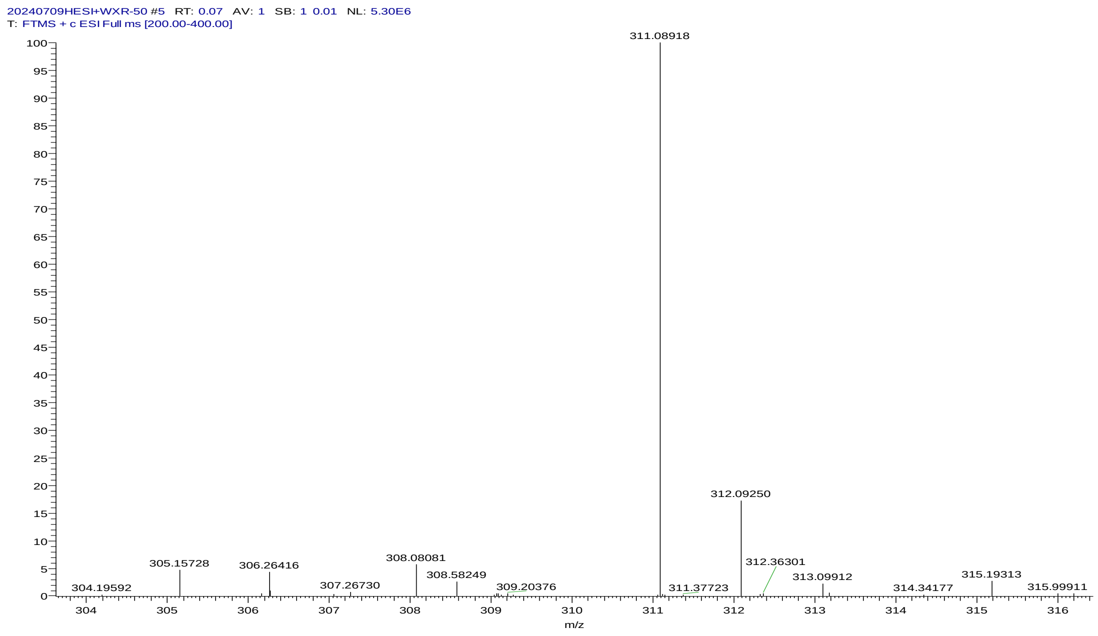

IR spectrum of compound **5**

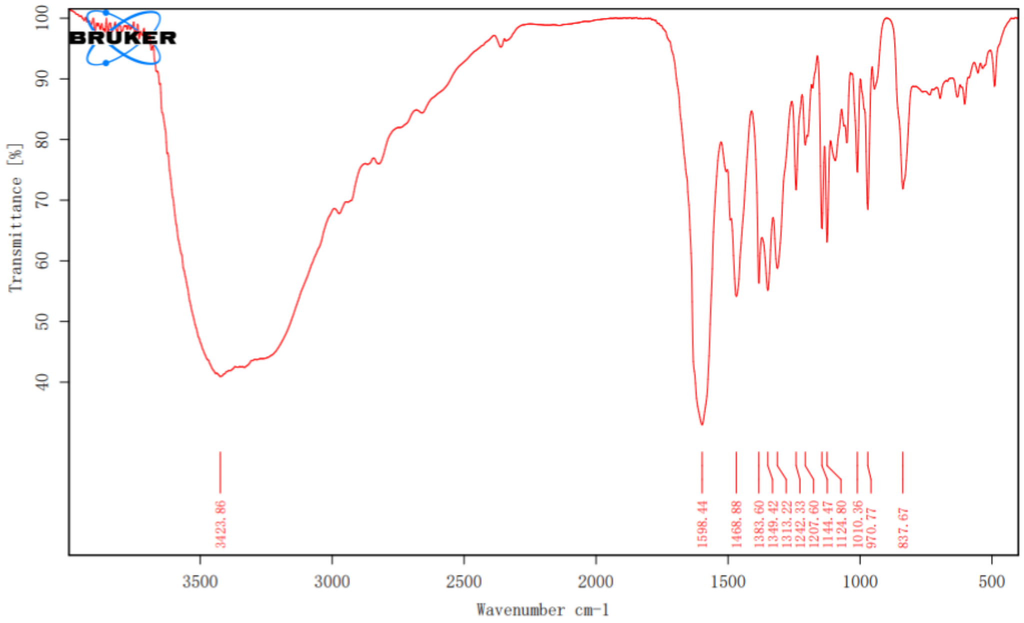


UV spectrum of compound **5**

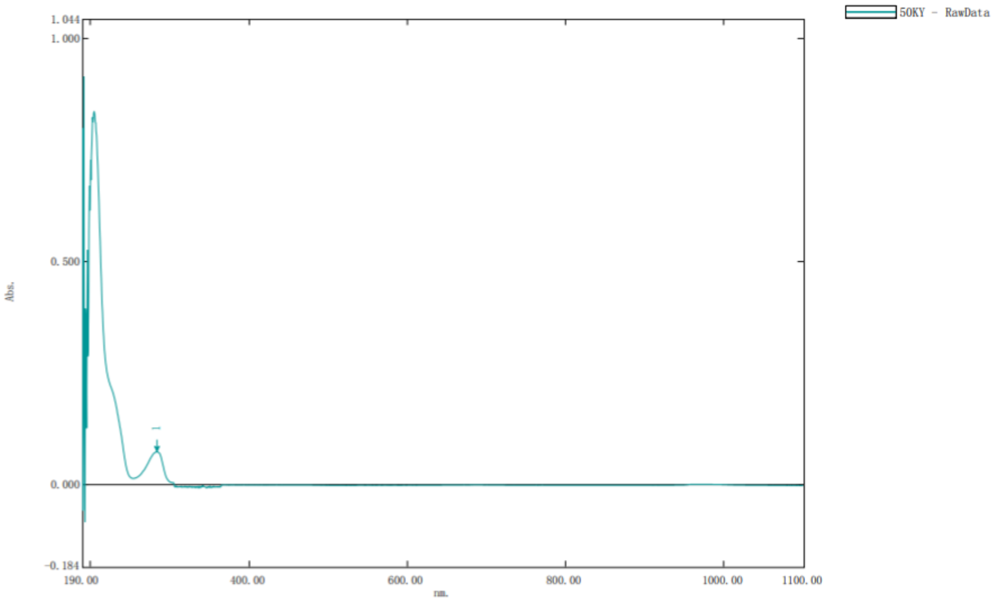

$^{1}$H-NMR spectrum of compound **6** (600 MHz, $CD_3OD$)

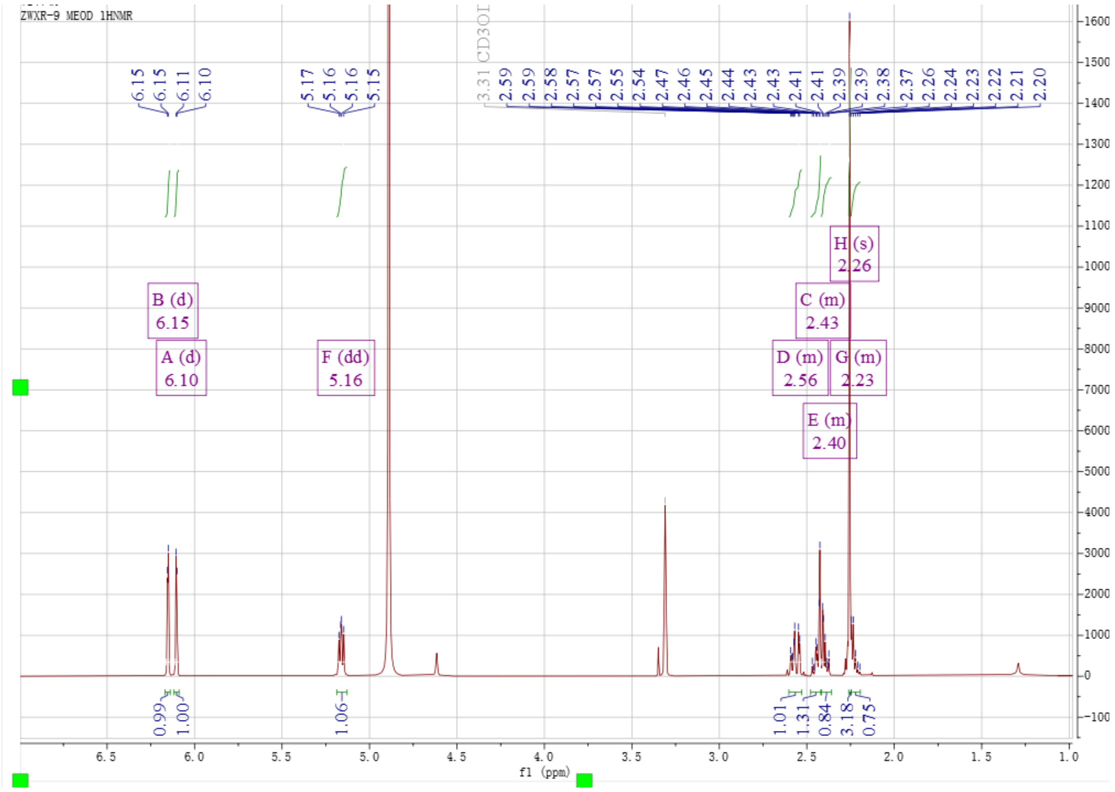

$^{13}$C-NMR spectrum of compound **6** (151 MHz, $CD_3OD$)

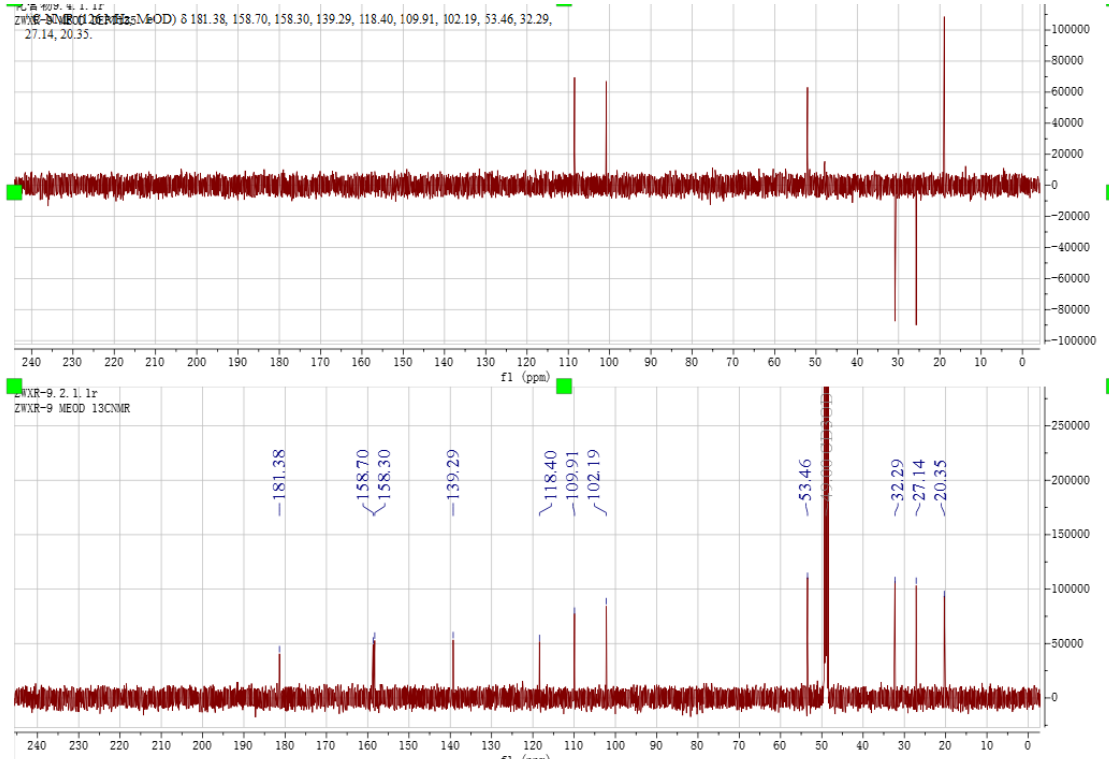

$^{1}$H-$^{1}$H COSY spectrum of compound **6**

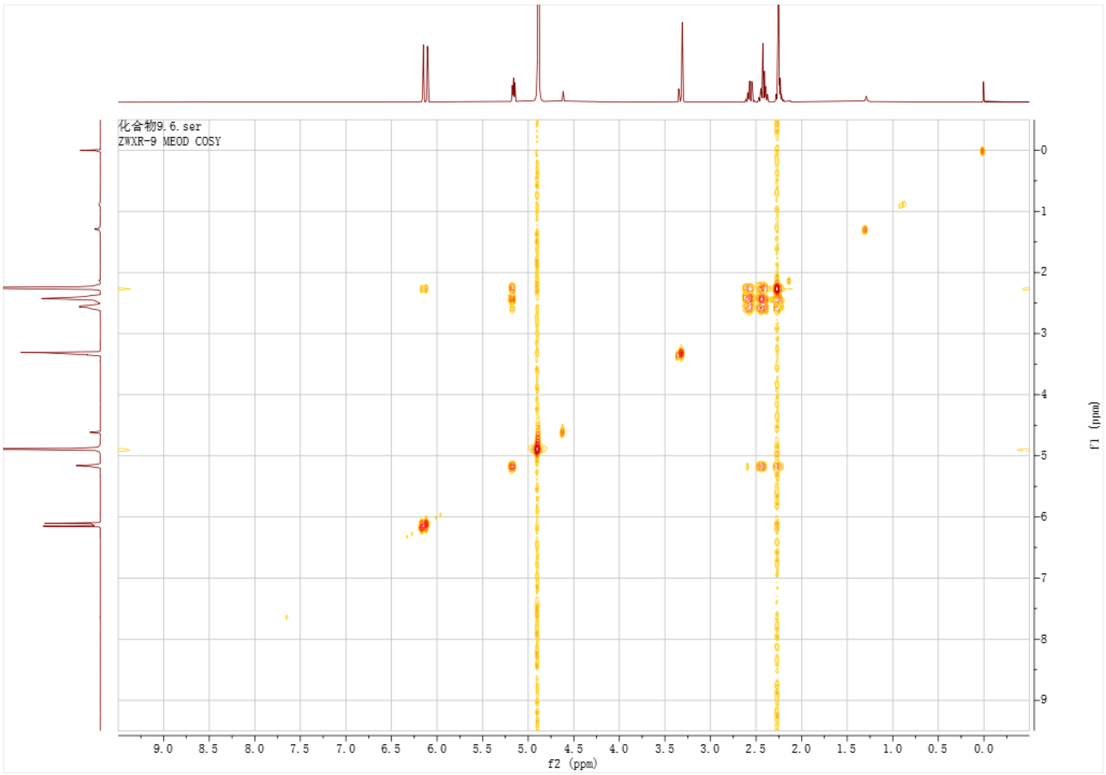


HSQC spectrum of compound **6**

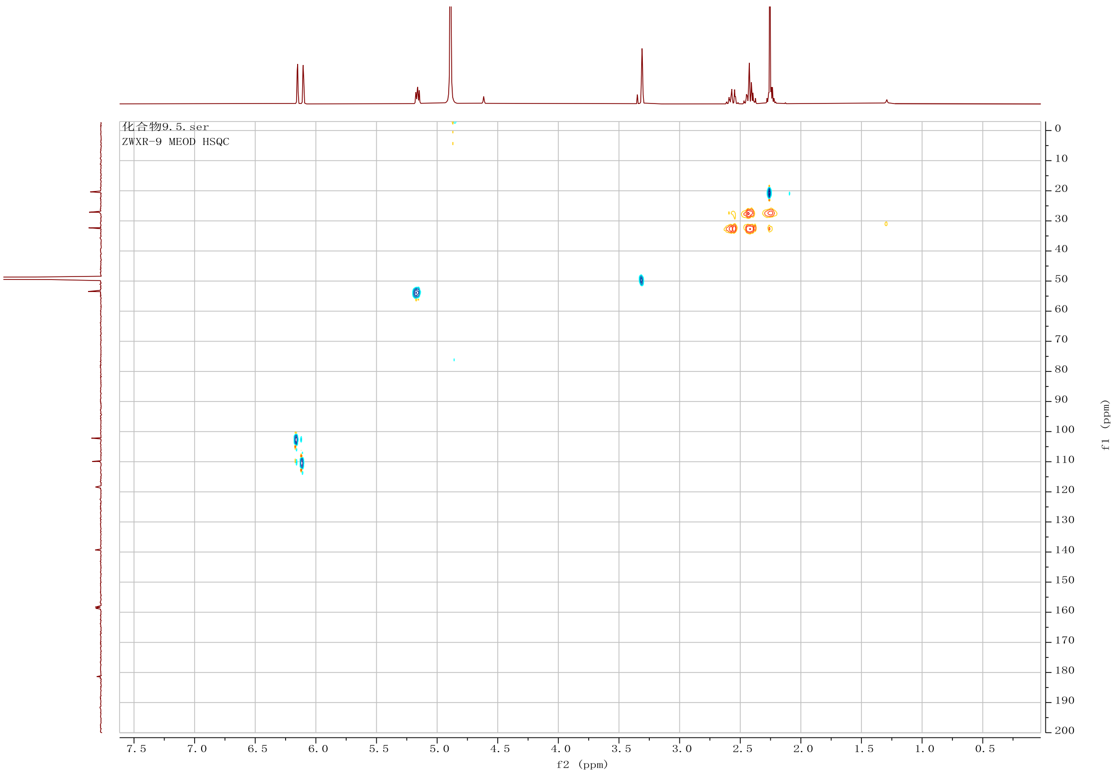

HMBC spectrum of compound **6**

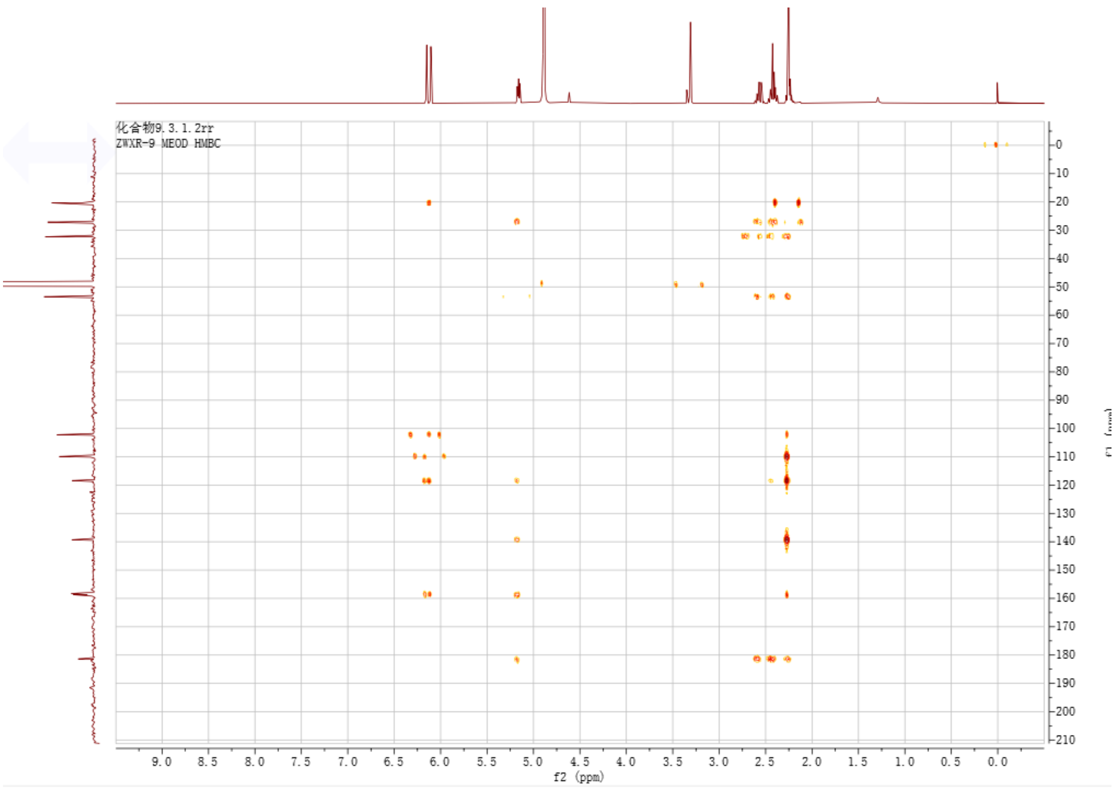


ROESY spectrum of compound **6**

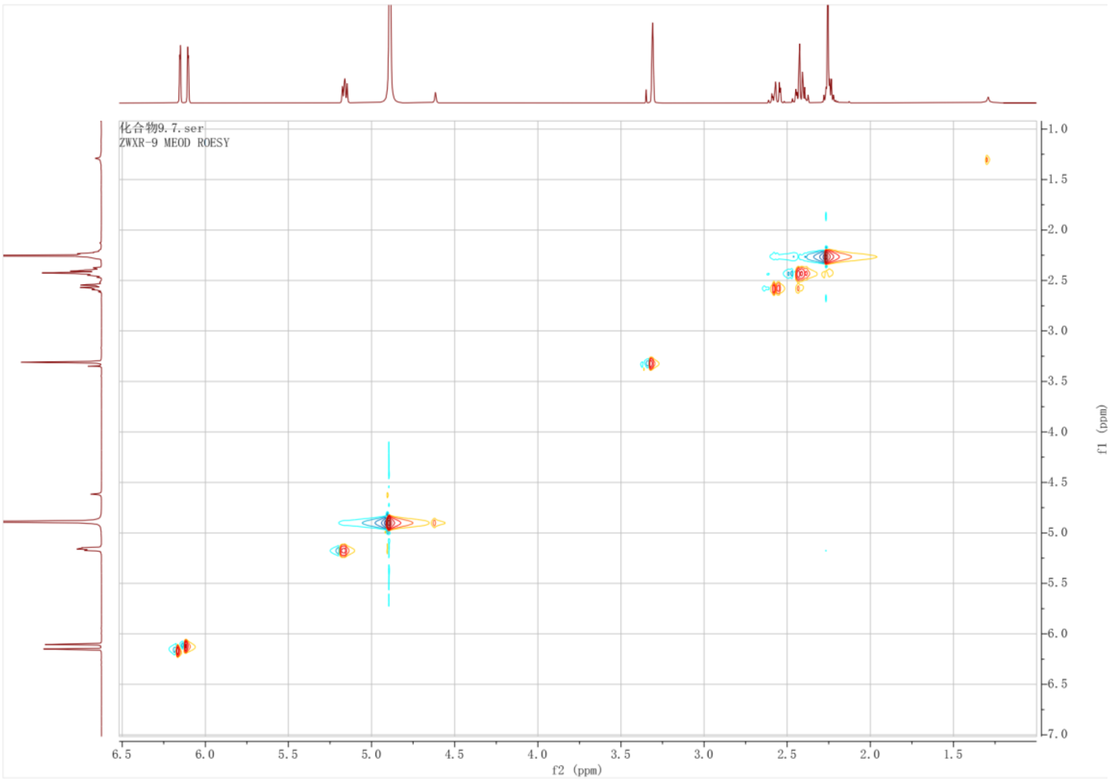

HRESIMS plot of compound **6**

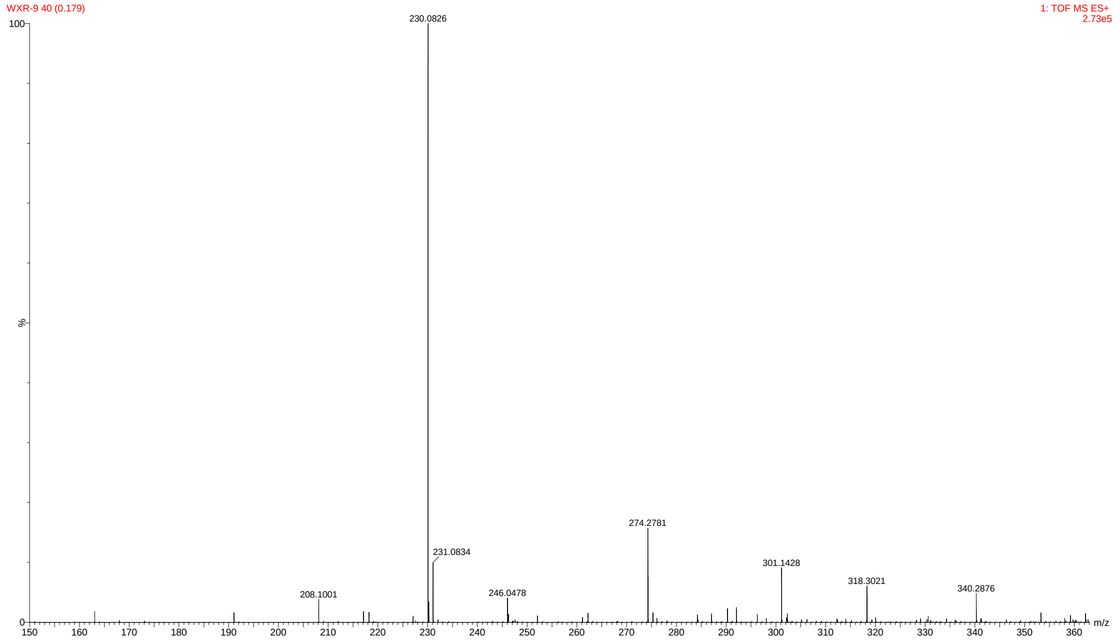

IR spectrum of compound **6**

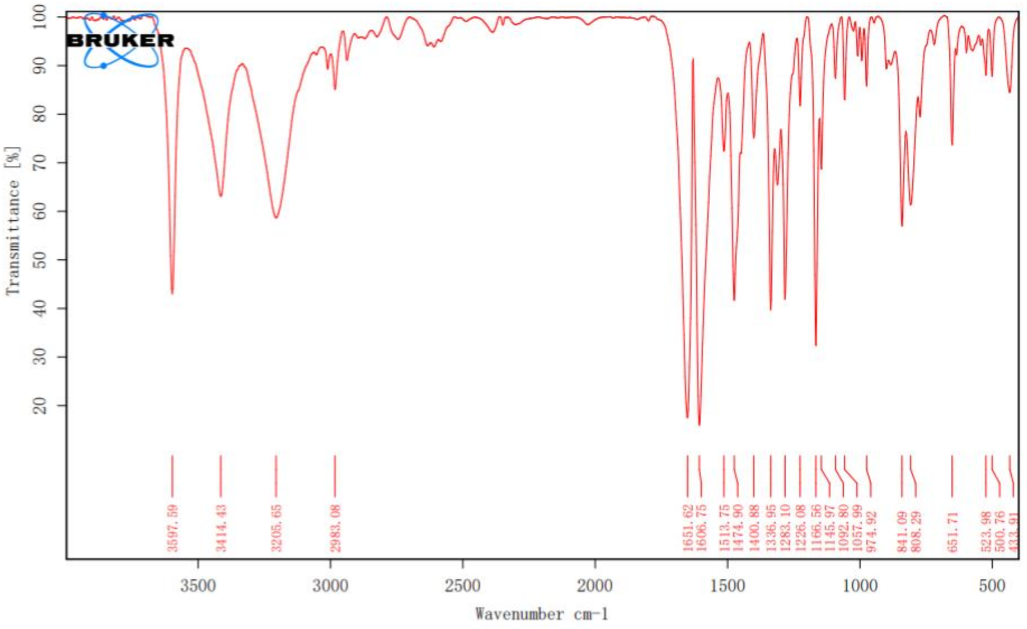


UV spectrum of compound **6**

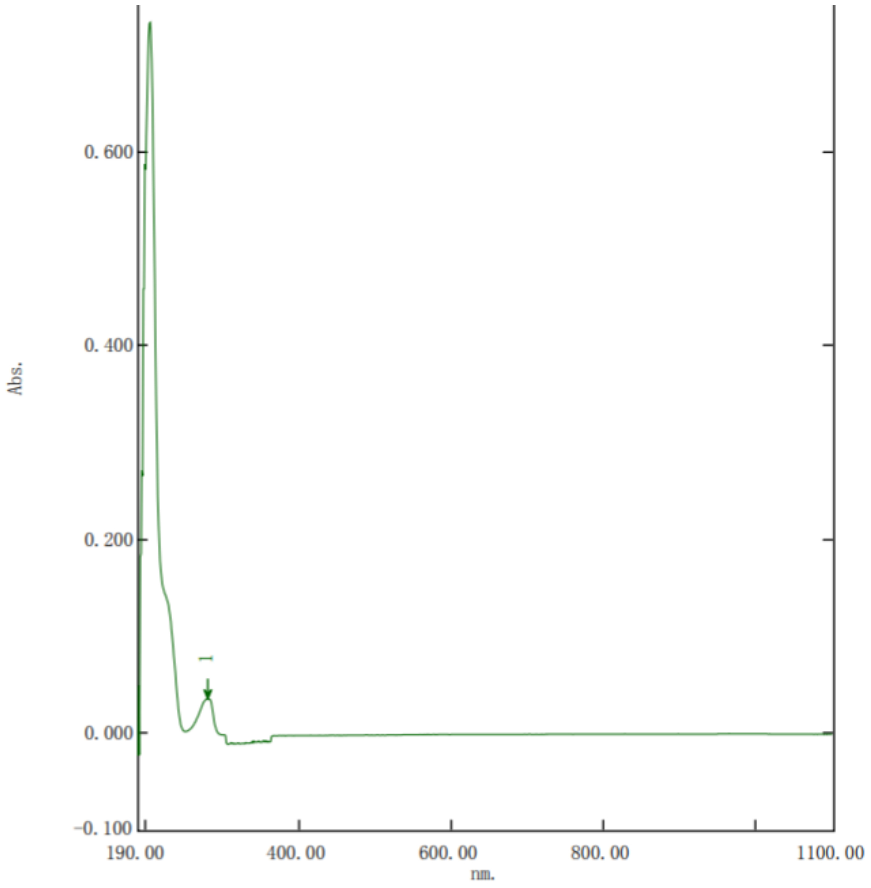

<sup></sup>$^{1}$H-NMR spectrum of compound **7** (700 MHz, $CD_3OD$)

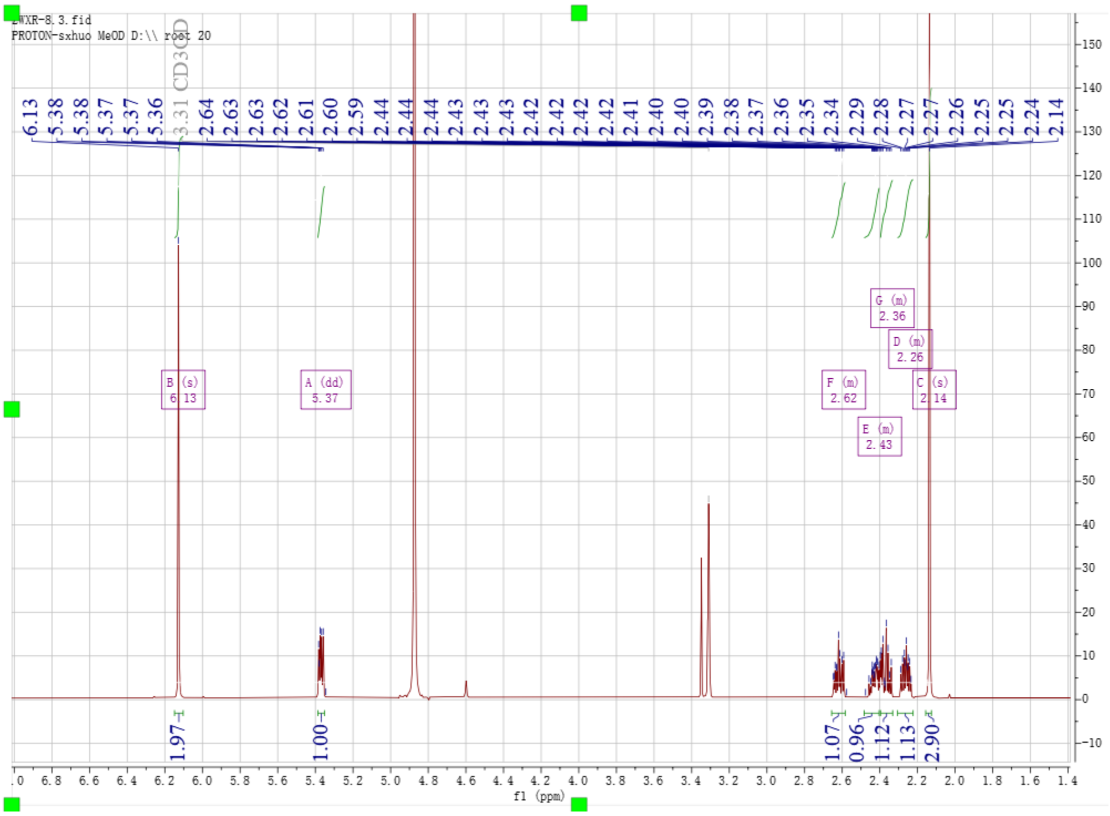


$^{13}$C-NMR spectrum of compound **7** (151 MHz, $CD_3OD$)

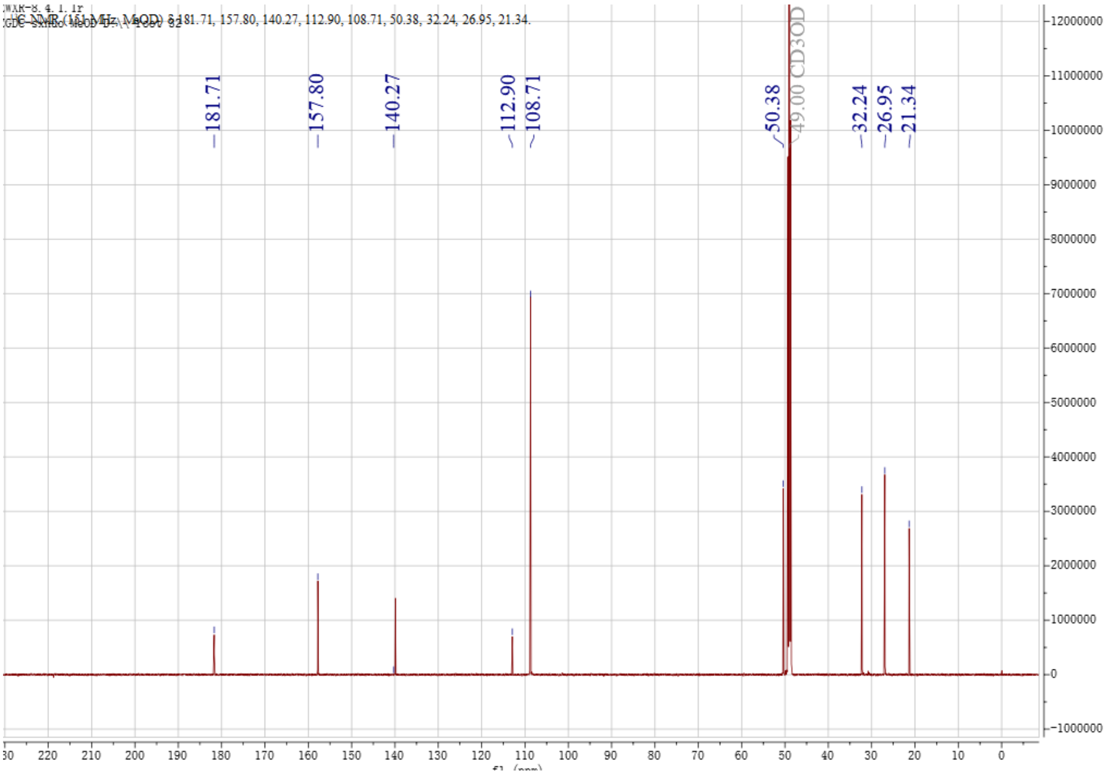

$^1$H-$^1$H COSY spectrum of compound **7**

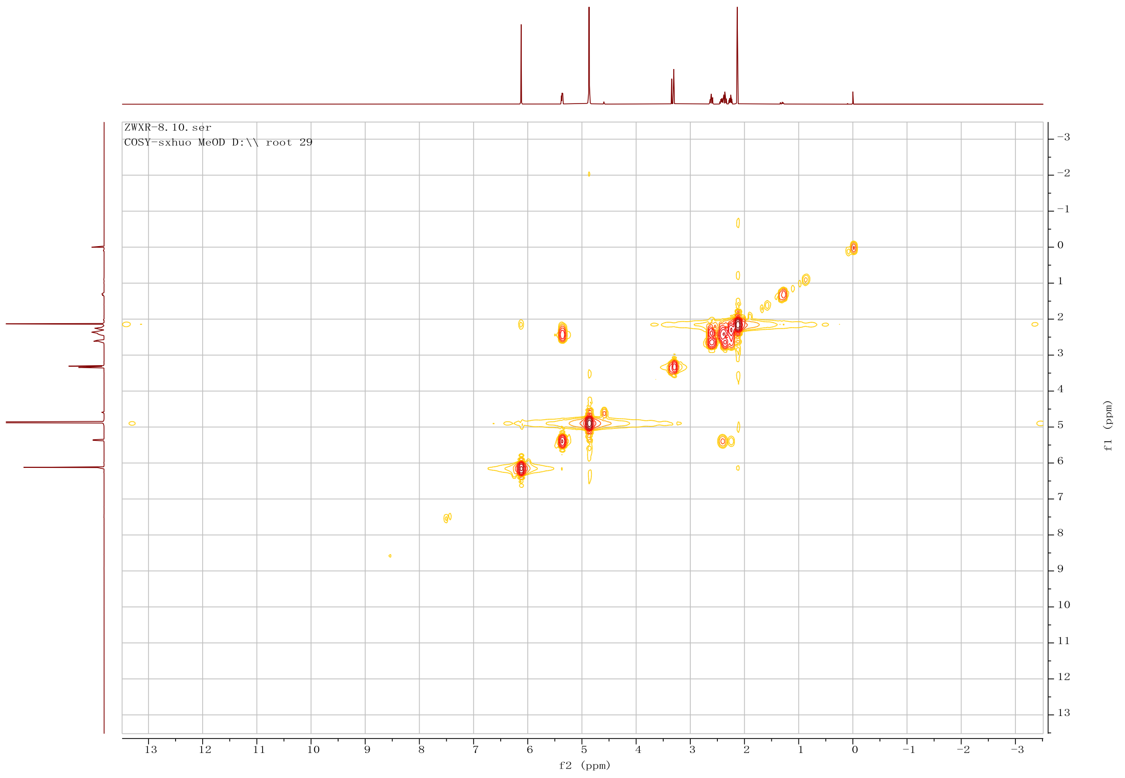


HSQC spectrum of compound **7**

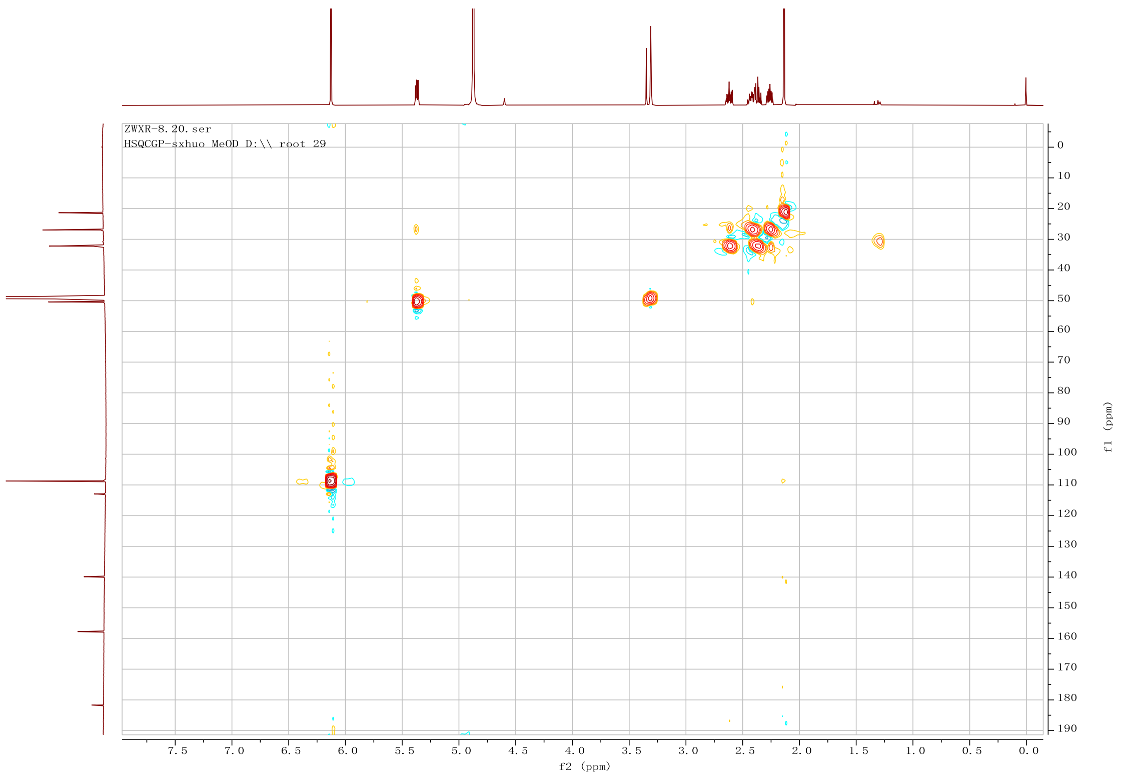

HMBC spectrum of compound **7**

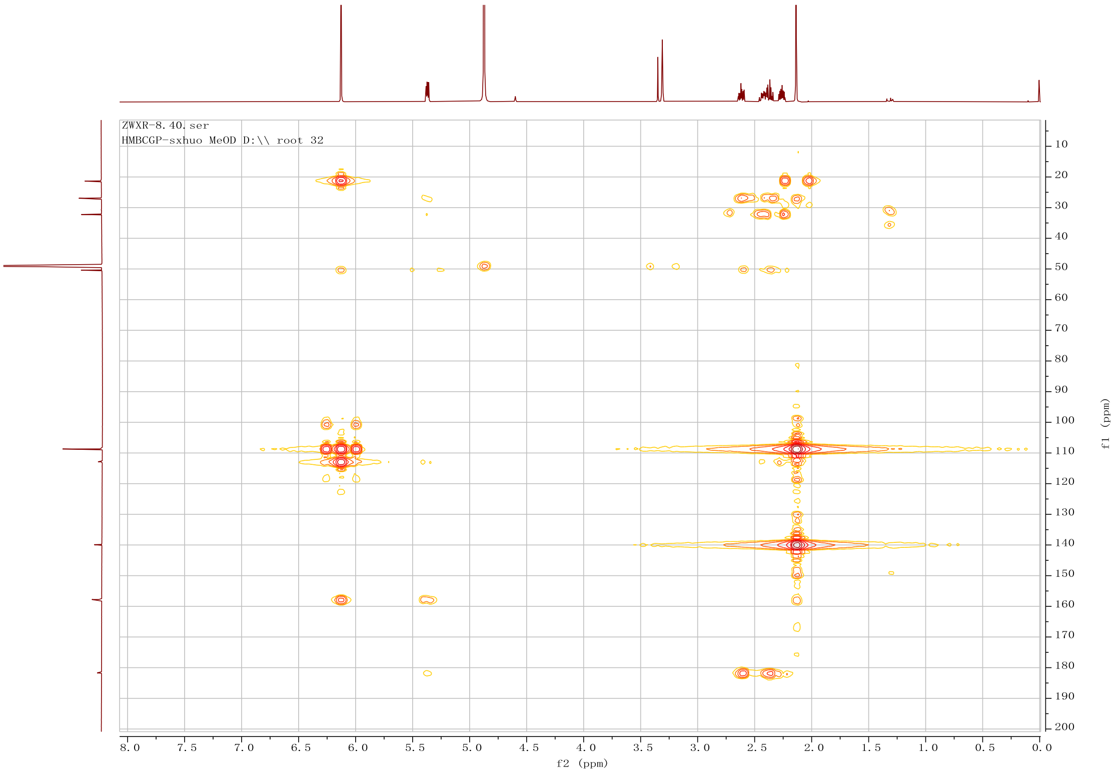

HRESIMS plot of compound **7**

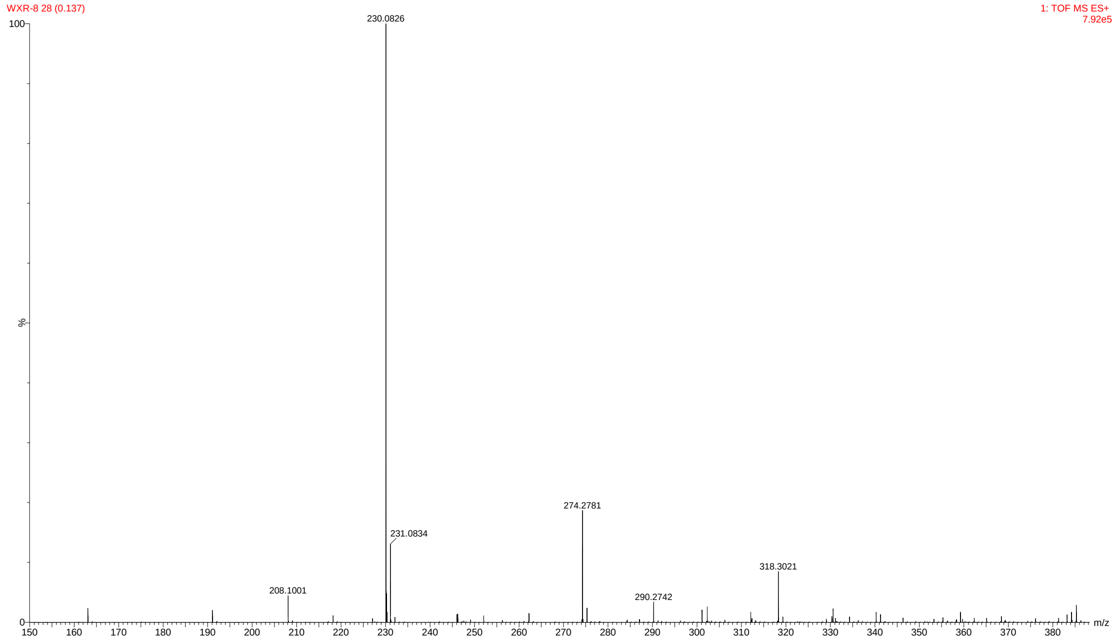

IR spectrum of compound **7**

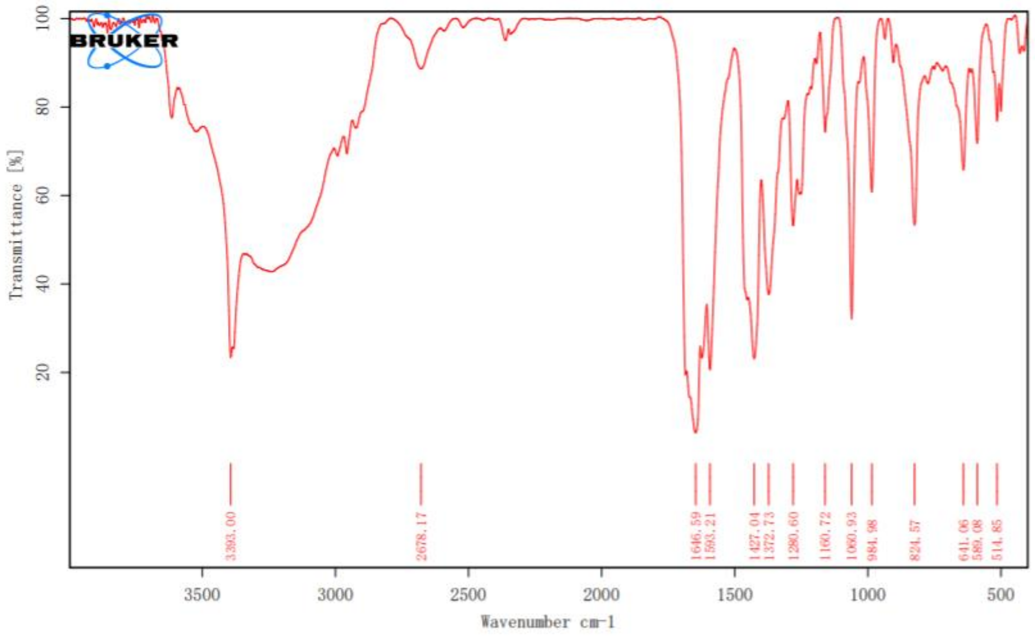


UV spectrum of compound **7**

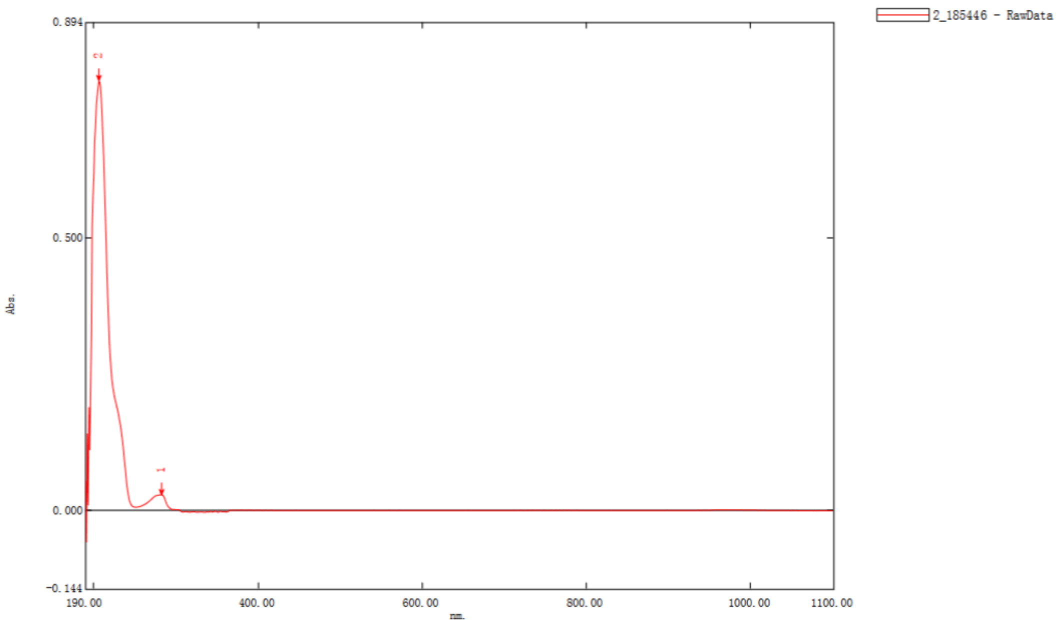

$^{1}$H-NMR spectrum of compound **8** (600 MHz, $CD_3OD$)

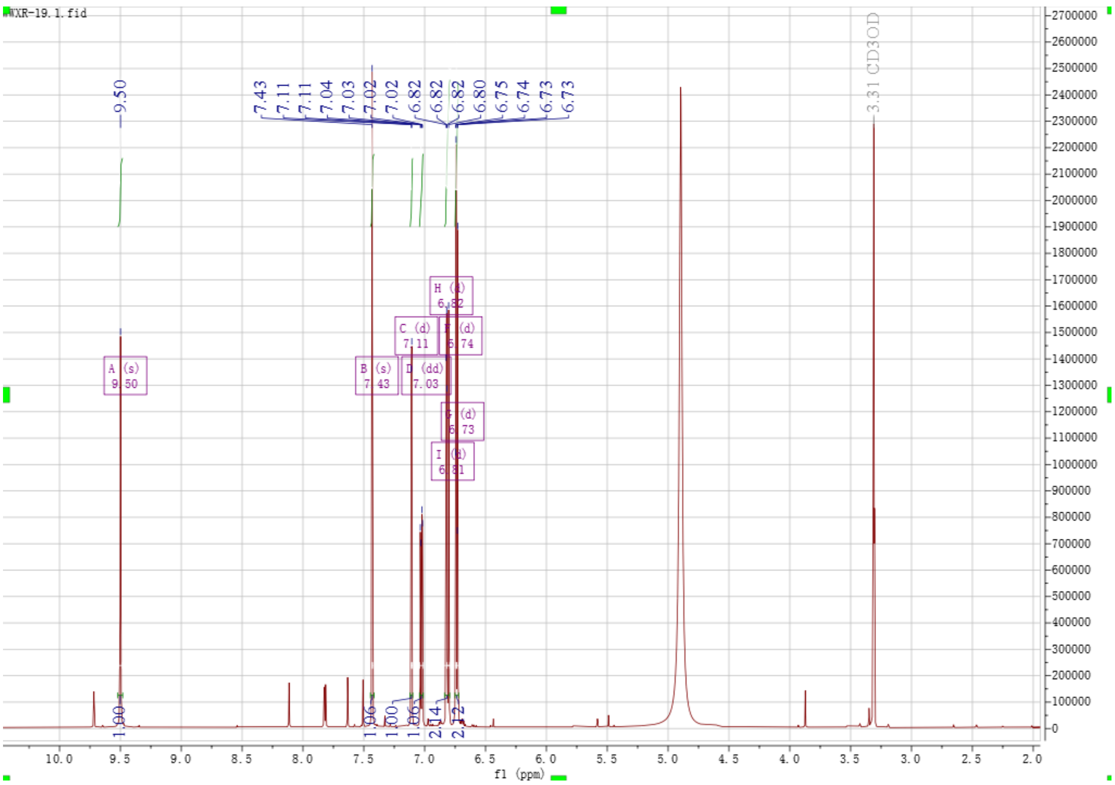

$^{13}$C-NMR and $^{13}$C DEPT-135 spectra of compound **8** (151 MHz, $CD_3OD$)

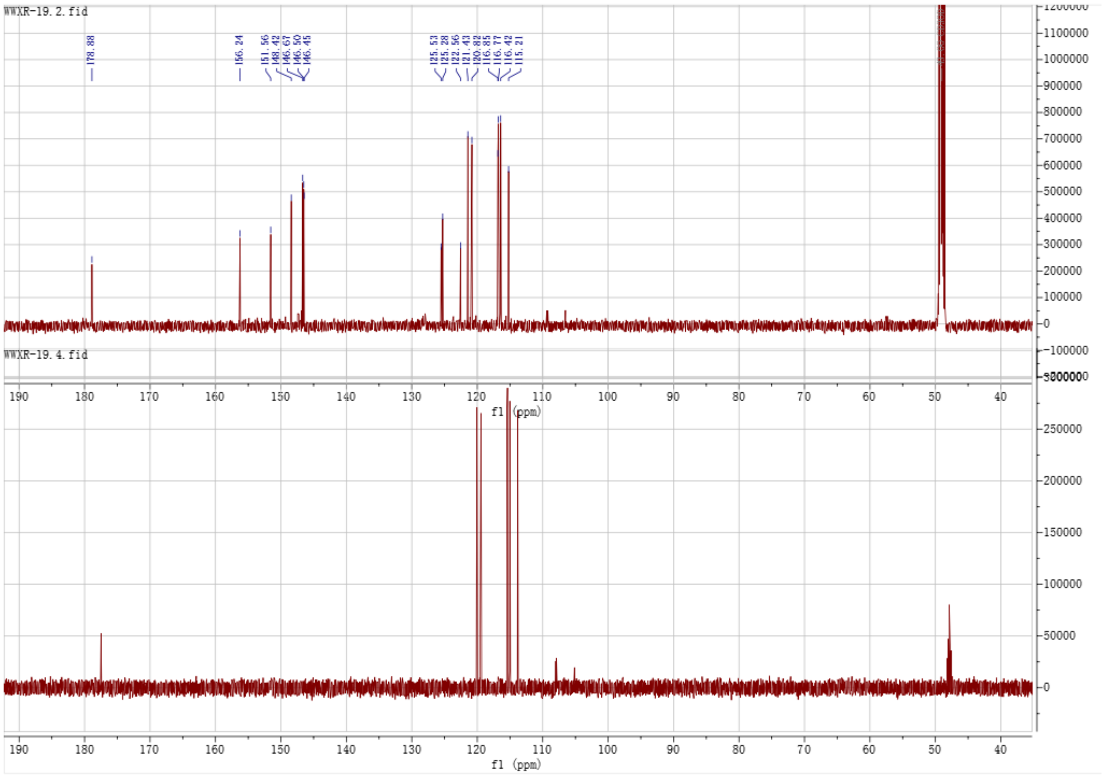

$^{1}$H-NMR spectrum of compound **9** (600 MHz, $CD_3OD$)

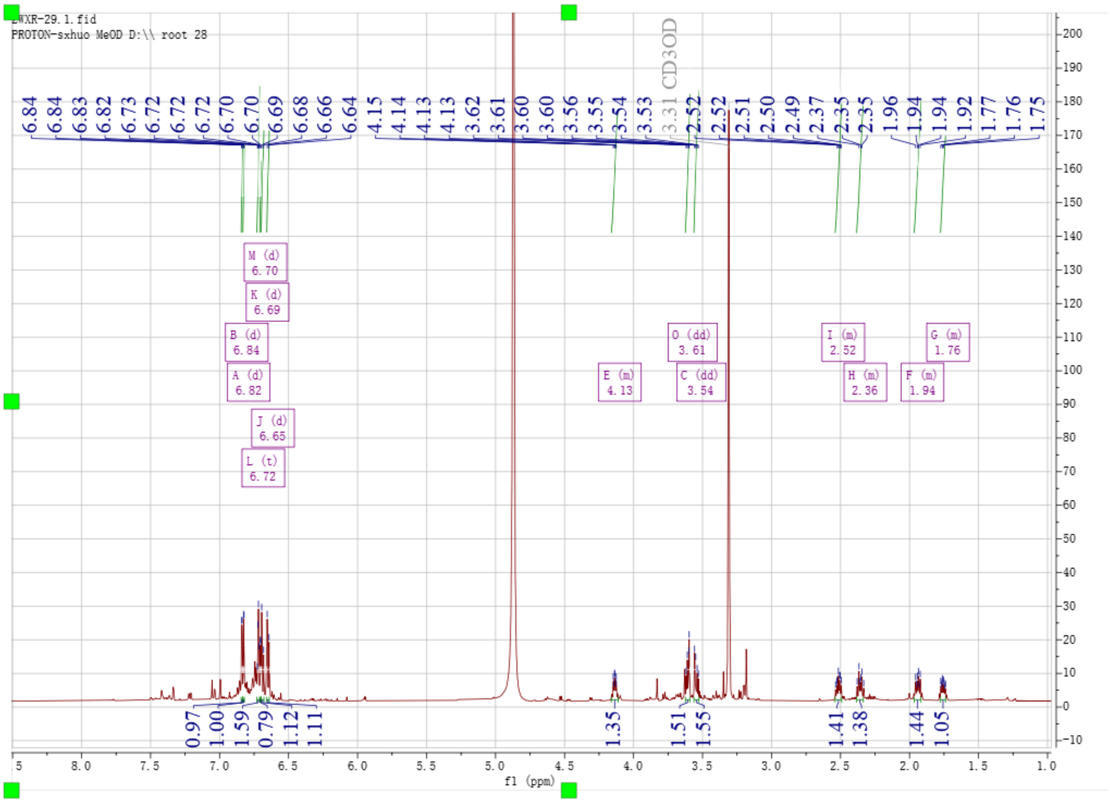

$^{13}$C-NMR and $^{13}$C DEPT-135 spectra of compound **9** (151 MHz, $CD_3OD$)

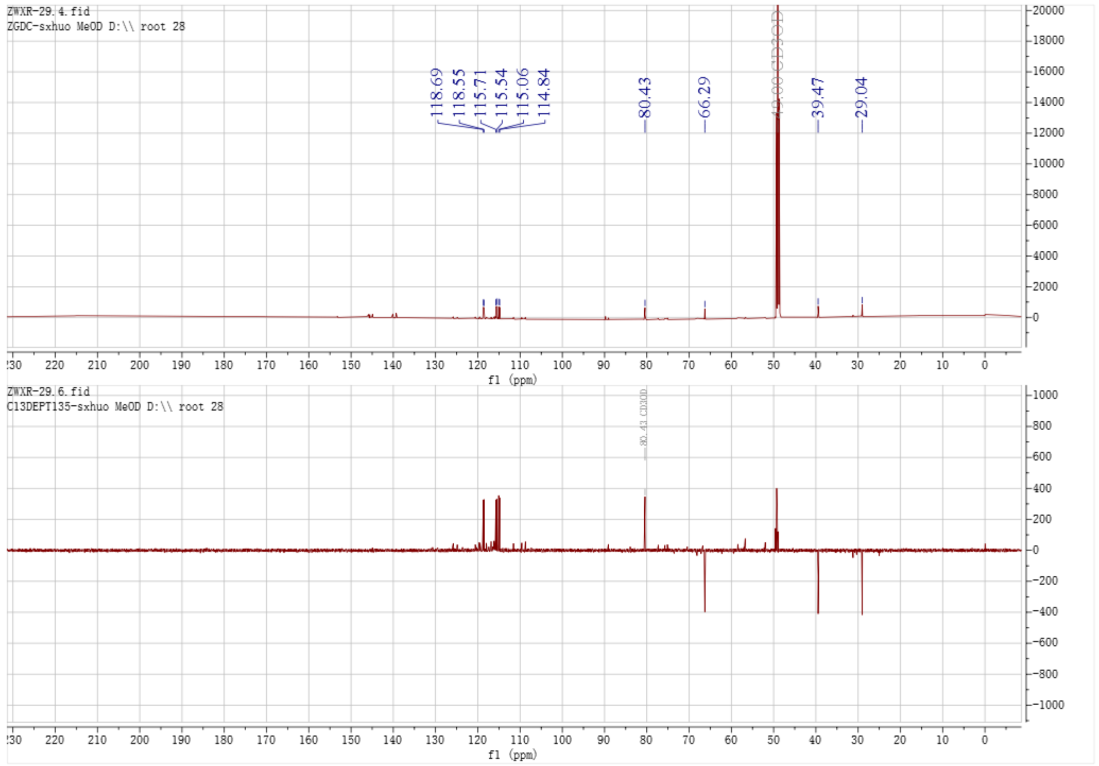

$^{1}$H-NMR spectrum of compound **10** (600 MHz, $CD_3OD$)

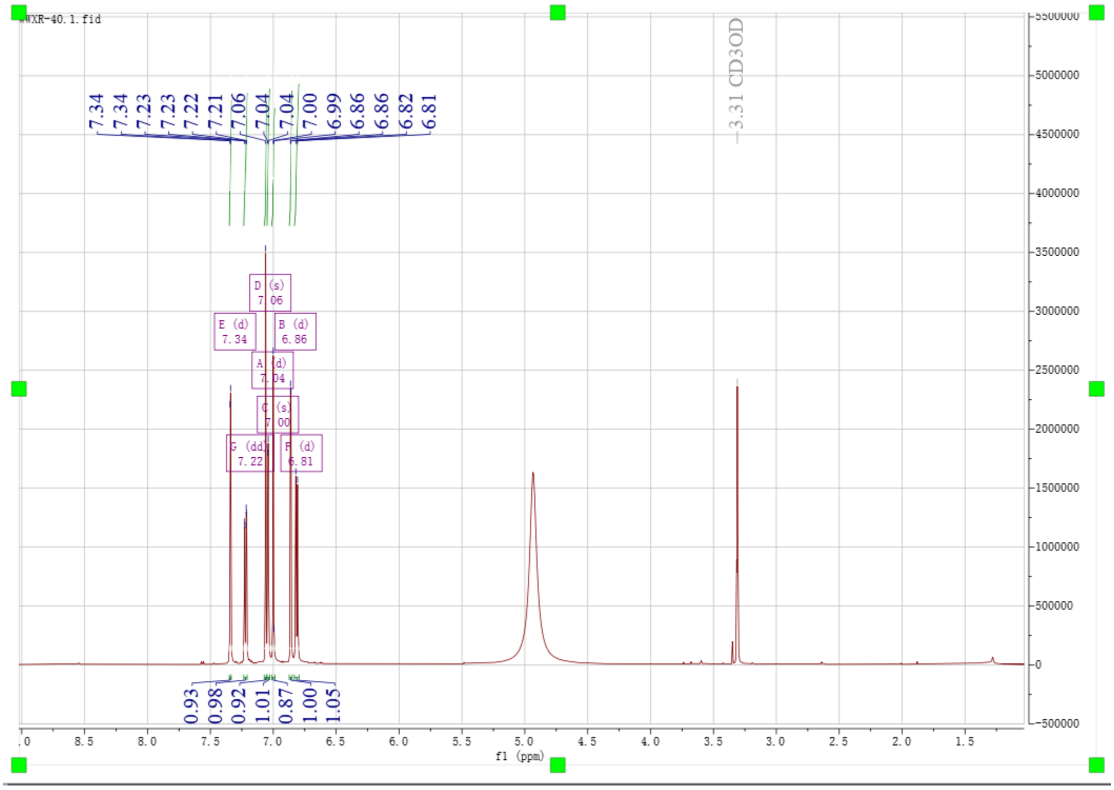


$^{13}$C-NMR and $^{13}$C DEPT-135 spectra of compound **10** (151 MHz, $CD_3OD$)

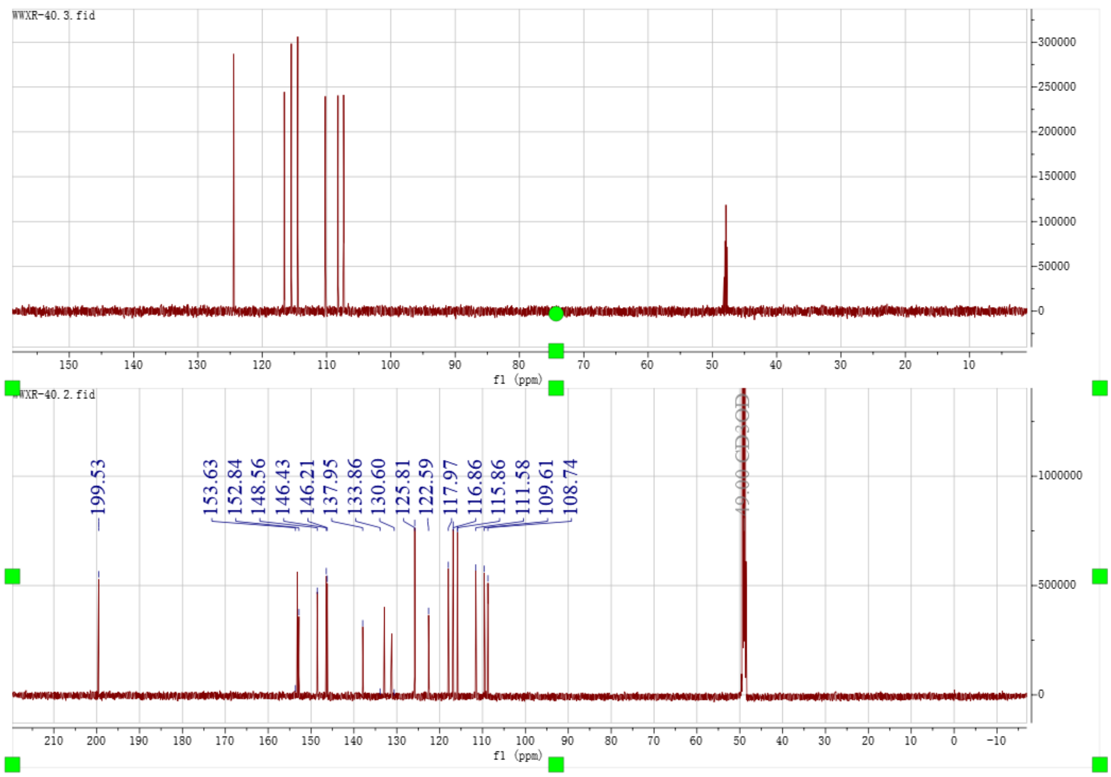

$^{1}$H-NMR spectrum of compound **11** (600 MHz, $CD_3OD$)

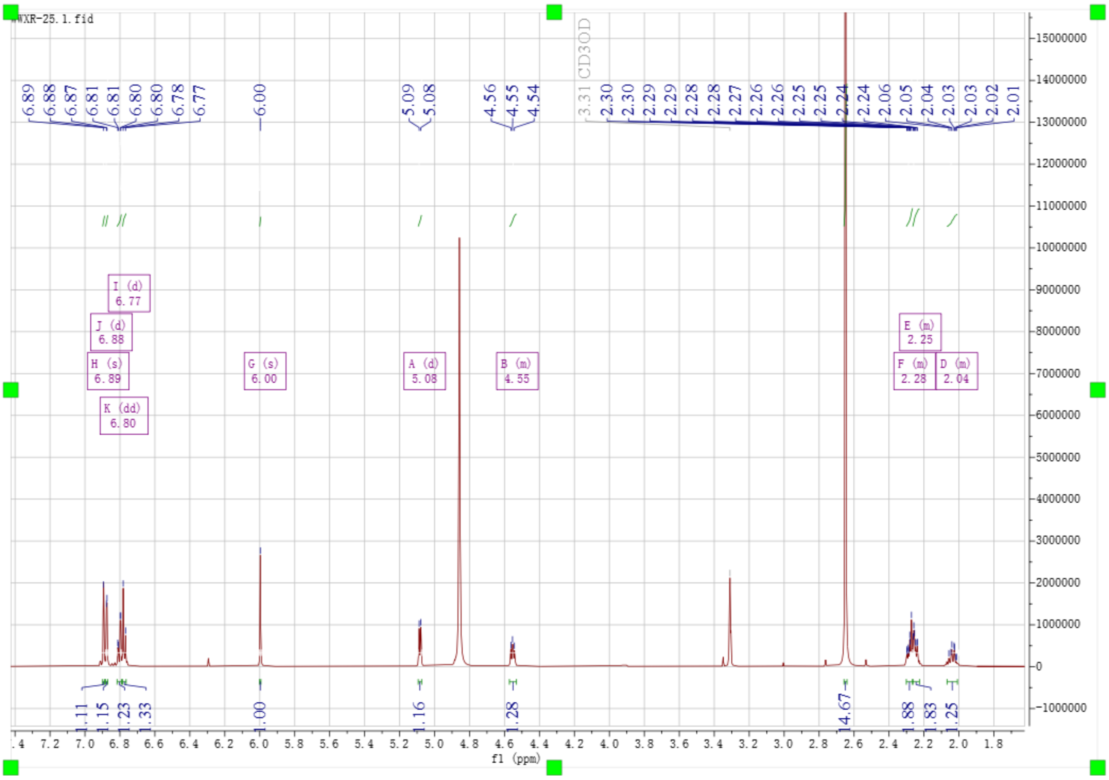


$^{13}$C-NMR and $^{13}$C DEPT-135 spectra of compound **11** (151 MHz, $CD_3OD$)

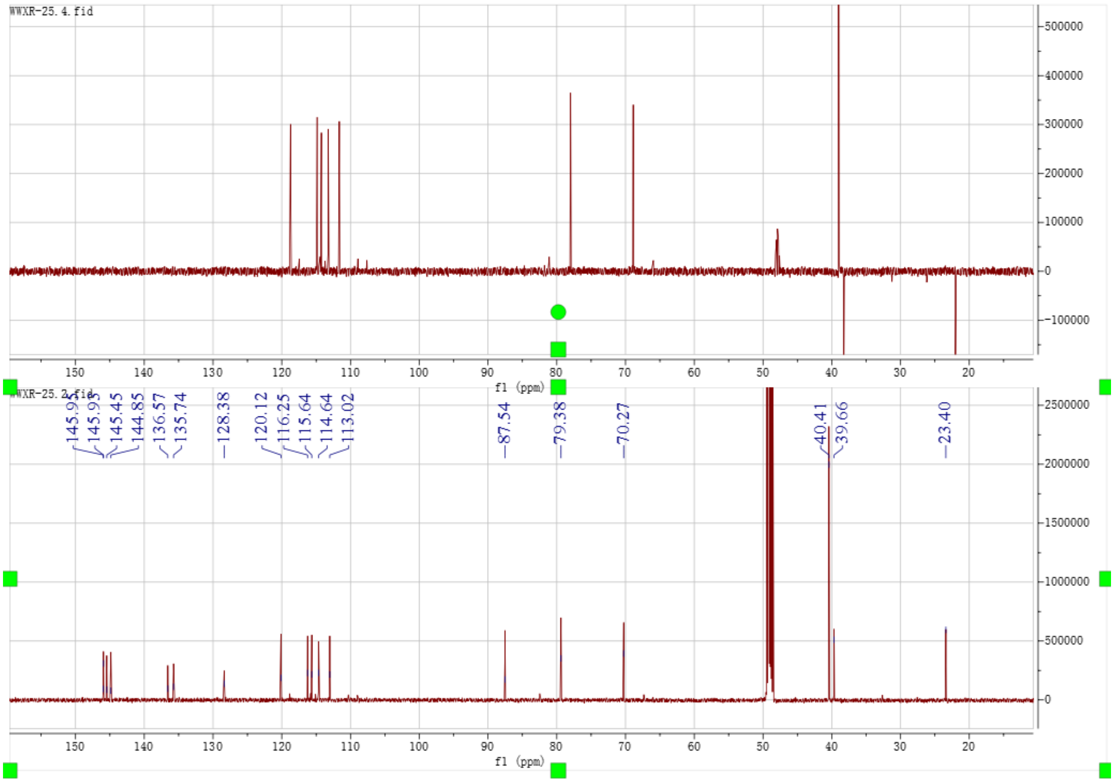

$^1$H-NMR spectrum of compound **12** (600 MHz, $CD_3OD$)

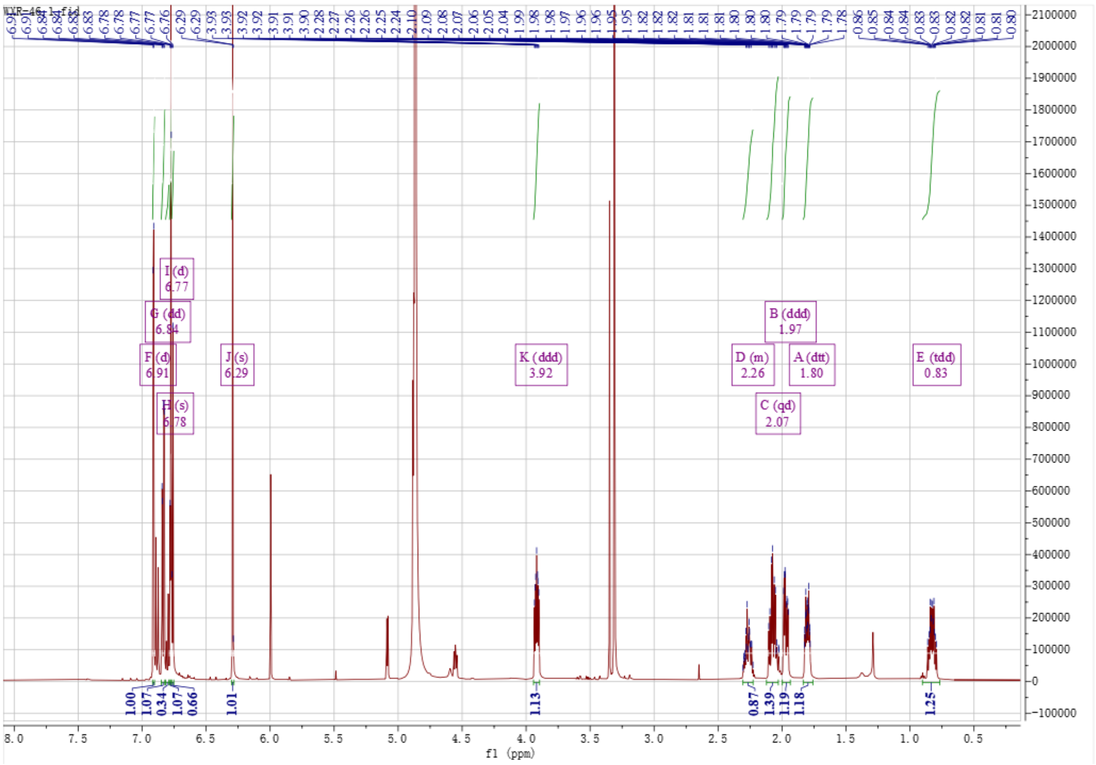

$^{13}$C-NMR and $^{13}$C DEPT-135 spectra of compound **12** (151 MHz, $CD_3OD$)

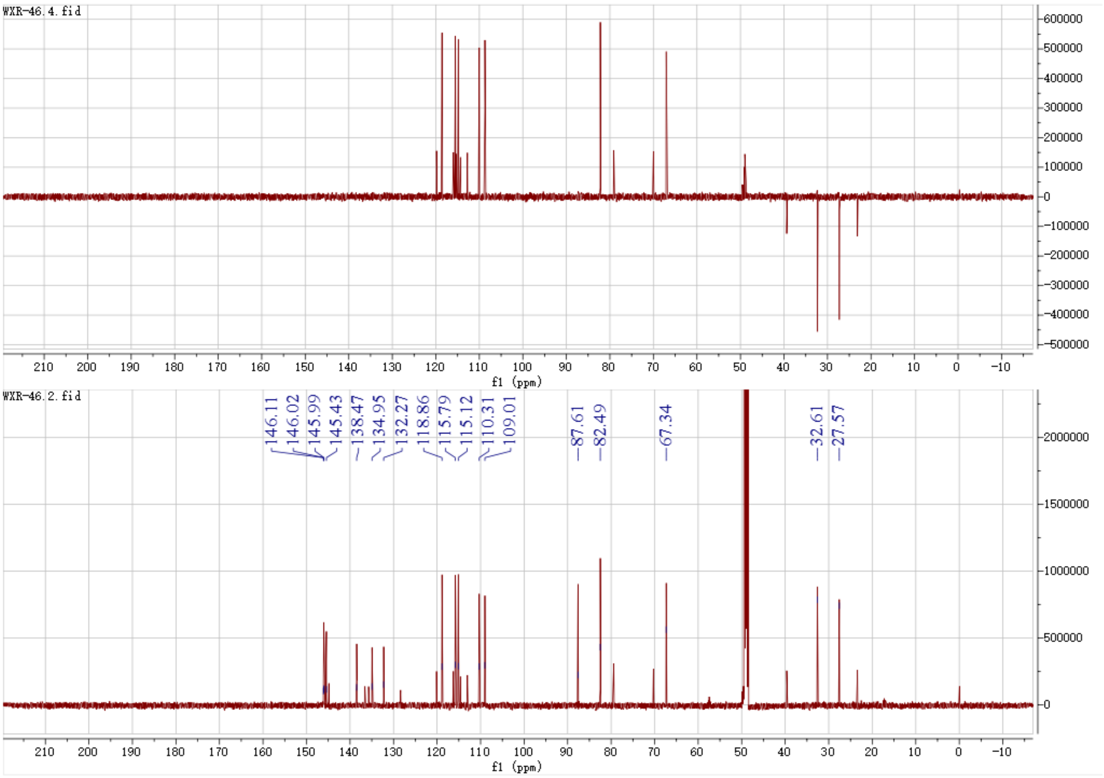

$^{1}$H-NMR spectrum of compound **13** (600 MHz, $CD_3OD$)

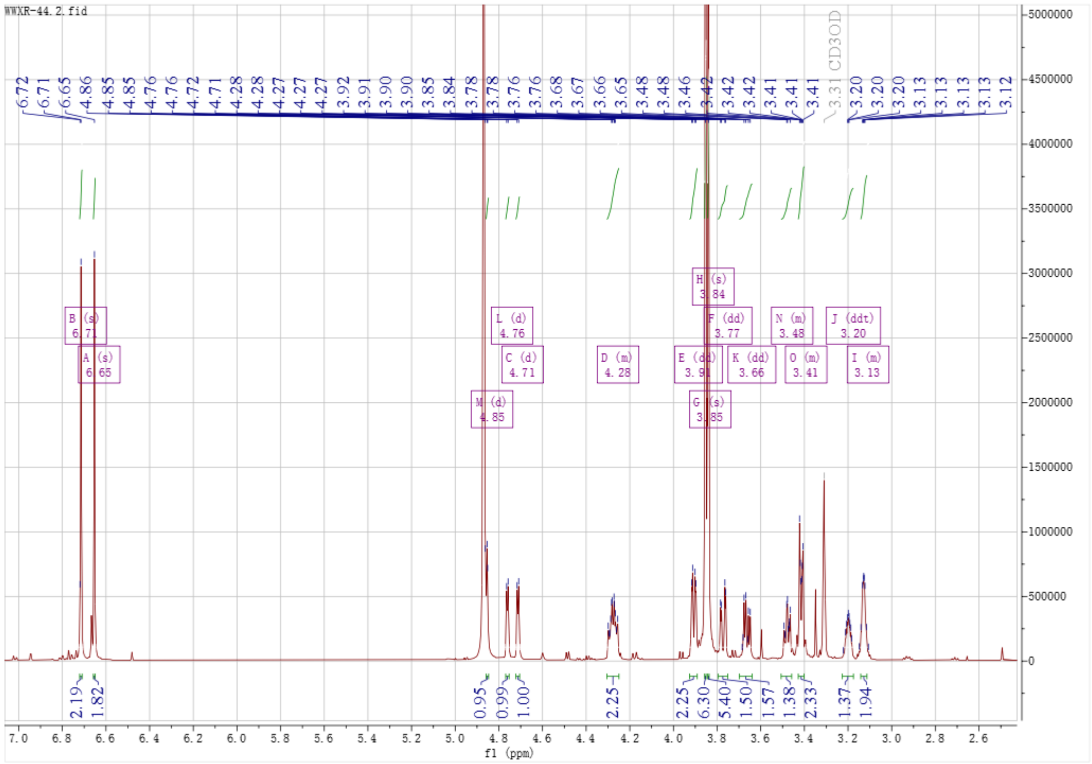

$^{13}$C-NMR and $^{13}$C DEPT-135 spectra of compound **13** (151 MHz, $CD_3OD$)

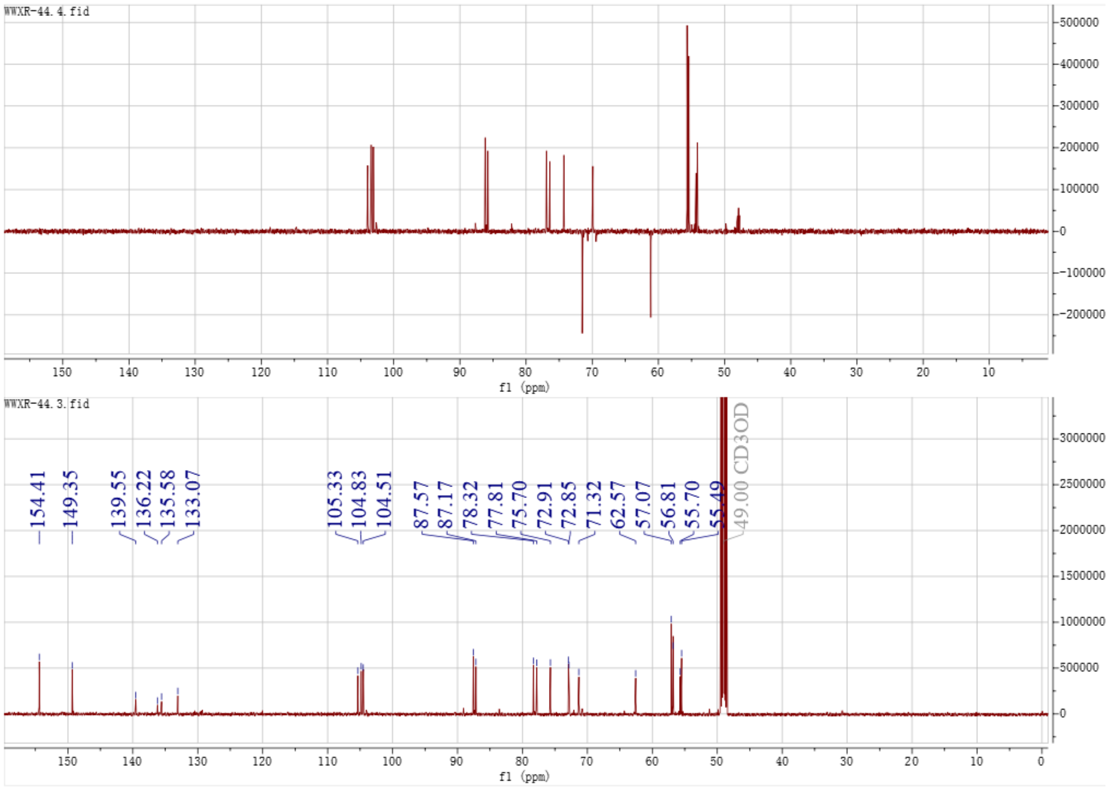

$^{1}$H-NMR spectrum of compound **14** (600 MHz, $CD_3OD$)

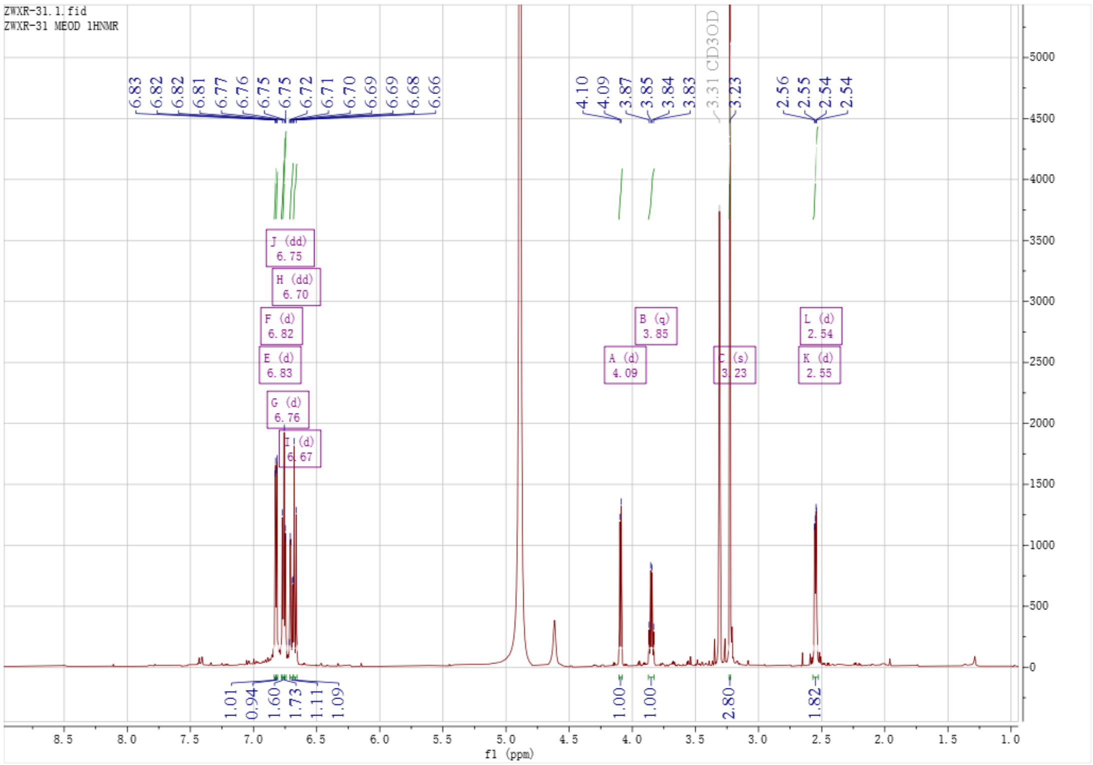


$^{14}$C-NMR and $^{14}$C DEPT-135 spectra of compound **14** (151 MHz, $CD_3OD$)

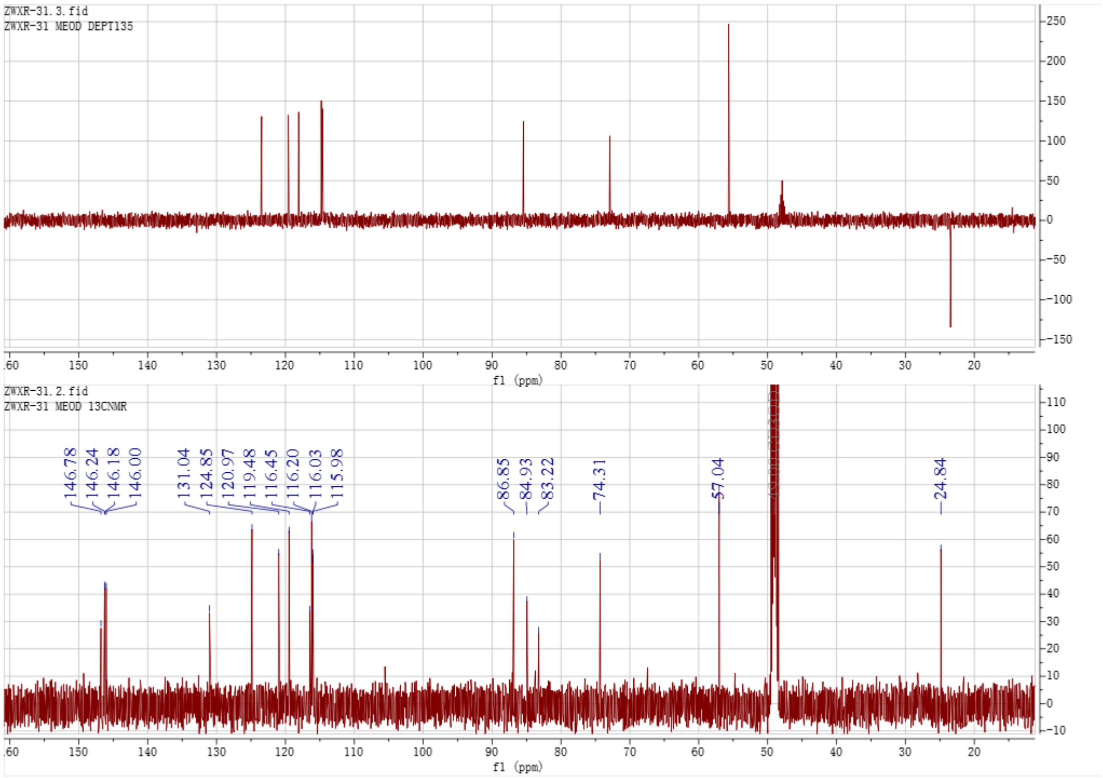

$^{1}$H-NMR spectrum of compound **15** (600 MHz, $CD_3OD$)

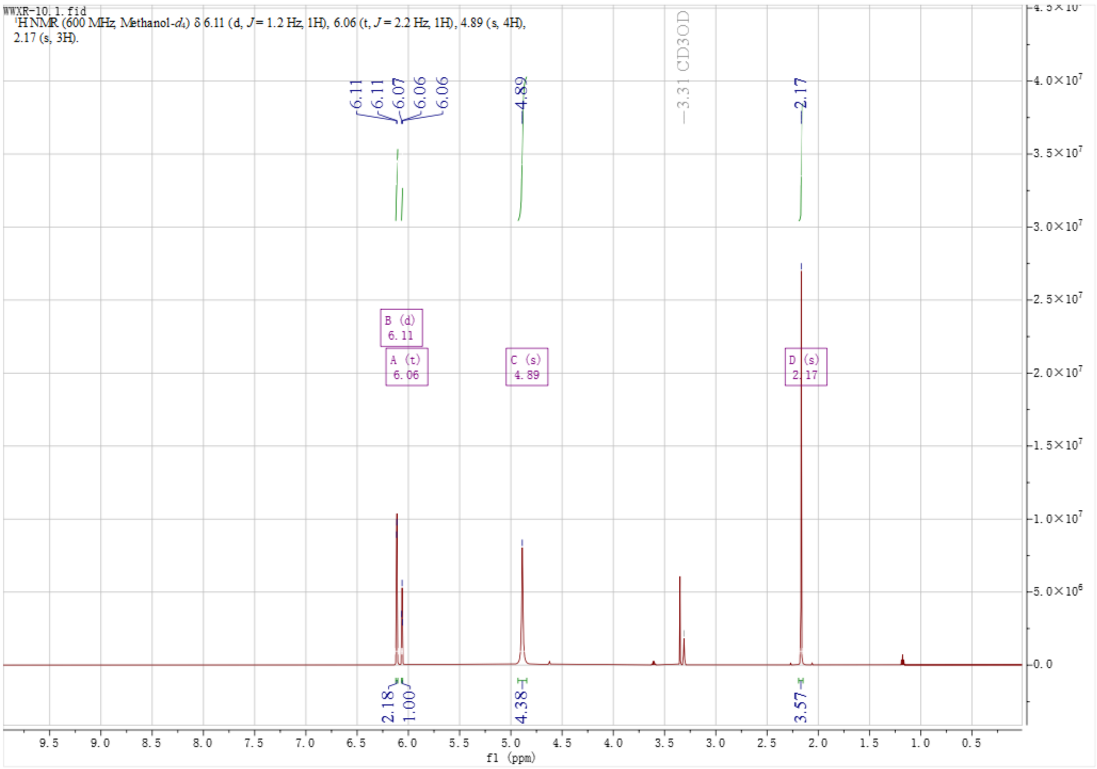


[15]C-NMR and [15]C DEPT-135 spectra of compound **15** (151 MHz, $CD_3OD$)

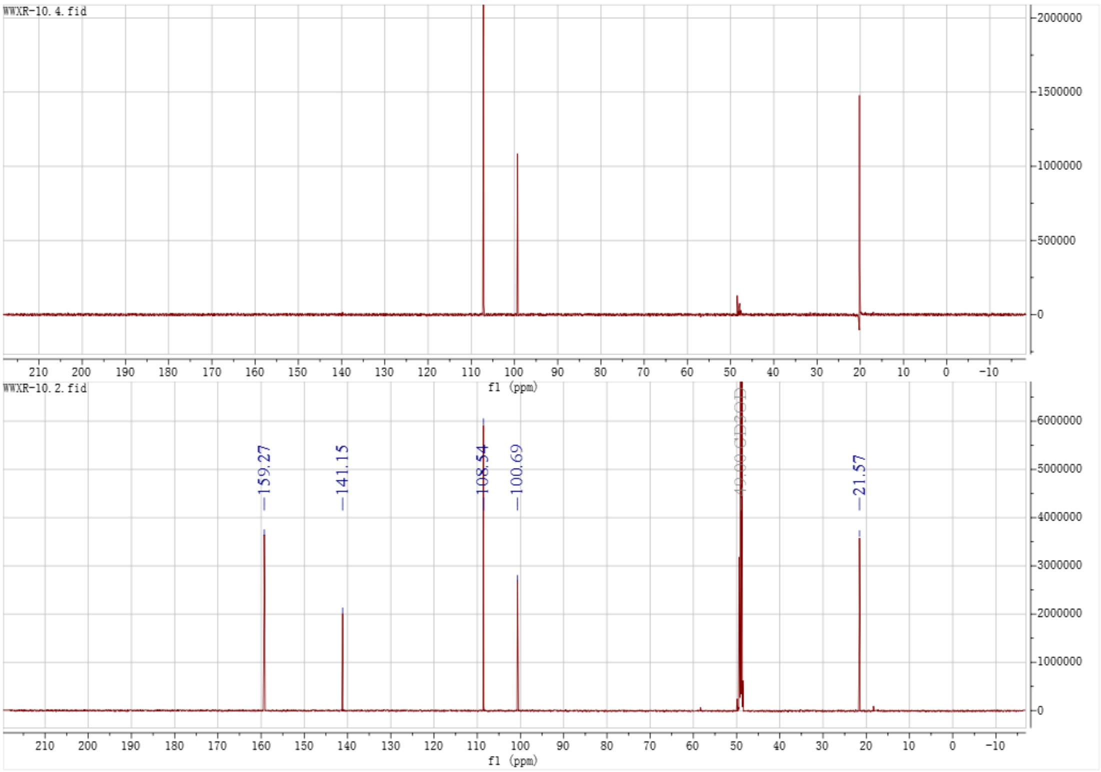

$^{1}$H-NMR spectrum of compound **16** (600 MHz, $CD_3OD$)

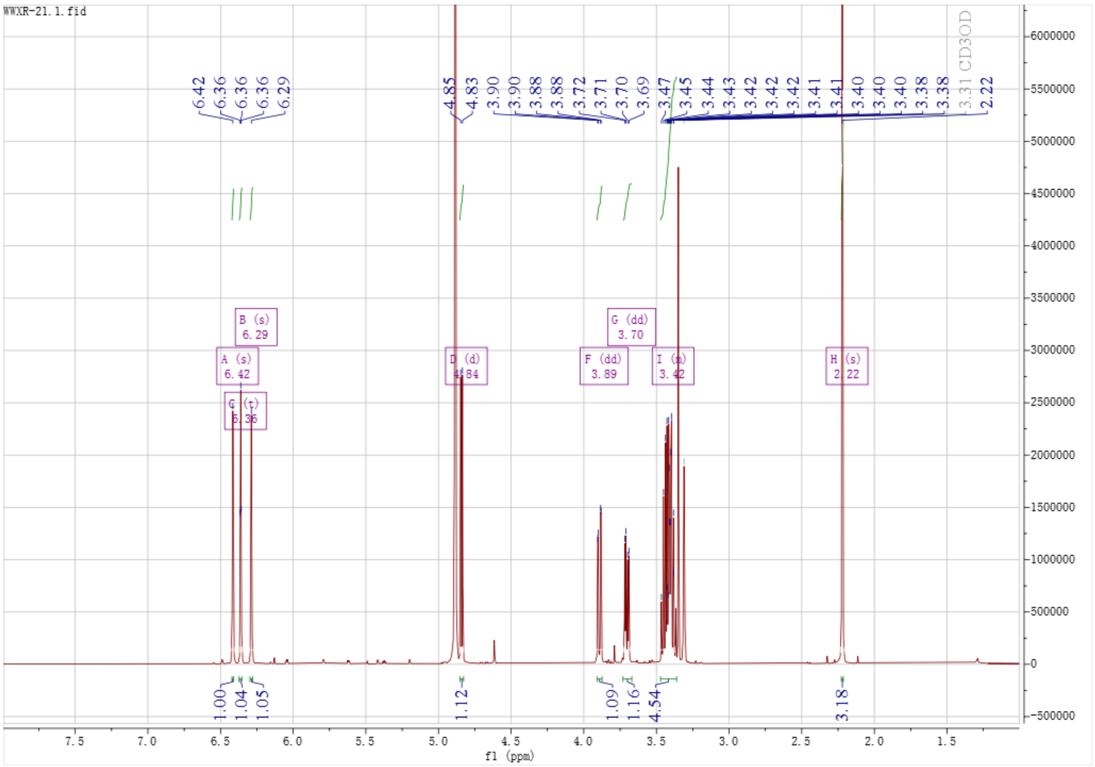


$^{16}$C-NMR and $^{16}$C DEPT-135 spectra of compound **16** (151 MHz, $CD_3OD$)

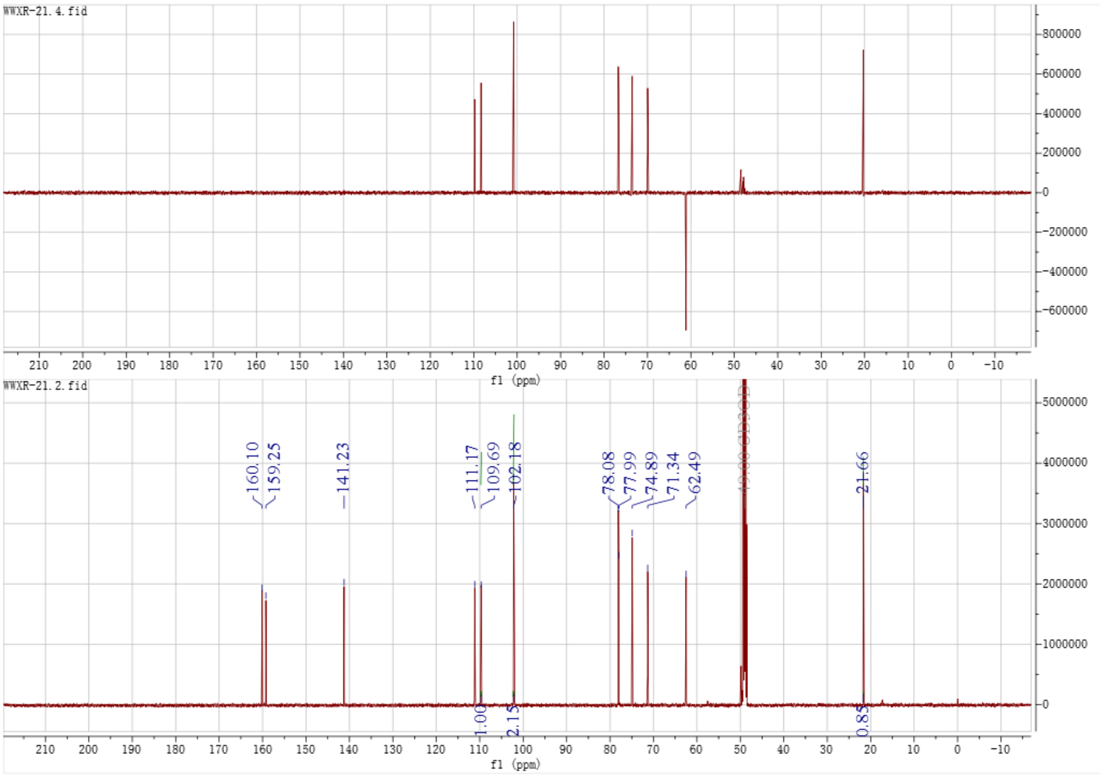

$^{1}$H-NMR spectrum of compound **17** (600 MHz, $CD_3OD$)

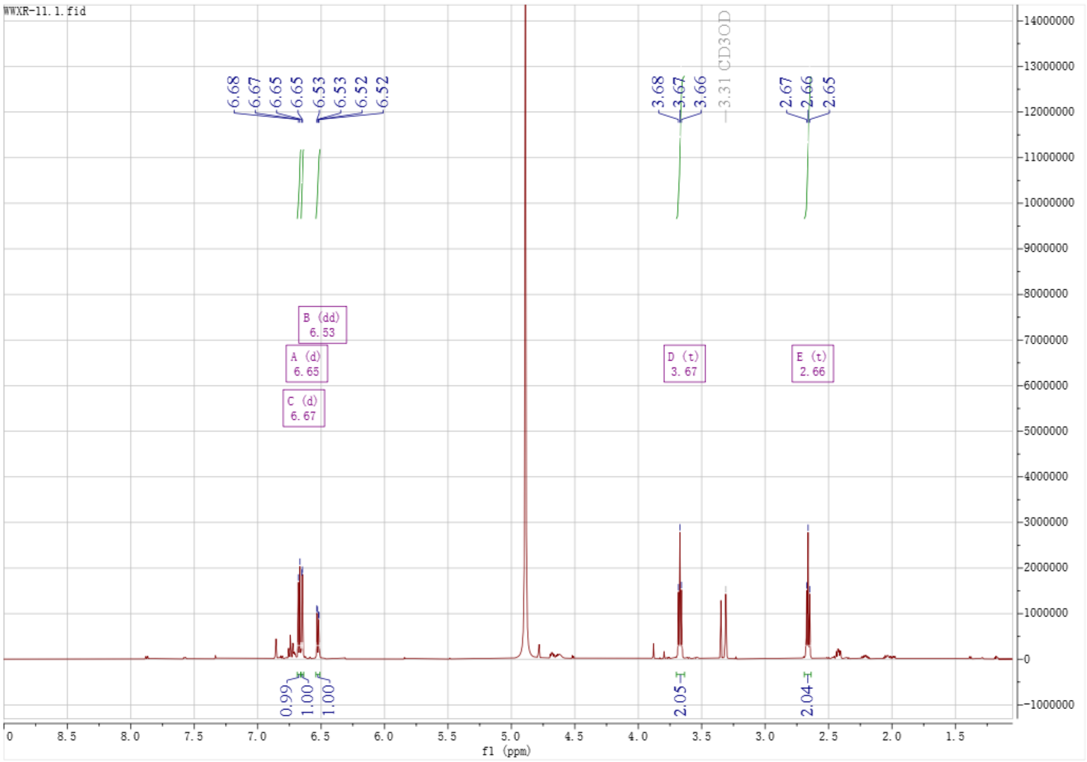


$^{13}$C-NMR and $^{13}$C DEPT-135 spectra of compound **17** (151 MHz, $CD_3OD$)

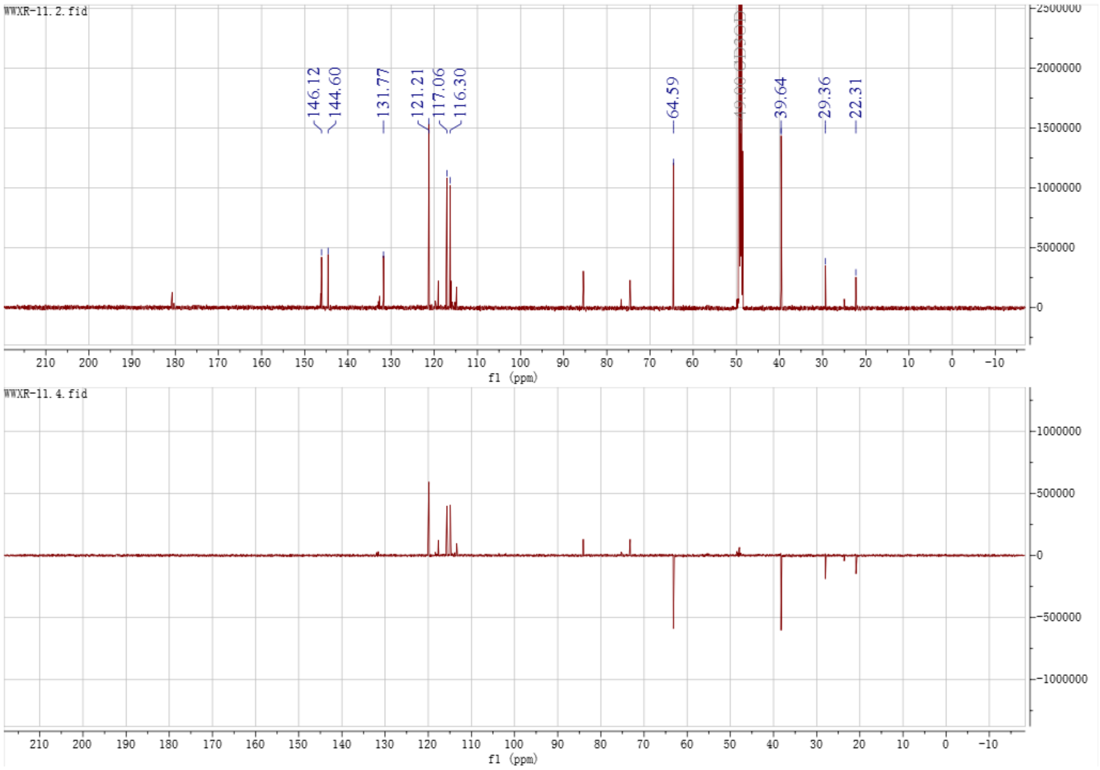

$^1$H-NMR spectrum of compound **18** (600 MHz, $CD_3OD$)

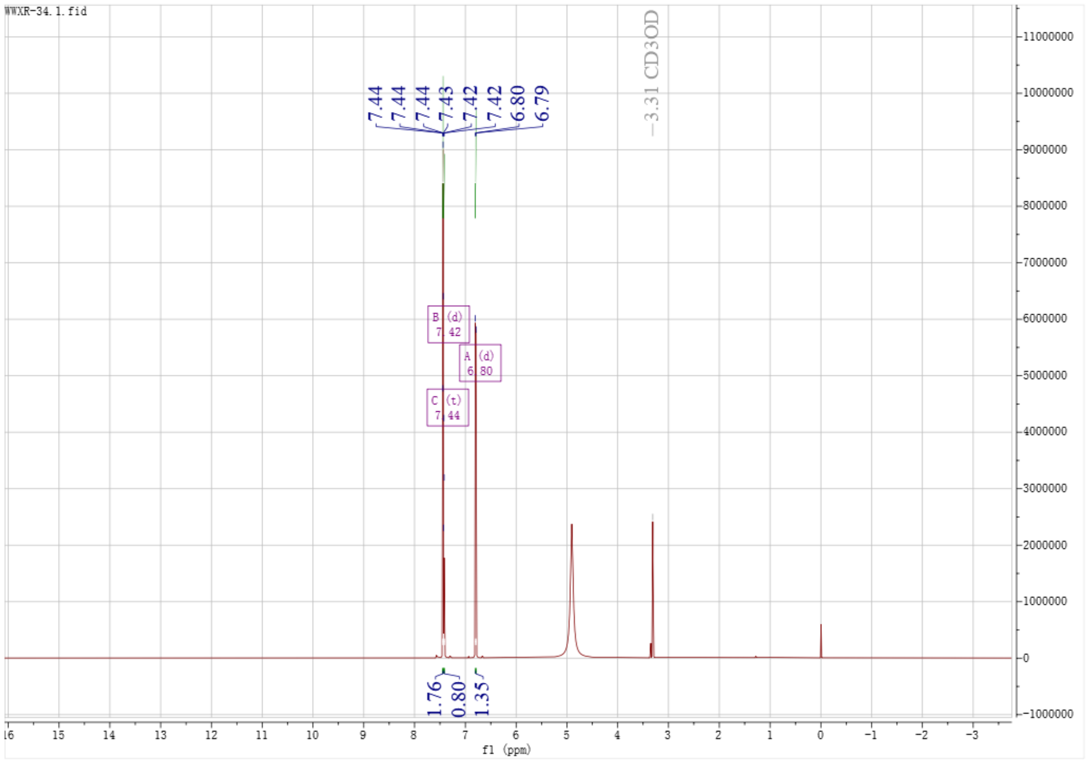


$^{13}$C-NMR and $^{13}$C DEPT-135 spectra of compound **18** (151 MHz, $CD_3OD$)

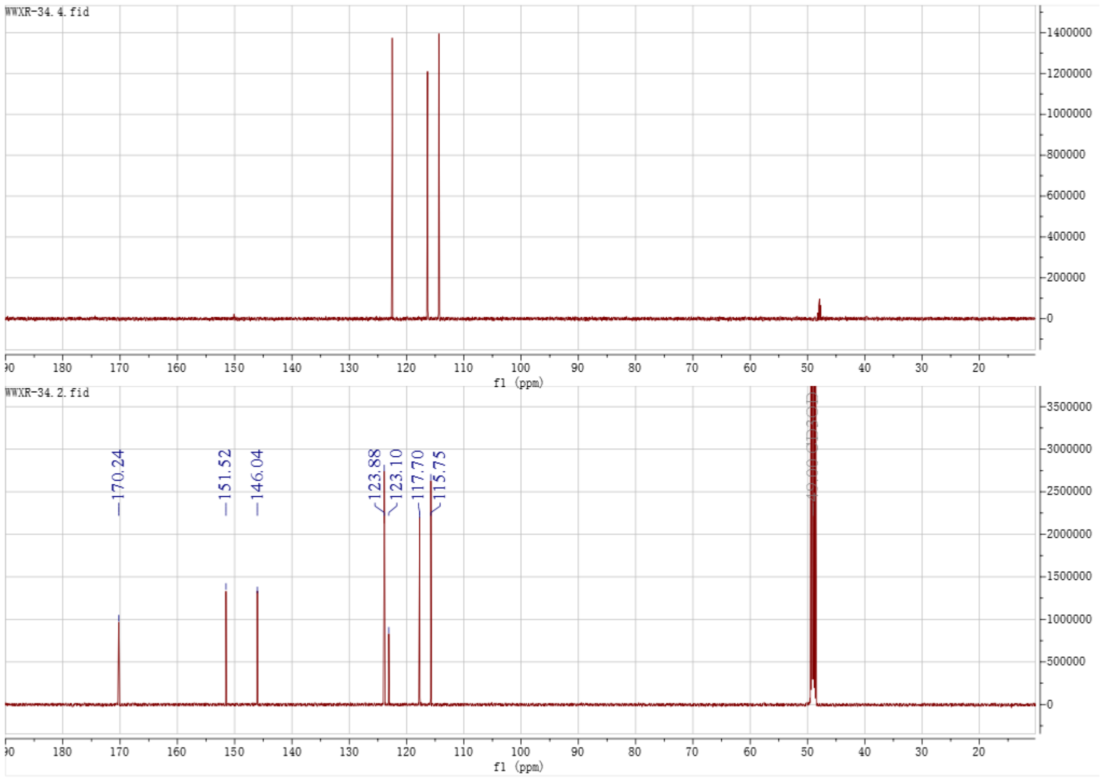

$^{1}$H-NMR spectrum of compound **19** (600 MHz, $CD_3OD$)

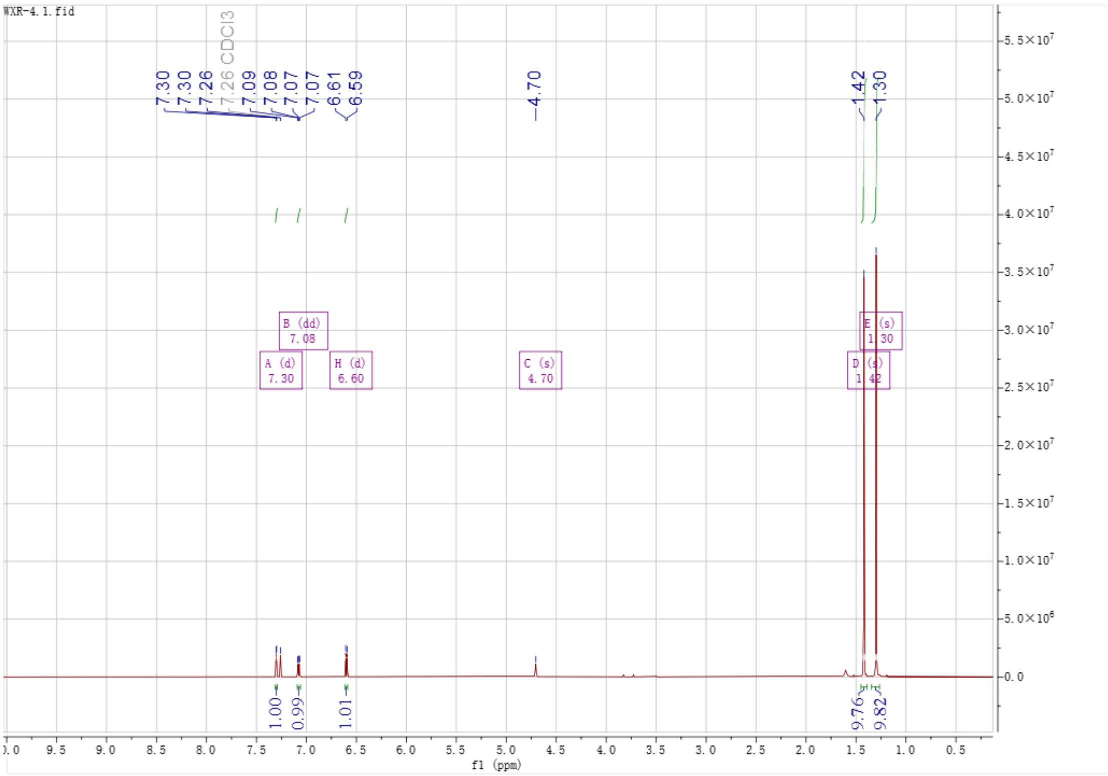


$^{13}$C-NMR and $^{13}$C DEPT-135 spectra of compound **19** (151 MHz, $CD_3OD$)

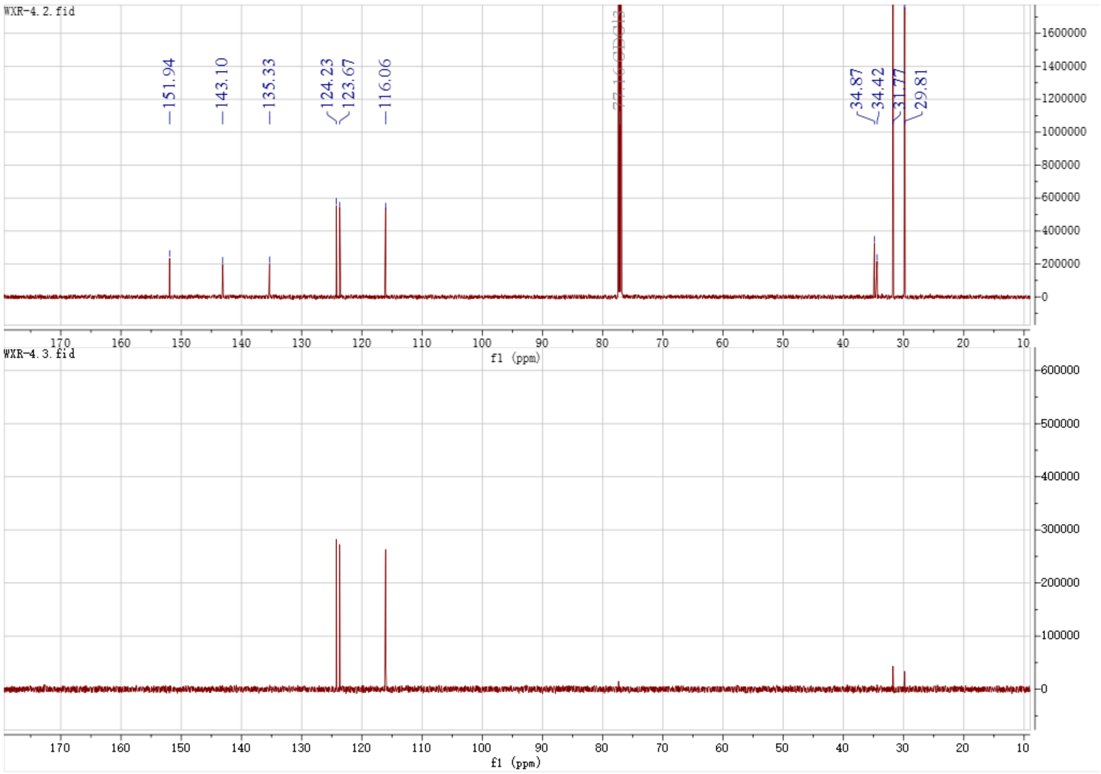

$^1$H-NMR spectrum of compound **20** (600 MHz, $CD_3OD$)

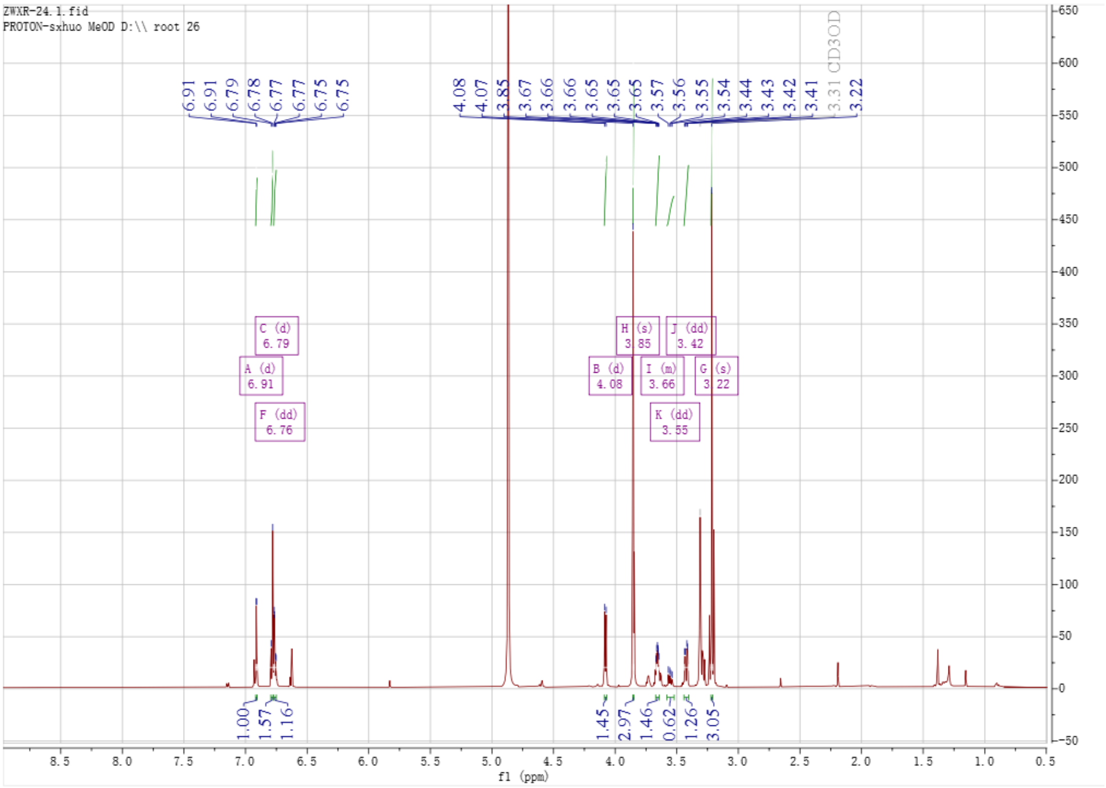

$^{13}$C-NMR and $^{13}$C DEPT-135 spectra of compound **20** (151 MHz, $CD_3OD$)

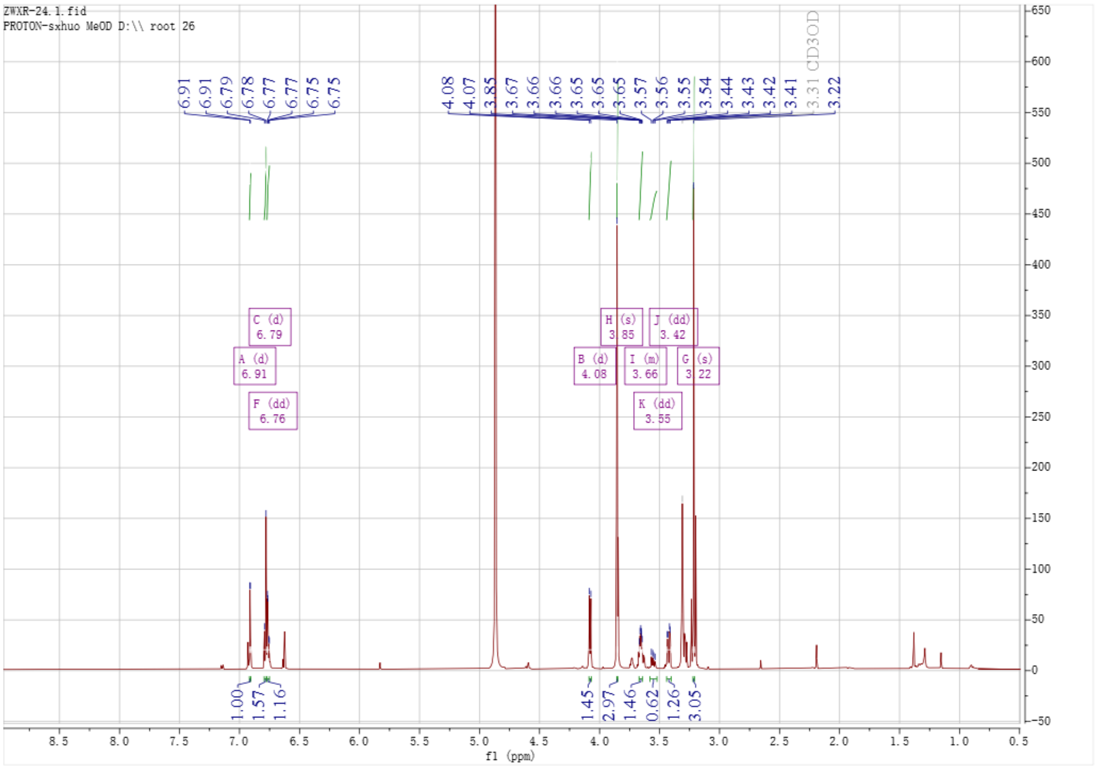

$^{1}$H-NMR spectrum of compound **21** (600 MHz, $CD_3OD$)

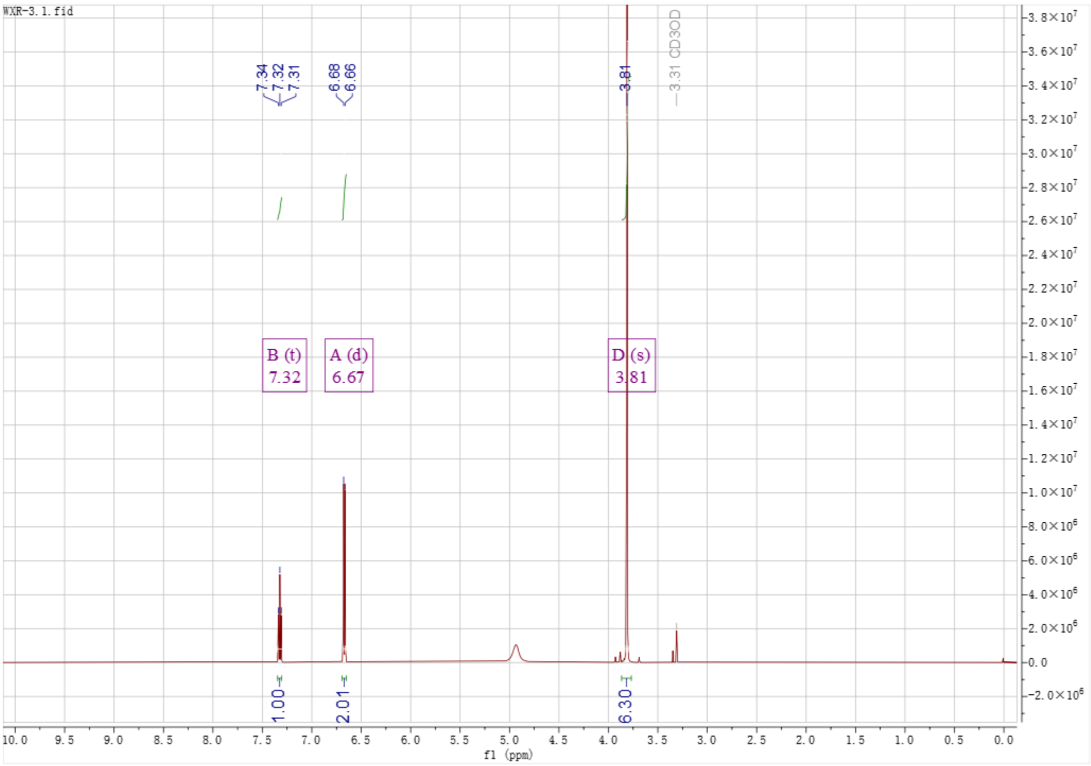


$^{13}$C-NMR and $^{13}$C DEPT-135 spectra of compound **21** (151 MHz, $CD_3OD$)

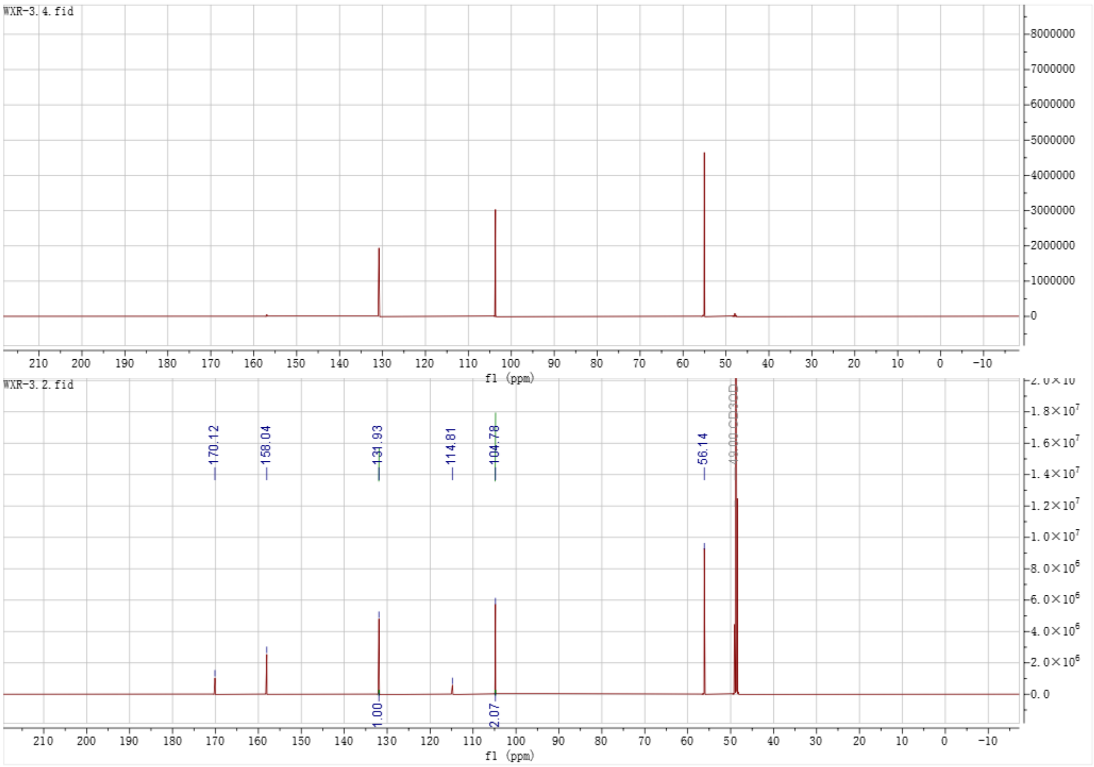

$^{1}$H-NMR spectrum of compound **22** (600 MHz, $CD_3OD$)

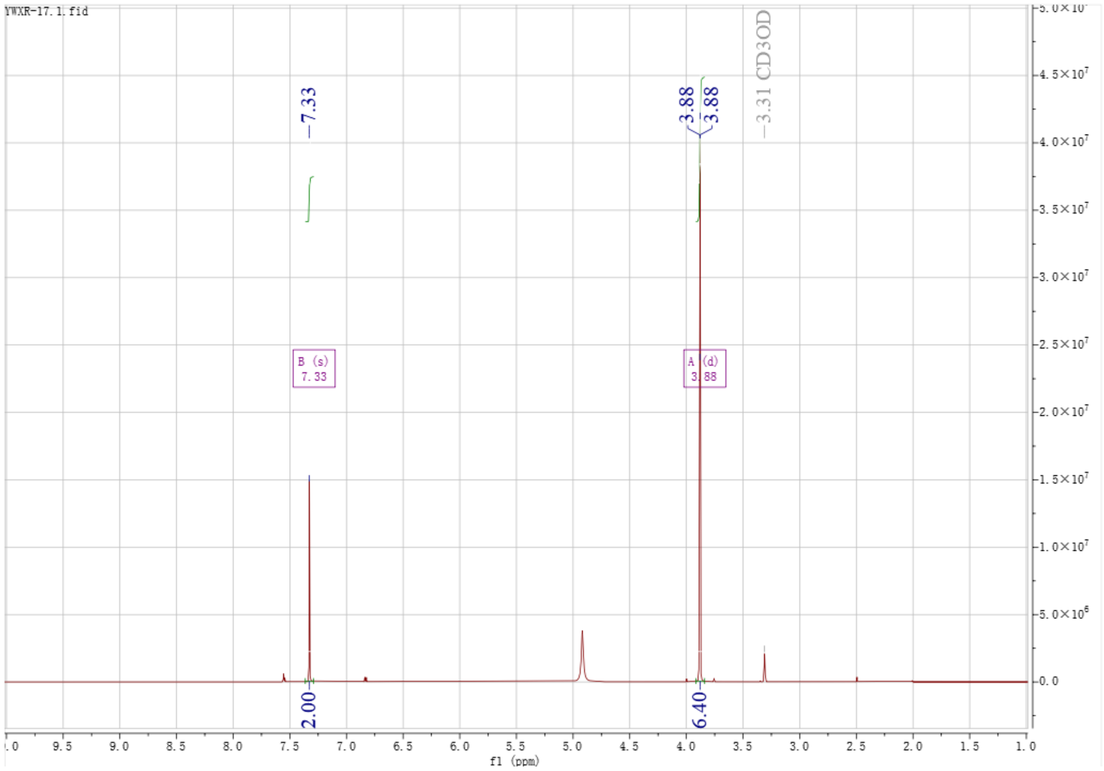


13C-NMR and 13C DEPT-135 spectra of compound 22 (151 MHz, CD3OD)

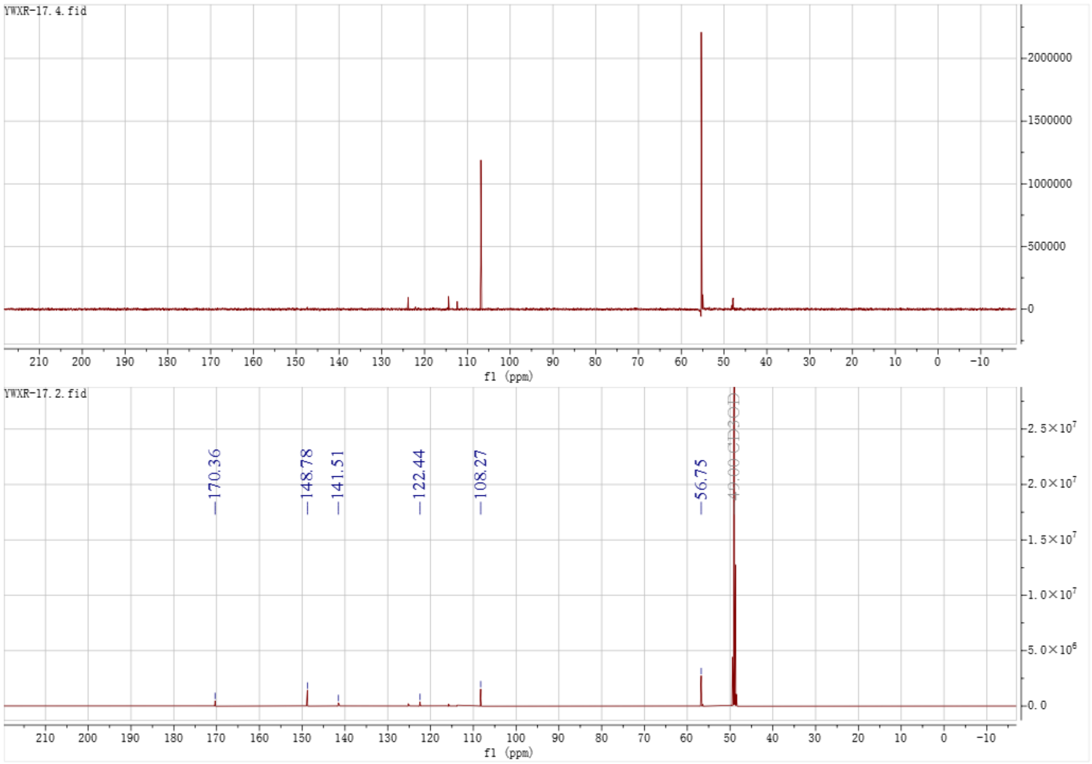

$^{1}$H-NMR spectrum of compound **23** (600 MHz, $CD_3OD$)

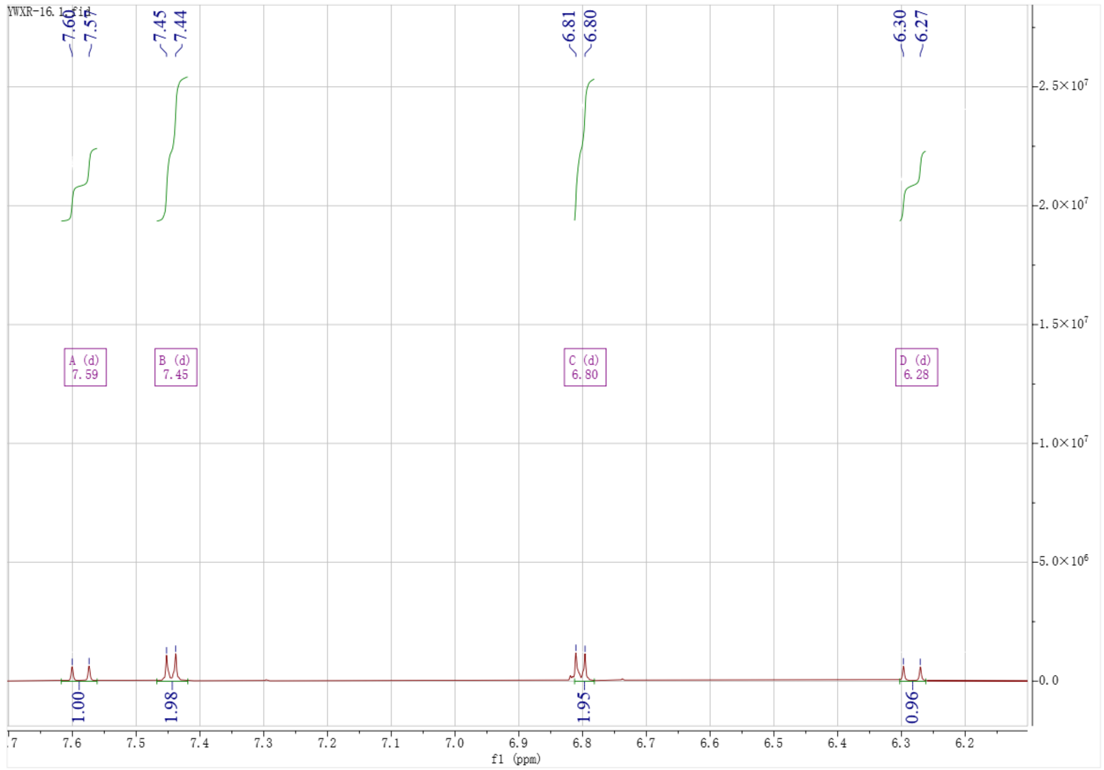


$^{13}$C-NMR and $^{13}$C DEPT-135 spectra of compound **23** (151 MHz, $CD_3OD$)

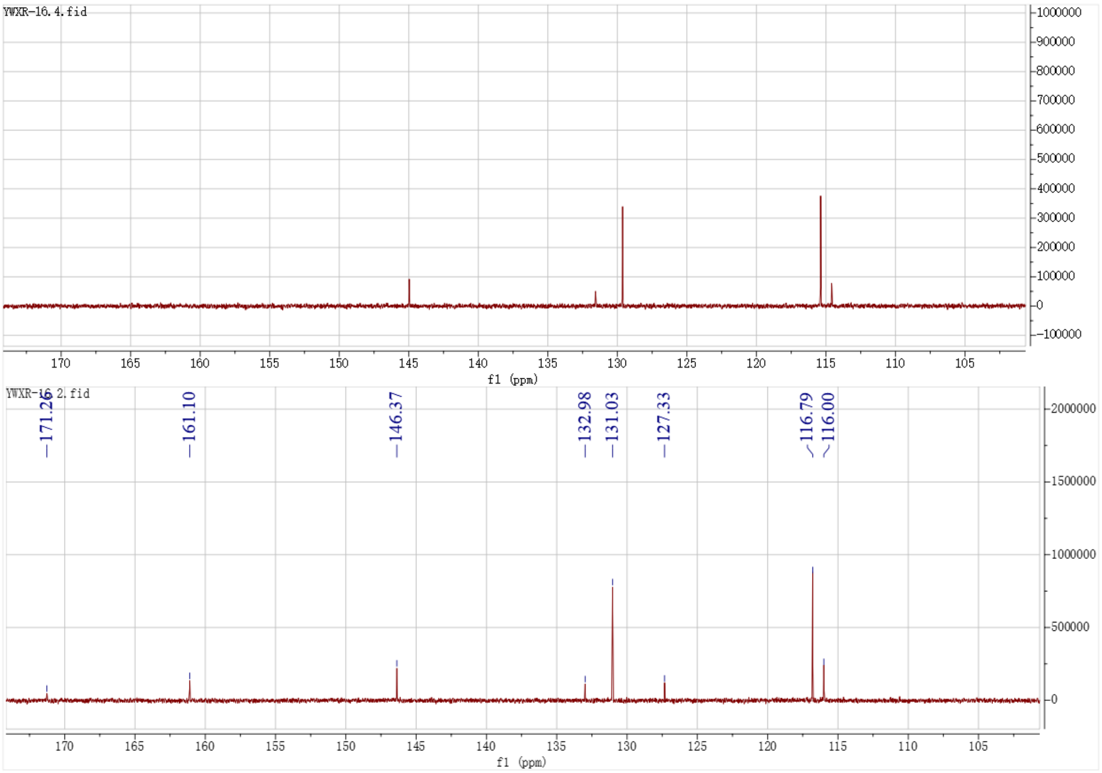

$^{1}$H-NMR spectrum of compound **24** (600 MHz, $CD_3OD$)

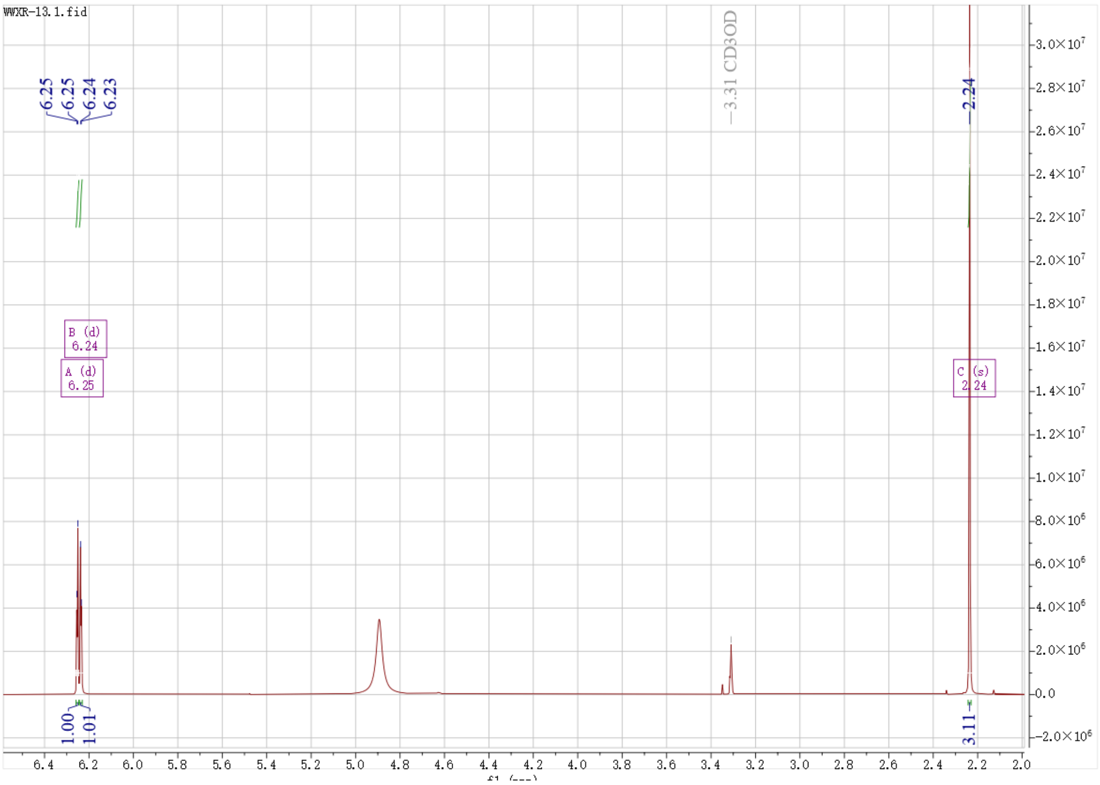

$^{13}$C-NMR and $^{13}$C DEPT-135 spectra of compound **24** (151 MHz, $CD_3OD$)

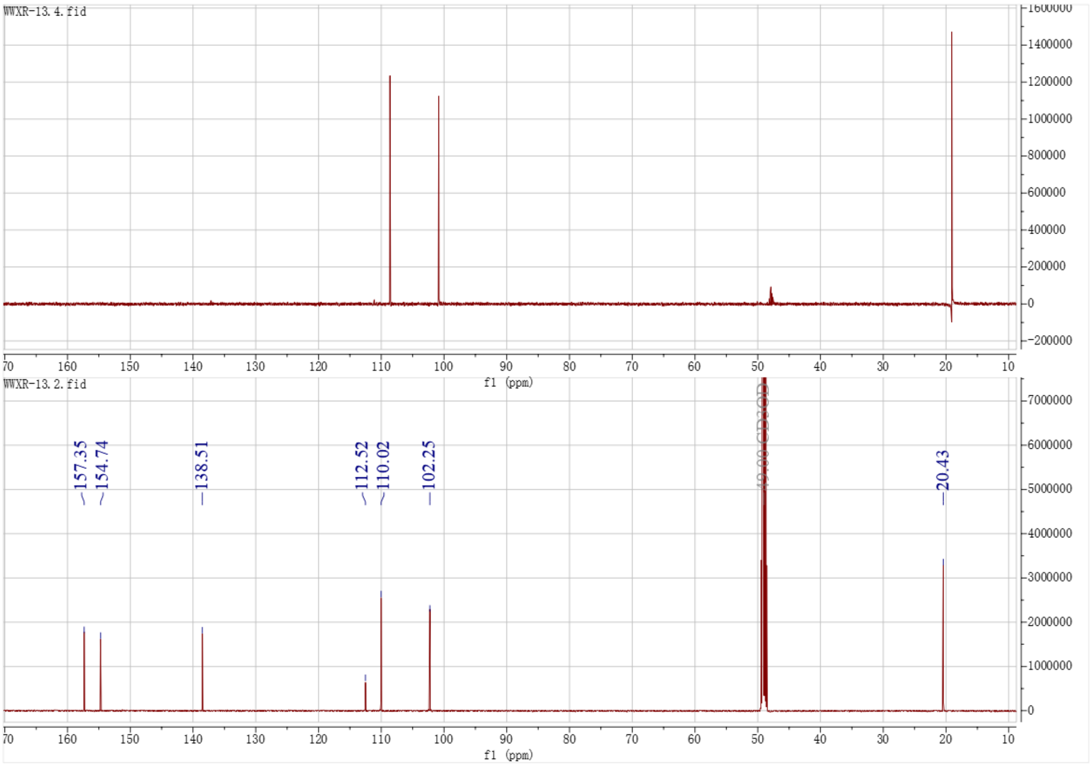

$^1$H-NMR spectrum of compound **25** (600 MHz, $CD_3OD$)

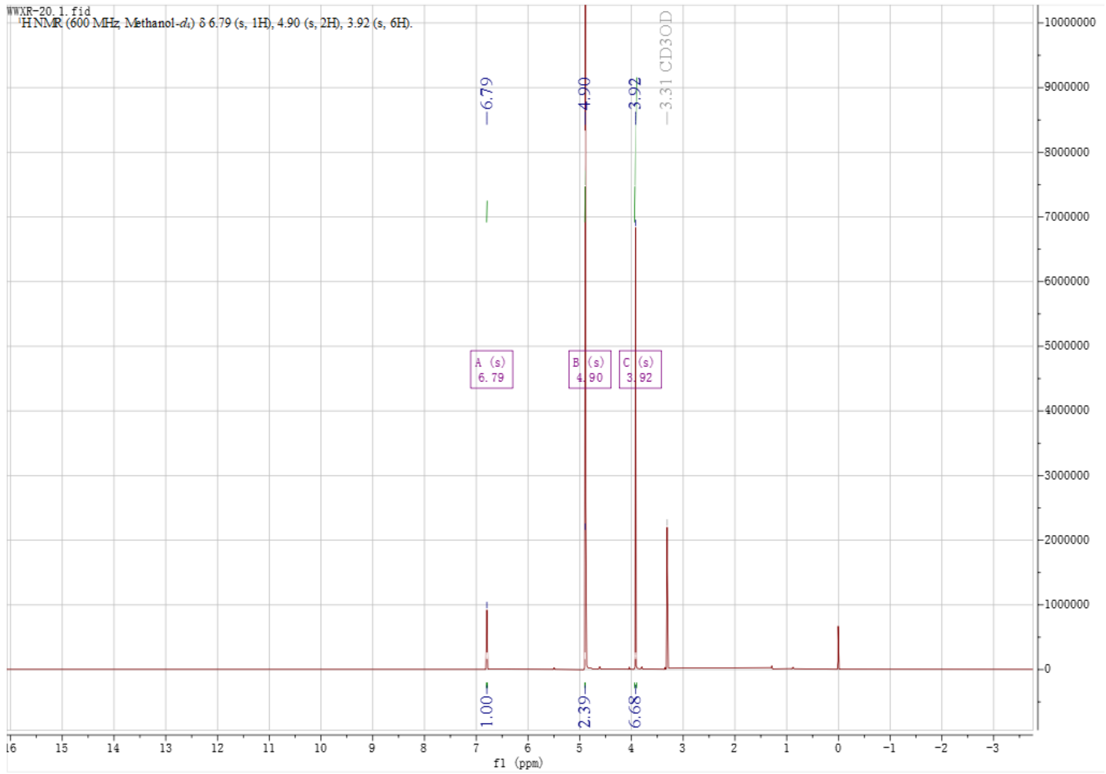


$^{13}$C-NMR and $^{13}$C DEPT-135 spectra of compound **25** (151 MHz, $CD_3OD$)

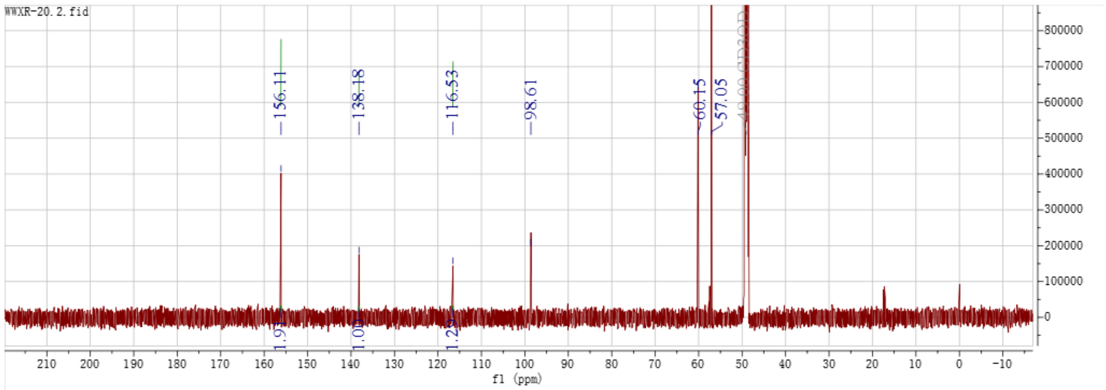


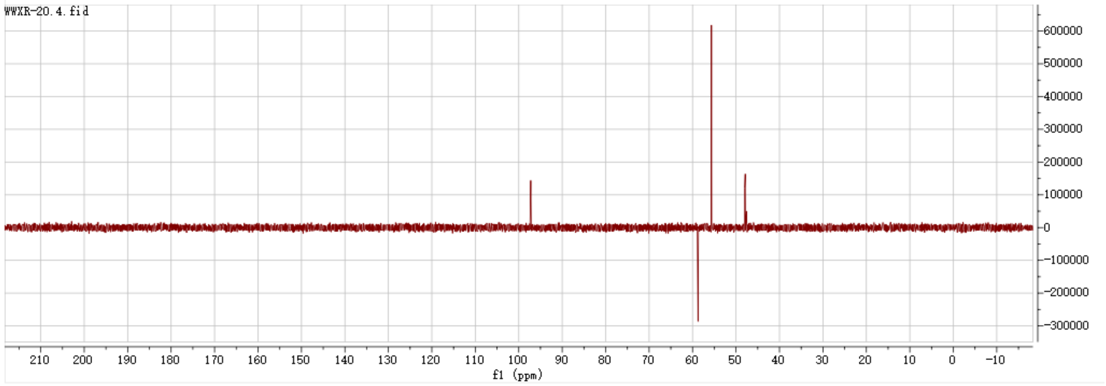

$^{1}$H-NMR spectrum of compound **26** (600 MHz, $CD_3OD$)

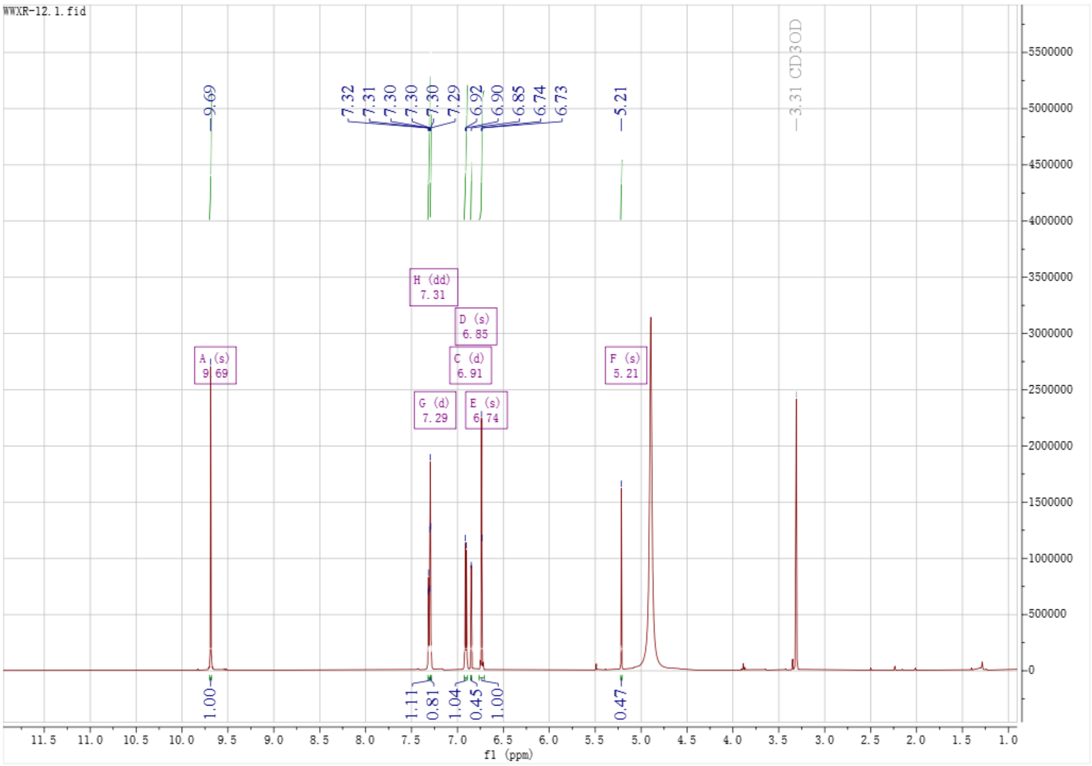

$^{13}$C-NMR and $^{13}$C DEPT-135 spectra of compound **26** (151 MHz, $CD_3OD$)

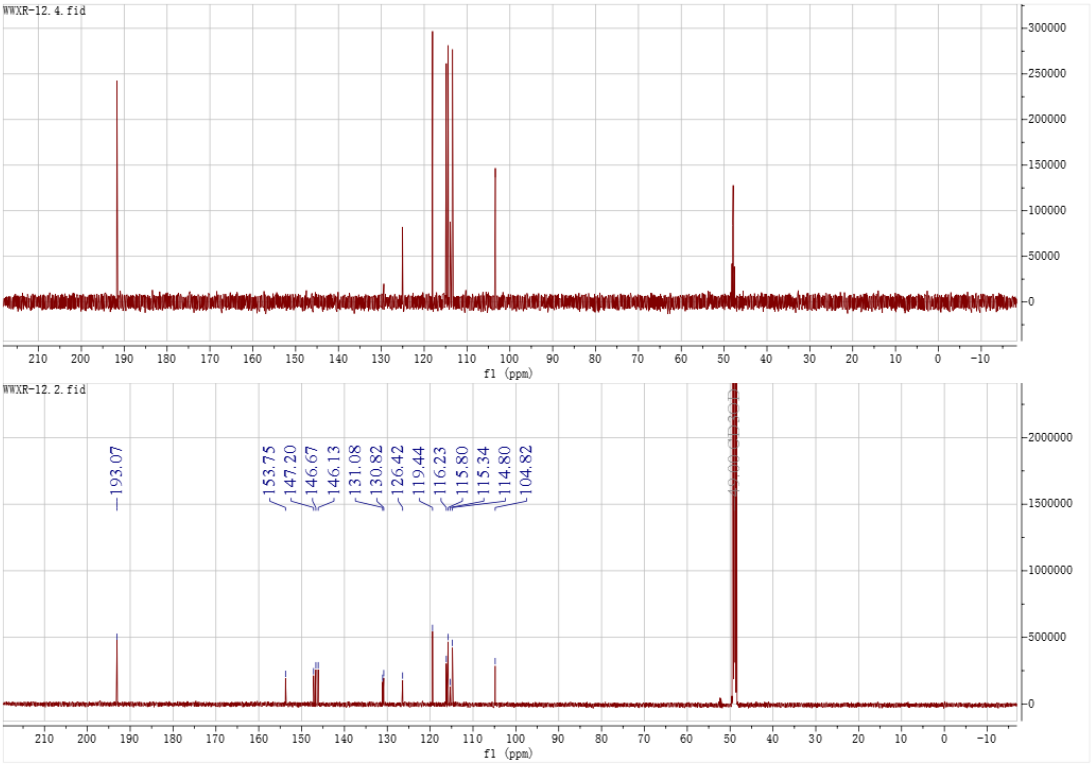

$^{1}H$ and $^{13}C$ NMR data of compounds **8-26**

Crassifogenin A (**8**): $^{1}H$ NMR (600 MHz, $CD_3OD$) $\delta$ 9.50 (s, 1H, H-6), 7.43 (s, 1H, H-4), 7.11 (d, $J$ = 2.1 Hz, 1H, H-2′), 7.03 (dd, $J$ = 8.4, 2.1 Hz, 1H, H-6′), 6.82 (d, $J$ = 2.1 Hz, 1H, H-2″), 6.81 (d, $J$ = 8.2 Hz, 1H, H-5″), 6.74 (d, $J$ = 1.9 Hz, 1 H, H-5′), 6.73 (d, $J$ = 2.1 Hz, 1H, H-6″). $^{13}C$ NMR (151 MHz, $CD_3OD$) $\delta$ 178.88 (C=O), 156.24(C-2), 151.56 (C-5), 148.42(C-4′), 146.67 (C-4″), 146.50(C-3′), 146.45 (C-3″), 125.53(C-4), 125.28(C-1′), 122.56(C-1″), 121.82(C-6″), 120.82(C-6′), 116.85(C-2″), 116.77(C-5″), 116.42(C-2′), 115.21(C-5′).

Breviscapin B (**9**): $^{1}H$ NMR (600 MHz, $CD_3OD$) $\delta$ 6.84 (d, J = 2.1 Hz, 1H, H-2’), 6.82 (d, J = 2.1 Hz, 1H, H-6″), 6.72 (t, J = 2.1 Hz, 1H, H-2″), 6.70 (d, J = 2.2 Hz, 1H, H-6′), 6.69 (d, J = 8.3 Hz, 1H, H-5′), 6.65 (d, J = 8.2 Hz, 1H, H-5″), 4.1 5 – 4.12 (m, 1H, H-2), 3.61 (dd, 1H, $J$ = 11.3, 5.9 Hz, 1H, H-1), 3.54 (dd, $J$ = 11. 3, 5.1 Hz, 1H, H-1), 2.54 – 2.49 (m, 1H, H-4), 2.38 – 2.33 (m, 1H, H-4), 1.96 – 1. 92 (m, 1H, H-3), 1.78 – 1.74 (m, 1H, H-3). $^{13}C$ NMR (151 MHz, $CD_3OD$) $\delta$ 145.83 (C-4′), 145.58(C-3′), 144.95(C-4″), 144.93(C-3″), 140.09(C-1′), 139.33(C-1″), 118.70(C-6′), 118.56(C-6″), 115.72(C-5′), 115.55(C-5″), 115.07(C-2″), 114.85(C-2′), 89.79(C-5), 8 0.44(C-2), 66.29(C-1), 39.47(C-4), 29.04(C-3).

Crassifogenin B (**10**): $^{1}H$ NMR (600 MHz, $CD_3OD$) $\delta$ 7.34 (d, $J$ = 2.1 Hz, 1H, H-2′), 7.22 (dd, $J$ = 8.3, 2.1 Hz, 1H, H-6′), 7.06 (s, 1H, H-8), 7.04 (d, $J$ = 2.5 Hz, 1H, H-5), 7.00 (s, 1H, H-4), 6.86 (d, $J$ = 2.4 Hz, 1H, H-2), 6.81 (d, $J$ = 8.3 Hz, 1 H, H-5′). $^{13}C$ NMR (151 MHz, $CD_3OD$) $\delta$ 199.53(C=O), 153.63(C-3), 152.84(C-4′), 14 8.56(C-7), 146.43(C-6), 146.21(C-3′), 137.95(C-1), 133.86(C-10), 130.60(C-1′), 125.81(C -6′), 122.59(C-9), 117.97(C-2), 116.86(C-2′), 115.86(C-5′), 111.58(C-4), 109.61(C-5), 10 8.74(C-8).

Sinensigenin C (**11**): $^{1}H$ NMR (600 MHz, $CD_3OD$) $\delta$ 6.89 (s, 1H, 2′), 6.88 (d, $J$ = 2.0 Hz, 1H, H-2″), 6.80 (dd, $J$ = 8.2, 2.0 Hz, 1H, H-6″), 6.77 (d, $J$ = 8.1 Hz, 1H, H-5″), 6.00 (s, 1H, H-5′), 5.08 (d, $J$ = 5.2 Hz, 1H, H-1), 4.58 – 4.53 (m, 1H, H-2), 2.30 – 2.27 (m, 1H, H-4), 2.26 – 2.22 (m, 1H,H-5), 2.07 – 2.00 (m, 1H,H-3). $^{13}C$ NMR (151 MHz, $CD_3OD$) $\delta$ 145.95(C-4″), 145.93(C-3″), 145.45(C-3′), 144.85(C-4′), 1 36.57(C-6′), 135.74(C-1″), 128.38(C-1′), 120.12(C-6″), 116.25(C-2″), 115.64(C-5″),114.6 4(C-2′), 113.02(C-5′), 87.54(C-5), 79.38(C-2), 70.27(C-1), 39.66(C-4), 23.40(C-3).

Sinensigenin A (**12**): $^{1}H$ NMR (600 MHz, $CD_3OD$) $\delta$ 6.91 (d, $J$ = 2.1 Hz, 1H, H-2″), 6.84 (dd, $J$ = 8.3, 2.2 Hz, 1H, H-6″), 6.78 (s, 1H, H-5′), 6.77 (d, $J$ = 8.3 Hz, 1H, H-5″), 6.29 (s, 1H, H-2′), 3.92 (ddd, $J$ = 10.1, 5.7, 4.0 Hz, 1H, ), 2.31 – 2.22

(m, 1H), 2.07 (qd, *J* = 14.8, 13.8, 6.5 Hz, 1H), 1.97 (ddd, *J* = 13.2, 5.9, 1.7 Hz, 1 H), 1.80 (dtt, *J* = 12.7, 5.5, 1.5 Hz, 1H), 0.83 (tdd, *J* = 12.6, 10.6, 5.8 Hz, 1H). $^{13}$C NMR (151 MHz, $CD_3OD$) $\delta$ 146.11(C-3″), 146.02(C-4″),, 145.99(C-4′), 145.43(C-3′), 138.47(C-6′), 134.95(C-1″), 132.27(C-1′), 118.86(C-6″), 115.79(C-5′), 115.12(C-2″), 110. 31(C-5″), 109.01(C-2′), 87.61(C-1), 82.49(C-2), 67.34(C-5), 32.61(C-4), 27.57(C-3).

(+)-Syringaresinol-O-β-D-glucopyranoside (**13**): $^{1}$H NMR (600 MHz, $CD_3OD$) $\delta$ 6. 71 (s, 1H), 6.65 (s, 1H), 4.85 (d, *J* = 1.5 Hz, 1H), 4.76 (d, *J* = 4.0 Hz, 0H), 4.71 (d, *J* = 4.4 Hz, 1H), 4.31 – 4.24 (m, 1H), 3.91 (dd, *J* = 9.1, 2.3 Hz, 1H), 3.85 (s, 4H), 3.84 (s, 3H), 3.77 (dd, *J* = 12.0, 2.4 Hz, 1H), 3.66 (dd, *J* = 12.0, 5.1 Hz, 1H), 3.52 – 3.46 (m, 1H), 3.43 – 3.39 (m, 1H), 3.20 (ddt, *J* = 7.5, 5.3, 2.5 Hz, 1H), 3.15 – 3.10 (m, 1H). $^{13}$C NMR (151 MHz, $CD_3OD$) $\delta$ 154.41(C-3″, 5″), 149.35(C-3′, 5′), 139.55(C-1″), 136.22(C-4′), 135.58(C-4″), 133.07(C-1′), 105.33(C-1‴), 104.83(C-2″, 6″), 104.51(C-2′, 6′), 87.57(C-6), 87.17(C-2), 78.32(C-5‴), 77.81(C-3‴), 75.70(C-2‴), 72.9 1(C-4), 72.85(C-8), 71.32(C-4‴), 62.57(C-6‴), 57.07(C-3″, 5″ -$OCH_3$), 56.81(C-3′, 5′ -$OCH_3$), 55.70(C-5), 55.49(C-1).

(1*R*, 2*R*)-crassifogenin D (**14**): $^{1}$H NMR (500 MHz, $CD_3OD$) $\delta$ 6.83 (d, *J* = 2.0 Hz, 1H, H-2′), 6.82 (d, *J* = 1.9 Hz, 1H, H-2″), 6.76 (d, *J* = 8.0 Hz, 1H, H-5′), 6.75 (dd, *J* = 8.0, 1.9 Hz, 1H, H-6′), 6.70 (dd, *J* = 8.1, 2.0 Hz, 1H, H-6″), 6.67 (d, *J* = 8.1 Hz, 1H, H-5″), 4.09 (d, *J* = 5.9 Hz, 1H, H-1), 3.85 (q, *J* = 5.9 Hz, 1H, H-2), 3. 23 (s, 3H, -$OCH_3$), 2.55 (d, *J* = 2.9 Hz, 1H, H-3a), 2.54 (d, *J* = 2.0 Hz, 1H, H-3b). $^{13}$C NMR (126 MHz, $CD_3OD$) $\delta$ 146.78(C-4″), 146.24(C-4′), 146.18(C-3″), 146.00(C-3′), 131.04(C-1′), 124.85(C-6″), 120.97(C-6′), 119.48(C-2″), 116.45(C-1″), 116.20(C-5″), 116.03 (C-5′), 115.98(C-2′), 86.85(C-1), 84.93(C-4), 83.22(C-5), 74.31(-$OCH_3$), 57.04(C -2), 24.84(C-3).

Orcinol (**15**): $^{1}$H NMR (600 MHz, $CD_3OD$) $\delta$ 6.11 (d, *J* = 1.2 Hz, 2H, H-4, 6), 6.06 (t, *J* = 2.2 Hz, 1H, H-2), 2.17 (s, 3H, H-7).$^{13}$C NMR (151 MHz, $CD_3OD$) $\delta$ 15 9.27 (C-1, 3), 141.15 (C-5), 108.54 (C-4, 6), 100.69 (C-2), 21.57 (C-7).

Orcinol glucoside (**16**): $^{1}$H NMR (600 MHz, $CD_3OD$) $\delta$ 6.42 (s, 1H, H-2), 6.36 (s, *J* = 2.2 Hz, 1H, H-4), 6.29 (s, 1H, H-6), 4.84 (d, *J* = 7.4 Hz, 2H, H-1′), 3.89 (d d, *J* = 12.1, 2.1 Hz, 2H, Hb-6′), 3.70 (dd, *J* = 12.1, 5.2 Hz, 2H, Ha-6′), 3.48 – 3.37 (m, 4H, Glc H), 2.22 (s, 3H, $CH_3$). $^{13}$C NMR (151 MHz, $CD_3OD$) $\delta$ 160.10 (C-1), 159.25 (C-3), 141.23 (C-5), 111.17 (C-6), 109.69 (C-4), 102.18 (C-2,C-1′), 78.08 (C-5′), 77.99 (C-3′), 74.89 (C-2′), 71.34 (C-4′), 62.49 (C-6′), 21.66 ($CH_3$).

3,4-dihydroxyphenylethyl alcohol (**17**): $^{1}$H NMR (600 MHz, $CD_3OD$) $\delta$ 6.67 (d, *J* = 8.0 Hz, 1H, H-5), 6.65 (d, *J* = 2.1 Hz, 1H, H-2), 6.53 (dd, *J* = 8.0, 2.1 Hz, 1H, H-6), 3.67 (t, *J* = 7.3 Hz, 2H, H-8), 2.66 (t, *J* = 7.3 Hz, 2H, H-7).$^{13}$C NMR (151 M Hz, $CD_3OD$) $\delta$ 146.12 (C-3), 144.60 (C-4), 131.77 (C-1), 121.21 (C-6), 117.06 (C-5), 116.30 (C-2), 64.59 (C-8), 39.64 (C-7).

Protocatechuic acid (**18**): $^{1}$H NMR (600 MHz, $CD_3OD$) $\delta$ 7.44 (t, *J* = 1.8 Hz, 1 H, H-2), 7.42 (d, *J* = 2.1 Hz, 1H, H-6), 6.80 (d, *J* = 8.1 Hz, 1H, H-5). $^{13}$C NMR (151 MHz, $CD_3OD$) $\delta$ 170.24 (C-7), 151.52 (C-3), 146.04 (C-4), 123.88 (C-1), 123.10 (C-6), 117.70 (C-2), 115.75 (C-5).

2,4-Di-tert-butylphenol (**19**): $^{1}$H NMR (600 MHz, Chloroform-*d*) $\delta$ 7.30 (d, *J* = 2. 4 Hz, 1H, H-3), 7.08 (dd, *J* = 8.2, 2.4 Hz, 1H, H-5), 6.60 (d, *J* = 8.2 Hz, 1H, H-6), 4.70 (s, 1H, 1-OH), 1.42 (s, 9H, 7-t-Bu), 1.30 (s, 9H, 8-t-Bu).$^{13}$C NMR (151 MHz, $CDCl_3$) $\delta$ 151.94 (C-1), 143.10 (C-4), 135.33 (C-2), 124.23 (C-3), 123.67 (C-5), 116.0 6 (C-6), 34.87 (c-7), 34.42 (c-8), 31.77 (C-8-$(CH_3)_3$), 29.81 (C-7-$(CH_3)_3$).

Threo-5-hydroxy-3,7-dimethoxyphenylpropane-8,9-diol (**20**): $^{1}$H NMR (600 MHz, C $D_3OD$) $\delta$ 6.91 (d, *J* = 1.8 Hz, 1H, H-2), 6.79 (d, *J* = 8.1 Hz, 1H, H-4), 6.76 (dd, *J* = 8.0, 1.8 Hz, 1H, H-6), 4.08 (d, *J* = 6.8 Hz, 1H, H-7), 3.85 (s, 3H, -OCH3), 3.67 – 3.64 (m, 1H, H-8), 3.55 (dd, *J* = 11.3, 6.5 Hz, 1H, H-9), 3.42 (dd, *J* = 11.3, 3.8 Hz, 1H, H-9′), 3.22 (s, 3H, -OCH3). $^{13}$C NMR (151 MHz, $CD_3OD$) $\delta$ 149.14(C-3), 1 47.54(C-5), 131.50(C-1), 121.58(C-4), 116.03(C-6), 111.81(C-2), 85.58(C-7), 77.09(C-8), 63.92(C-9), 56.89(-OCH3), 56.33(-OCH3).

2,6-Dimethoxy-benzioc acid (**21**): $^{1}$H NMR (600 MHz, $CD_3OD$) $\delta$ 7.32 (t, *J* = 8. 5 Hz, H-4), 6.67 (d, *J* = 8.6 Hz, 2H, H-3, 5), 3.81 (s, 4H, 2, 6-OMe). $^{13}$C NMR (15 1 MHz, $CD_3OD$) $\delta$ 170.12 (C=O), 158.04 (C-2, 6), 131.93(C-4), 114.81(C-1), 104.78 (C-3, 5), 56.14(2, 6-OMe).

Syringic acid (**22**): $^{1}$H NMR (600 MHz, $CD_3OD$) $\delta$ 7.33 (s, 2H, H-2, 6), 3.88 (d, *J* = 0.9 Hz, 6H, -$OCH_3$). $^{13}$C NMR (151 MHz, $CD_3OD$) $\delta$ 170.36(COOH), 148.78(C-2, 6), 141.51(C-1), 122.44(C-4), 108.27(C-3,5), 56.75(-$OCH_3$).

*p*-coumaric acid (**23**): $^{1}$H NMR (600 MHz, $CD_3OD$) $\delta$ 7.59 (d, *J* = 15.9 Hz, 1H, H-7), 7.45 (d, *J* = 8.6 Hz, 2H, H-2,6), 6.80 (d, *J* = 8.6 Hz, 2H, H-3,5), 6.28 (d, *J* = 15.9 Hz, 1H, H-8). $^{13}$C NMR (151 MHz, $CD_3OD$) $\delta$ 171.26(C-9), 161.10(C-4), 146.3 7(C-7), 132.58(C-2), 131.03(C-6), 127.33(C-1), 116.79(C-3/5), 116.00(C-8).

2-Chloro-3,5-dihydroxytoluene (**24**): $^{1}$H NMR (600 MHz, $CD_3OD$) $\delta$ 6.25 (d, $J$ = 2.8 Hz, 1H, H-6), 6.24 (d, $J$ = 2.1 Hz, 1H, H-4), 4.89 (s, 2H, -OH), 2.24 (s, 3H, C H3). $^{13}$C NMR (151 MHz, $CD_3OD$) $\delta$ 157.35 (C-5), 154.74 (C-3), 138.51 (C-1), 112.5 2 (C-2), 110.02 (C-6), 102.25 (C-4), 20.43 (C-7).

2,6-Dichloro-3,5-dimethoxybenzenemethanol (**25**): $^{1}$H NMR (600 MHz, $CD_3OD$) $\delta$ 6.79 (s, 1H, H-4), 4.90 (s, 2H, C-7), 3.92 (s, 6H, $-OH_3$). $^{13}$C NMR (151 MHz, $CD_3O$ D) $\delta$ 156.11(C-1), 138.18(C-3,5), 116.53(C-2,6), 98.61(C-4), 60.15(C-7), 57.05(-OCH3).

2-(3,4-Dihydroxyphenyl)-1,3-benzodioxole-5-carboxaldehyde (**26**): $^{1}$H NMR (600 M Hz, $CD_3OD$) $\delta$ 9.69 (s, 1H, H-10), 7.31 (m, 1H, H-6), 7.29 (d, $J$ = 2.0 Hz, 1H, H-4), 6.91 (d, $J$ = 8.0 Hz, 1H, H-7), 6.85 (s, 1H, H-2′), 6.74 (s, 1H, H-5′), 5.21 (s, 1 H, H-2). $^{13}$C NMR (151 MHz, $CD_3OD$) $\delta$ 193.07 (C-10), 153.75 (C-9), 147.20 (C-1′), 146.67 (C-8), 146.13 (C-3′), 131.08 (C-4′), 130.82 (C-5), 126.42 (C-6), 119.44 (C-6′), 116.23 (C-7), 115.80 (C-5′), 115.34 (C-4), 114.80 (C-2′), 104.82 (C-2).

Effects of compounds **4, 5, 9, 10, 14, 15, 16, 18, 19, 21, 22, 23, 24** and **26** on glutamate-induced oxidative injury of SH-SY5Y cells.

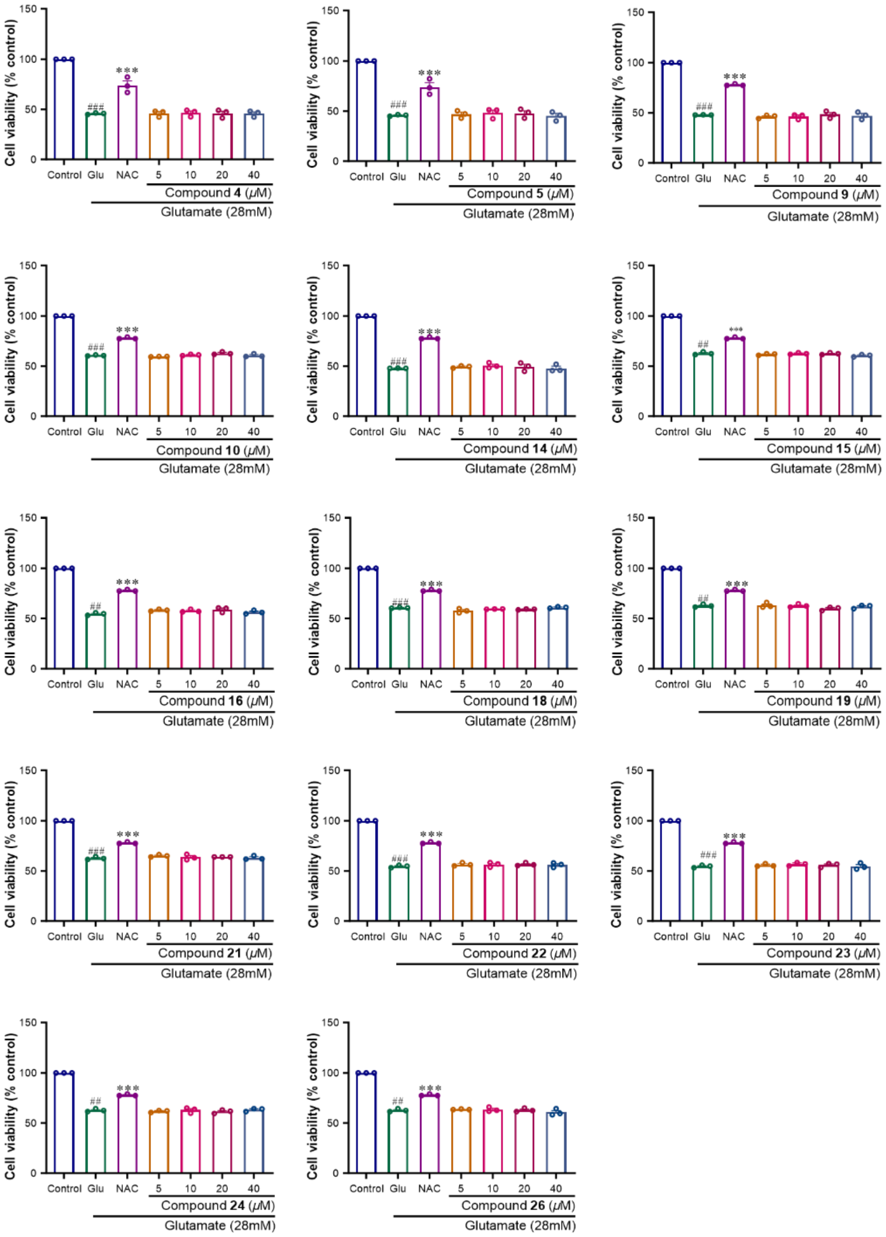

Supplement: Supplementary file 1 [file molecules-29-05648-s001.zip › Supplementary Materials.pdf]
